# AI protein structure prediction-based modeling and mutagenesis of a protostome receptor and peptide ligands reveal key residues for their interaction

**DOI:** 10.1016/j.jbc.2022.102440

**Published:** 2022-08-30

**Authors:** Shi-Qi Guo, Ya-Dong Li, Ping Chen, Guo Zhang, Hui-Ying Wang, Hui-Min Jiang, Wei-Jia Liu, Ju-Ping Xu, Xue-Ying Ding, Ping Fu, Ke Yu, Hai-Bo Zhou, James W. Checco, Jian Jing

**Affiliations:** 1 State Key Laboratory of Pharmaceutical Biotechnology, Institute for Brain Sciences, Chinese Academy of Medical Sciences Research Unit of Extracellular RNA, Jiangsu Engineering Research Center for MicroRNA Biology and Biotechnology, Advanced Institute for Life Sciences, Chemistry and Biomedicine Innovation Center, School of Life Sciences, Nanjing University, Nanjing, Jiangsu, China; 2 School of Electronic Science and Engineering, Nanjing University, Nanjing, Jiangsu, China; 3 Peng Cheng Laboratory, Shenzhen, China; 4 Department of Chemistry and the Nebraska Center for Integrated Biomolecular Communication (NCIBC), University of Nebraska-Lincoln, Lincoln, Nebraska, USA; 5 Department of Neuroscience and Friedman Brain Institute, Icahn School of Medicine at Mount Sinai, New York, New York, USA

**Keywords:** computer modeling, molecular docking, G protein-coupled receptor, insect, peptide conformation, signal transduction, ligand–receptor interaction, leucokinin, Autodock Vina, *Aplysia*, AI, artificial intelligence, ALK, *Aplysia* leucokinin-like peptide, ALKR, *Aplysia* leucokinin-like peptide receptor, CCK, cholecystokinin, cDNA, complementary DNA, CHO-K1 cells, Chinese hamster ovary K1 cells, FBS, fetal bovine serum, GPCRs, G protein-coupled receptors, IP1, inositol monophosphate, LK, leucokinin, LKR, leucokinin receptor, TMs, transmembrane domains

## Abstract

The protostome leucokinin (LK) signaling system, including LK peptides and their G protein-coupled receptors, has been characterized in several species. Despite the progress, molecular mechanisms governing LK peptide–receptor interactions remain to be elucidated. Previously, we identified a precursor protein for *Aplysia* leucokinin-like peptides (ALKs) that contains the greatest number of amidated peptides among LK precursors in all species identified so far. Here, we identified the first ALK receptor from *Aplysia*, ALKR. We used cell-based IP1 activation assays to demonstrate that two ALK peptides with the most copies, ALK1 and ALK2, activated ALKR with high potencies. Other endogenous ALK-derived peptides bearing the FXXWX-amide motif also activated ALKR to various degrees. Our examination of cross-species activity of ALKs with the *Anopheles* LK receptor was consistent with a critical role for the FXXWX-amide motif in receptor activity. Furthermore, we showed, through alanine substitution of ALK1, the highly conserved phenylalanine (F), tryptophan (W), and C-terminal amidation were each essential for receptor activation. Finally, we used an artificial intelligence–based protein structure prediction server (Robetta) and Autodock Vina to predict the ligand-bound conformation of ALKR. Our model predicted several interactions (*i.e.*, hydrophobic interactions, hydrogen bonds, and amide-pi stacking) between ALK peptides and ALKR, and several of our substitution and mutagenesis experiments were consistent with the predicted model. In conclusion, our results provide important information defining possible interactions between ALK peptides and their receptors. The workflow utilized here may be useful for studying other ligand–receptor interactions for a neuropeptide signaling system, particularly in protostomes.

Neuropeptides are the most diverse class of neuromodulators in both protostomes and deuterostomes (1, 2, 3, 4, 5). Neuropeptides primarily act on G protein-coupled receptors (GPCRs) to influence a variety of behaviors and physiological processes, including feeding, locomotion, and reproduction. Although a growing number of neuropeptides and their receptors have been characterized (6, 7, 8, 9, 10, 11), molecular-level details of how peptide ligands engage their receptors remain poorly understood in many cases. Ideally, this information is obtained through high-resolution structures of a GPCR in a bound state with its peptide ligand (*e.g.*, by X-ray crystallography, NMR, or cryogenic-EM) (12, 13, 14, 15). However, obtaining high-resolution structures remains a daunting task for most GPCRs, particularly for neuropeptide signaling systems that are only present in protostomes (most invertebrates) that lack well-studied homologs (12, 13, 15). Thus, previous work has often used amino acid substitution and other experiments to characterize the roles of specific residues in a ligand that may be critical for receptor activity (10). Efforts are also made to infer receptor activity of ligands based on ligands’ structure in solution (16, 17, 18, 19, 20, 21, 22, 23, 24), although these analyses generally do not allow one to draw conclusions about specific interactions between ligands and the receptor. Moreover, previous work has analyzed the ligand–receptor interactions based on homology modeling of the structures of protostome receptors with their vertebrate homologs (*e.g.*, insect receptors for cholecystokinin (CCK) (25) and Neuromedin U (26)). However, few have explored the contributions of specific residues or other properties of ligands to receptor activity based on the structure of a protostome’s receptor that has no known homologs in deuterostomes (vertebrates and some invertebrates), partly because a protein structure cannot be obtained using a homology modeling approach. In the present work, we utilize a molluscan model system, *Aplysia californica* (11, 27, 28, 29, 30, 31, 32, 33, 34, 35, 36, 37, 38, 39, 40, 41, 42, 43, 44, 45, 46, 47, 48, 49, 50, 51, 52, 53, 54, 55, 56, 57, 58, 59), to study this issue using *Aplysia* leucokinin peptides (ALKs) (60) and their receptor. In particular, recent successful efforts (14) have been made to predict protein structure based on the amino acid sequence of a protein, particularly template-free modeling (61) using artificial intelligence (AI) deep machine learning algorithms such as Robetta (62) and AlphaFold (63). Presumably, the AI prediction approach could be applied to protostome proteins that have no homologs in deuterostomes. Thus, we sought to demonstrate this applicability using the ALK signaling system by taking advantage of the Robetta server, which is freely available, to obtain a predicted receptor structure. We then used Autodock Vina (64, 65) to predict the bound conformations of the ligands (including their analogs) with the receptor.

The leucokinin (LK) signaling system is known to be present only in protostomes (6, 7, 66, 67). LK peptides were first identified in cockroach *Leucophaea maderae* (now named *Rhyparobia maderae*) through bioassays on hindgut contractions (68, 69, 70, 71, 72). Subsequently, a number of LK peptides and some of their precursor proteins have been identified in arthropods, tardigrades, annelids, and molluscs. The C-termini of LK peptides share a FXXWX-amide motif (Fig. 1). LKs play diverse roles in the regulation of ion and water homeostasis, feeding, sleep–metabolism interactions, state-dependent memory formation, as well as modulation of gustatory sensitivity and nociception (66, 67). Interestingly, the first LK receptor (LKR) was found in the mollusc *Lymnaea* (73) and subsequently in several insect species (74, 75, 76, 77, 78, 79, 80). For all LKRs thus far identified, there is only a single receptor in each species. Prior studies have investigated activity of ligands on the LKR in each species, but no study has characterized the mechanisms of the ligand–receptor interactions at molecular resolution.

**Figure 1:**
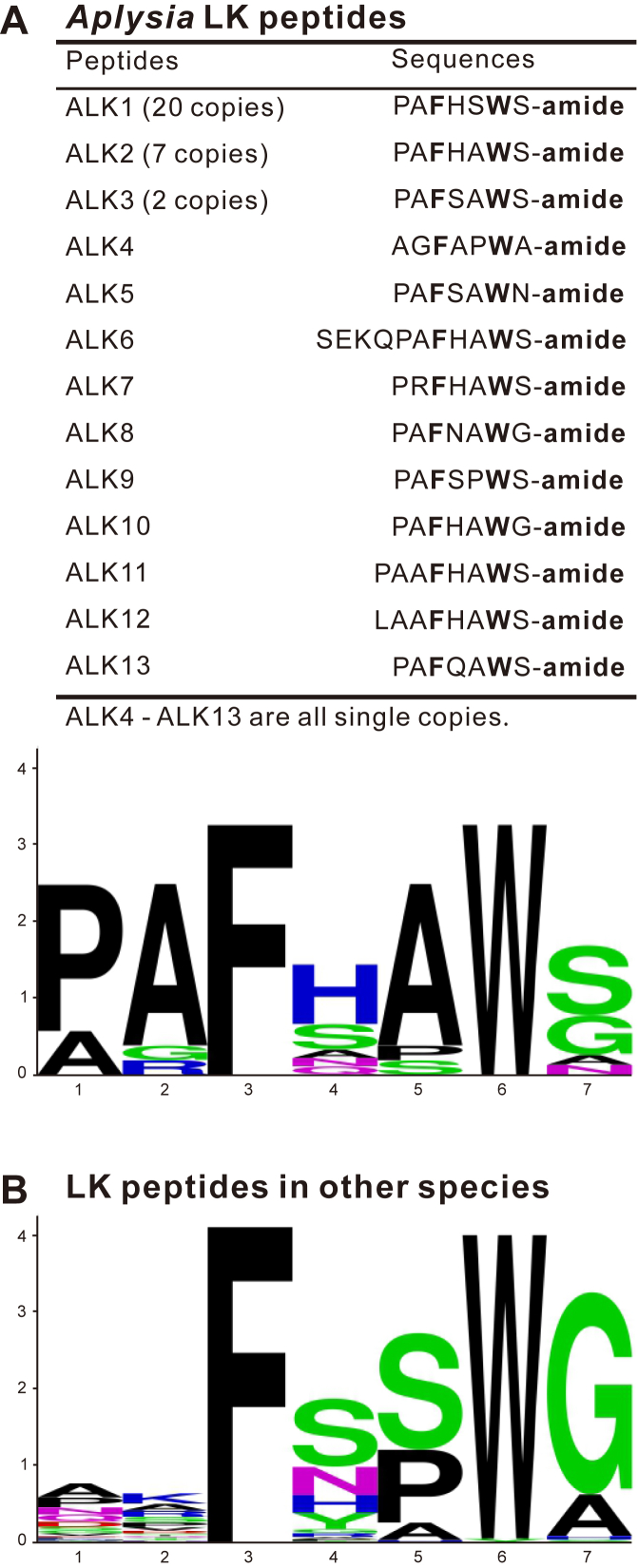
**Comparison of ALK peptides and LK peptides in other species.***A*, primary sequences and sequence logo plot for the C-terminus of ALK peptides relevant to this study. All ALK peptides have a conserved FXXWX-amide motif. The shared residues and amidation are shown in *bold*. Note that the consensus sequence is identical to ALK2. *B*, sequence logo plot of LK peptide C-terminal conserved sequences in other species (excluding *Aplysia*) (see Table S4 for information on the selected sequences). The sequence logo plots in (*A*) and (*B*) show that shared residues in ALKs and LKs are similar. ALK, *Aplysia* leucokinin-like peptide; LK, leucokinin.

We have previously identified an *Aplysia* leucokinin (ALK) precursor protein that encodes up to 40 putative ALK peptides (60), representing one of the longest neuropeptide precursors known. The diverse ALK peptides generated from this precursor share the FXXWX-amide motif present in LKs in other invertebrates (Fig. 1). Here, we describe a newly identified receptor for ALK peptides, termed ALK receptor (ALKR). We took advantage of the diverse ALK ligands and showed that all the native ALKs could activate the receptor, albeit with different potencies. Mutagenesis studies of the ligands and receptor demonstrated that the conserved amino acids and amidation in different LKs are critical for receptor activity and gave insight into the roles of specific receptor residues critical for ligand-induced activation. Together with an AI-predicted model of peptide–receptor complex, these computational and experimental analyses elucidated the specific roles of several residues in both the peptide and receptor in this interaction. Overall, the results provide detailed information on the ALK–ALKR interaction and support the effectiveness of AI prediction of structures of protostome proteins.

## Results

### Identification of a putative ALKR

**Figure 2:**
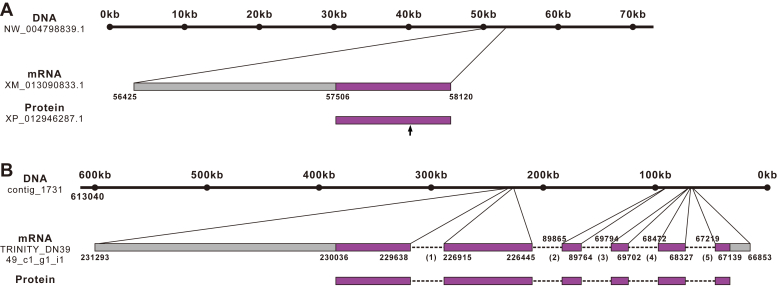
**Gene expression mapping of ALKR.***A*, BLASTn search result of Genbank showing a DNA that encodes a partial sequence of the ALKR. There is no intron in the DNA, but the *arrow* indicates where a possible intron is located as shown in (*B*). *B*, BLASTn search result of *Aplysia* transcriptome databases and genome databases showing a DNA that encodes a complete sequence of ALKR. The DNA sequence was a complementary sequence of the genome, so the nucleotide number on *top* starts from the *right*. Exons were drawn to scale but introns were not. The length of intron 1: 2723 bp; intron 2: 136,580 bp; intron 3: 19,970 bp; intron 4: 1230 bp; intron 5: 1108 bp. Approximate locations of the exons and introns on the genome are marked. ALK, *Aplysia* leucokinin-like peptide; ALKR, *Aplysia* leucokinin-like peptide receptor.

The ALK precursor protein has been previously characterized (60). Here, we sought to identify a receptor in *Aplysia* for ALK peptides. We used the receptor for lymnokinin, which is the mollusc *Lymnaea stagnalis* homolog of LK peptides (NCBI accession: U84499.1), as a query to perform a BLASTn search of NCBI GeneBank, and found a sequence (XM_013090833.1). The protein it encodes contains 205 amino acids (XP_012946287.1) but appears to be incomplete (see below). It is located on the *Aplysia* genomic sequence NW_004798839.1, which has 72,762 bp (Fig. 2*A*).

We then performed BLASTn search of *Aplysia* transcriptome databases (http://aplysiatools.org:4567/) and genome databases (http://aplysiatools.org:8080/) using the *Lymnaea* lymnokinin receptor. This search returned an mRNA with an ORF of 1290 bp encoding 429 amino acids in the transcriptome database. In *Aplysia* genome database, this mRNA was located in contig_1731 and consisted of six exons and five introns (Fig. 2*B*). There is a long intron between the second and third exons, about 136 kb. To verify whether the sequence was predicted to be a complete GPCR, we analyzed the sequence using NCBI-conserved domain database (81) (https://www.ncbi.nlm.nih.gov/Structure/cdd/wrpsb.cgi) and TMHMM 2.0 (82, 83) (https://services.healthtech.dtu.dk/service.php?TMHMM-2.0). The analysis showed that the identified protein sequence was indeed predicted to be a GPCR with seven transmembrane domains (TMs) (Fig. S1). In addition, the putative receptor also contains the conserved Asp-Arg-Tyr (DRY) motif (84), located in the second intracellular loop, and the conserved Asn-Pro-Xaa-Xaa-Tyr (NPXXY) motif (85) located in the seventh TM helix, suggesting that it is a Class A GPCR (86) (Fig. S2). This sequence had 76.9% similarity with the *Lymnaea* lymnokinin receptor (Table S1), therefore, we tentatively named it an ALKR. Note that the 205-residue protein sequence from NCBI (protein: XP_012946287.1) is identical to the first 205 residues from this putative ALKR, indicating the receptor sequence deposited on NCBI is only a partial sequence. We also analyzed sites for possible posttranslational modifications in the ALKR (Supporting Results and Discussion and Table S2).

**Figure 3:**
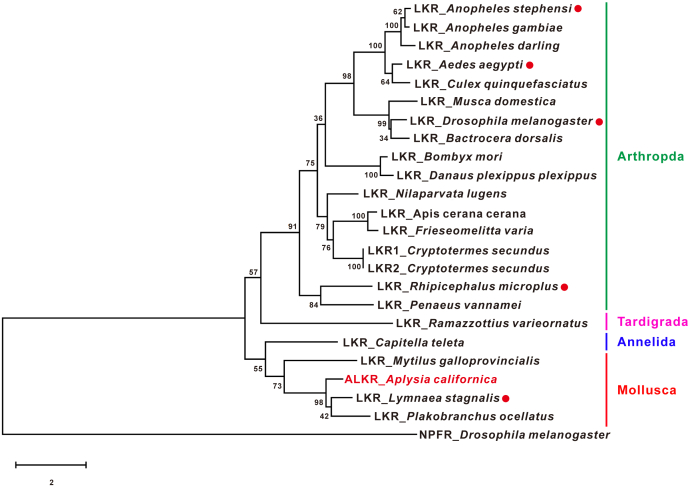
**A phylogenetic tree of leucokinin receptors in protostomes.***Drosophila* neuropeptide F receptor (NPFR) was used as an out-group to root the tree. Amino acid sequences of full-length receptors were used for the analysis (see Table S1 for information on the selected sequences). Sequences were aligned using the Clustal W. Maximum-likelihood trees were constructed by MEGA X software using JTT+G method. The tree is drawn to scale, with branch lengths measured in the number of substitutions per site. The numbers at the nodes of the branches represent the percentage bootstrap support (1000 replications) for each branch. LKRs that have been functionally characterized are indicated by a *red dot* after the species name. LKR, leucokinin receptor.

We generated a phylogenetic tree of LKRs from selected species in *Arthropoda*, *Mollusca*, *Annelida*, and *Tardigrade* (Fig. 3). Among the selected species, the LKRs of *Lymnea* *stagnalis*, *Drosophila melanogaster*, *Aedes aegypti*, *Anopheles stephensi*, and *Rhipicephalus microplus* have been functionally characterized (73, 74, 75, 76, 77, 78, 79, 80) (Table S1). We performed BLASTp search of NCBI databases using the above verified sequences from the five species and found several additional sequences that are not annotated as putative LKRs, but we putatively named them LKR (Table S1). Indeed, when we used these putative LKRs to perform BLASTp search of NCBI databases, the most similar sequence is actually one of the above five sequences functionally characterized. Currently, there is only one type of LKR in the vast majority of known species, and no other subtypes exist (see (67)) with the possible exception of *C. secundus* (see Discussion). The phylogenetic tree showed that the ALKR is closely related to LKRs from other molluscs, including *Lymnaea* (*i.e.*, lymnokinin receptor: 76.9%) and *Plakobranchus ocellatus* (Similarity: 72.6%).

Finally, we sought to clone the ALKR from the *Aplysia* complementary DNA (cDNA). We designed primers (Table S3) using the putative ALKR sequence and obtained a PCR product (Fig. S3) that was identical with the putative ALKR in transcriptome database.

### Activation of the putative ALKR by ALK peptides

As shown previously (60), the ALK precursor is predicted by NeuroPred (http://stagbeetle.animal.uiuc.edu/cgi-bin/neuropred.py) (87) to code 40 unique putative neuropeptides, with a total of 66 copies including duplicate sequences. These 40 peptides were also detected using Matrix-Assisted Laser Desorption Ionization mass spectrometry (Fig. S4, see also Table S2 of (60)). Among them, 13 peptide sequences have the conserved FXXWX-amide motif at the C-terminus, and are predicted to be fully processed ALKs. The two peptides with the largest number of copies were named ALK1 (20 copies) and ALK2 (seven copies). Except ALK1 and ALK2, we named the neuropeptide with two copies (P505-S511) ALK3, and the other ten peptides were named ALK4-ALK13 according to their appearance order on the precursor (Fig. 1*A*). Among all ALKs, ALK11 and ALK12 are octapeptides, ALK6 is an undecapeptide, and the others are heptapeptides. A sequence logo plot of the peptides (Fig. 1*A*, bottom panel) showed that aromatic phenylalanine and tryptophan residues are completely conserved in ALK sequences. C-terminal amidation is also conserved in all ALK peptides. These conserved elements are also found in LKs from other species (Fig. 1*B* and Table S4). Notably, the amino acid sequence with the highest frequency at each site of all ALKs was the same as ALK2 sequence. We also generated a sequence logo plot for most of the known LKs from different species other than ALKs (Fig. 1*B* and Table S4). Interestingly, the two frequency plots indicate that the amino acids that are most frequent at most positions are similar for the ALKs and LKs from other species. Only at the second position was there some minor difference (AGR for ALKs *versus* KAR for other species). This suggests that ALKs may be a good representation of LKs in different species.

**Figure 4:**
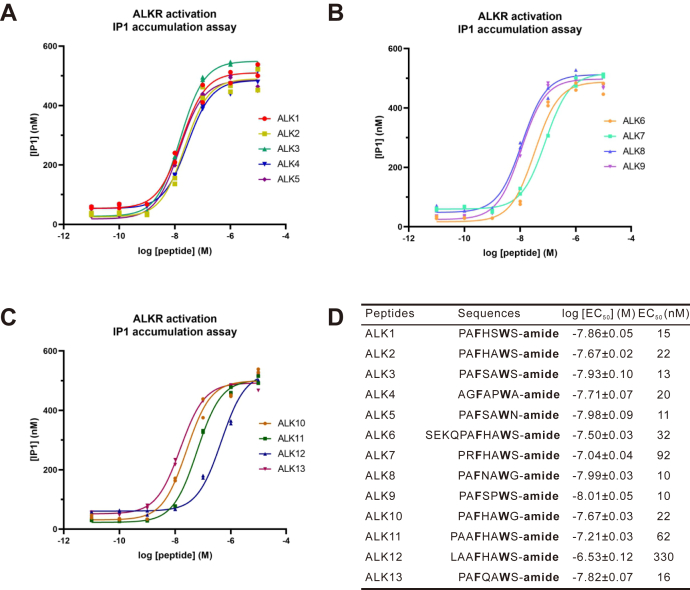
**Activation of the ALKR by native ALK peptides.***A*–*C*, dose-response curves for ALK peptides on CHO-K1 cells transfected with ALKR, as determined by IP1 accumulation assay. Each point represents the value from an individual well on the plate, with each condition run in duplicate. *D*, sequences of all peptides tested and a summary of the average log [EC_50_] and EC_50_ on ALKR. The log [EC_50_] values are reported as the mean ± SEM. For ALK1, *n* = 13; for ALK2, *n* = 4; for other native peptides, *n* = 3. ALK, *Aplysia* leucokinin-like peptide; ALKR, *Aplysia* leucokinin-like peptide receptor; IP1, inositol monophosphate.

To examine whether the native ALKs could activate the putative ALKR, we expressed the ALKR in Chinese hamster ovary K1 (CHO-K1) cells and examined ALK-mediated changes in the concentration of inositol monophosphate (IP1), a degradation product of the second messenger (inositol trisphosphate) in the G_q_ signaling pathway upon ligand-induced activation (88) (Figs. 4 and S5). In these experiments, we did not need to cotransfect with a promiscuous Gα_q_ protein (see (9, 10, 11, 89)) to elicit IP1 accumulation upon ALK peptide stimulation, suggesting that ALKR can associate with native Gα_q_ proteins in CHO-K1 cells. All endogenous ALK peptides could activate ALKR in a dose-dependent manner (Fig. 4, *A*–*C*). Nine of the 13 endogenous ALKs exhibited high potency, with EC_50_ values ranging from 10 nM to 22 nM. The lowest EC_50_ values were for ALK8 (EC_50_ = 10 nM) and ALK9 (EC_50_ = 10 nM). The EC_50_ values for ALK1, ALK2, ALK3, ALK4, ALK5, ALK10, ALK13 were slightly higher than that of ALK9, but there was no statistically significant difference between them (Table S5). ALK6, ALK11, and ALK7 had a somewhat lower potency, with EC_50_ value of 32 nM, 62 nM, and 92 nM, respectively. ALK12 (EC_50_ = 330 nM) had the lowest activity and was significantly different than all other ALKs (Table S5).

To determine the selectivity of ALKs on the ALKR, we tested the effects of ALK1 on a different *Aplysia* receptor (9, 59), that is, the receptor for *Aplysia* allatotropin-like peptide (45). ALK1 did not show any activation of *Aplysia* allatotropin-like peptide receptor (Fig. S6*A*, *n* = 3). Conversely, *Aplysia* allatotropin-like peptide also did not have any effect on ALKR (Fig. S6*B*, *n* = 3).

### Cross-activity with an insect (*A. stephensi*) receptor

**Figure 5:**
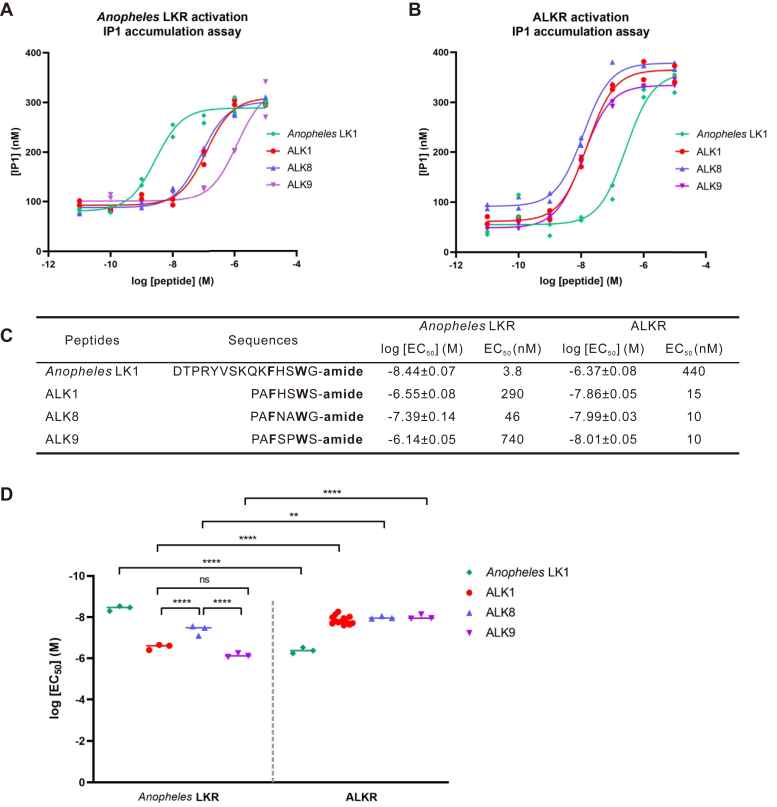
**Activation of *Anopheles* LKR and ALKR by *Anopheles* LK1 and three ALKs.***A* and *B*, dose-response curves showing the ability of *Anopheles* LK1, ALK1, ALK8, and ALK9 to activate *Anopheles* LKR (*A*) or ALKR (*B*) transfected in CHO-K1 cells, as determined by IP1 accumulation assay. Each point represents the value from an individual well on the plate, with each condition run in duplicate. *C*, sequences of all peptides tested and summary of the average log [EC_50_] and EC_50_ on ALKR. For active peptides, log [EC_50_] values are reported as the mean ± SEM from at least three independent experiments. For ALK1 acting on ALKR, *n* = 13; in other cases, *n* = 3. *D*, for peptides shown in (*C*), log [EC_50_] values showed overall significant difference (*F* (7, 26) = 88.06, *p* < 0.0001). Bonferroni post-hoc test: ∗∗*p* < 0.01; ∗∗∗∗*p* < 0.0001. ALK, *Aplysia* leucokinin-like peptide; ALKR, *Aplysia* leucokinin-like peptide receptor; IP1, inositol monophosphate; LK, leucokinin; LKR, leucokinin receptor.

Considering the similarity of leucokinin-like peptides in different species (Fig. 1), we sought to evaluate cross-activity between leucokinin peptides and their receptors in different species. We selected *A. stephensi* because it is a pest, and this would also provide an opportunity to determine if *Aplysia* LKs could be potentially used as insecticides (67). Specifically, we examined leucokinin 1 (LK1, the most active LK in *Anopheles*) and the LKR of *A. stephensi* for experiments with ALKR and the three most potent peptides (ALK1, ALK8, ALK9). In preliminary experiments, we found that the *Anopheles* receptor was not responsive to any of the LKs when there was no cotransfection with a promiscuous Gα_q_ protein but was responsive when there was cotransfection with promiscuous Gα_q_. Thus, we cotransfected promiscuous Gα_q_ with the *Anopheles* receptor, whereas there was no cotransfection of promiscuous Gα_q_ for ALKR. The results (Fig. 5) showed that both *Aplysia* and *A. stephensi* LKRs displayed low EC_50_ values of 3.8 to 15 nM when activated by their own endogenous peptides. The EC_50_ values of the cross-species neuropeptides and receptor pairs were significantly higher (ranging from 46 to 740 nM).

### The roles of specific residues and amidation of the ALKs to receptor activation based on exploration of ligand–receptor interactions

**Figure 6:**
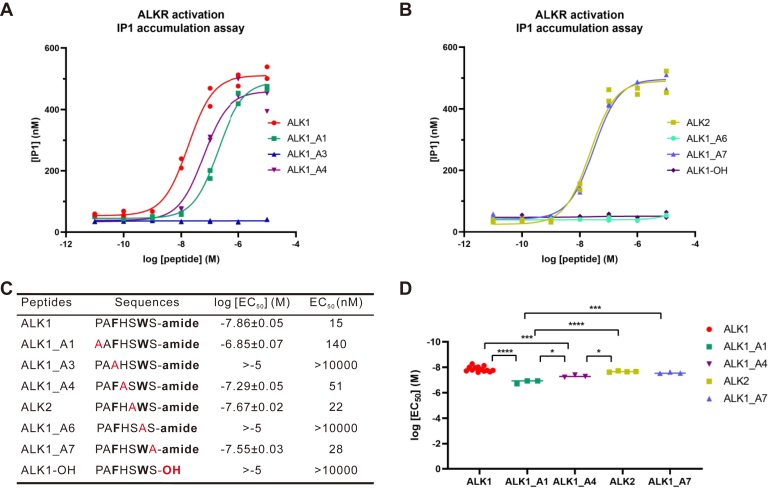
**Activation of ALKR by ALK1 and its analogs generated by alanine substitution or by removing amidated C-terminus.***A* and *B*, dose-response curves for ALK1 analogs on CHO-K1 cells transfected with ALKR, as determined by IP1 accumulation assay. Each point represents the value from an individual well on the plate, with each condition run in duplicate. *C*, sequences of all peptides tested and a summary of the average log [EC_50_] and EC_50_ on ALKR, as determined by IP1 accumulation assay. Alanine substitution or no C-terminal amidation are shown in *red*. For active peptides, log [EC_50_] values are reported as the mean ± SEM. For inactive peptides, the log [EC_50_] is listed as being greater than the highest concentration tested. For ALK1, *n* = 13; for ALK2, *n* = 4; for other ALK1 analogs, *n* = 3. *D*, for peptides shown in (*C*), log [EC_50_] showed overall significant difference (*F* (4, 21) = 27.49, *p* < 0.0001). Bonferroni post-hoc test: ∗*p* < 0.05; ∗∗∗*p* < 0.001; ∗∗∗∗*p* < 0.0001. ALK, *Aplysia* leucokinin-like peptide; ALKR, *Aplysia* leucokinin-like peptide receptor; IP1, inositol monophosphate.

To determine the influence of C-terminal amidation and each residue on activity of ALKs, we synthesized ALK analogs with each residue of ALK1 substituted by Ala (Fig. 6). The dose-response curves showed that the ALKR was not activated by ALK analogs when F3 or W6 (Fig. 1) were replaced by A or the C-terminal amidation was replaced with a carboxylic acid. In contrast, ALKR could be effectively activated by the other analogs. However, potency was significantly reduced when P1 or H4 of ALK1 was replaced with A (Fig. 6*C*). P1 substitution had the largest influence, and the EC_50_ value was 140 nM after it was replaced (ALK1_A1). H4 substitution had a smaller effect on ALK activity, and EC_50_ value was 51 nM after this residue was replaced (Fig. 6, *C* and *D*).

**Figure 7:**
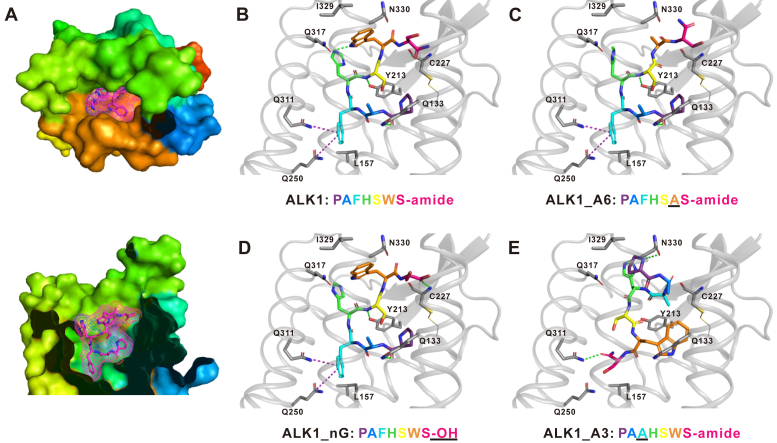
**Docking results of ALK1 and ALK1 analogs with ALKR.***A*, global diagrams of docking of ALK1 with ALKR. The ALKR is colored coded with N-terminus shown in *dark blue*, the C-terminus shown in *red*. The ligand (ALK1) is shown in *purple*. *Upper panel*, top view after removing residues on ALKR N-terminus; *lower panel*, side view after removing residues in ALKR that are in front of the ALK1. *B*–*E*, binding regions of ALK1 (*B*), ALK1_A6 (*C*), ALK1-OH (*D*), ALK1_A3 (*E*) with ALKR. ALK1 is active, whereas the other three analogs are not active on the ALKR. For the three inactive analogs, the residue or the C-terminus that is different from ALK1 is *underlined*. ALK1, ALK1_A6, and ALK1-OH have a similar bound conformation, while ALK1_A3 had a different bound conformation. H-bonds are shown in *dotted green lines* and amide-pi interactions are shown in *dotted purple lines*. For the residue C227, the entire amino acid is shown because the N atom on its backbone formed an H-bond with ALK1-OH (*D*). For the other residues highlighted in the ALKR, only side chains are displayed. ALK, *Aplysia* leucokinin-like peptide; ALKR, *Aplysia* leucokinin-like peptide receptor.

To examine the ligands’ interaction with the receptor and possibly explain the activity of these ALKs and their analogs, we used Robetta protein prediction server (https://robetta.bakerlab.org/) (62) to generate a receptor structure (Figs. 7*A* and S7, *A* and *B*) and evaluated that the predicted model was appropriate and reliable based on the Ramachandran plot (90) and QMEAN score (91) (Supporting Results and Discussion and Figs. S8–S11). This model had conserved bound conformations in extracellular loops 1 and 2 among peptide GPCRs, with extracellular loop 2 forming a β hairpin (15) (Fig. S7*C*). This structure was then imported into Autodock Vina (65) to generate docking results with ALK1 and its analogs (Fig. 7, *B*–*E*, Table S6, and Fig. S7). The molecular model predicted that ALK1, ALK2, and all of the active ALK analogs from the alanine scan bound to the ALKR putative-binding pocket in a similar conformation (Fig. 7*B*). In this conformation, L157 and I329 of the receptor formed two hydrophobic interactions with the peptide analogs (Table S6). Y213, Q317, and Q133 in ALKR formed three hydrogen bonds (H-bonds) with each of these effective analogs (Table S6). Q311 and Q250 in the ALKR formed amide-pi stacking interactions (for bound distances and angles, Table S6) (92, 93) with the effective analogs. Interestingly, the docking model also predicted that inactive analogs ALK1_A6 (W6 replaced by A) (Fig. 7*C*) and ALK1-OH (no C-terminal amidation) (Fig. 7*D*) bound to the receptor in the similar conformation as the active analogs, although some interactions differed (Supporting Results and Discussion). In contrast, ALK1_A3 (F3 replaced by A) did not bind to ALKR in the common conformation (Fig. 7*E*). Comparison of the ALKR and 12 LKRs in other species (Fig. S12) suggest that Q133, Q250, Q311, and I329 are completely conserved, while L157 and Q317 are moderately conserved (Supporting Results and Discussion).

### Mutagenesis of the ALKR

**Figure 8:**
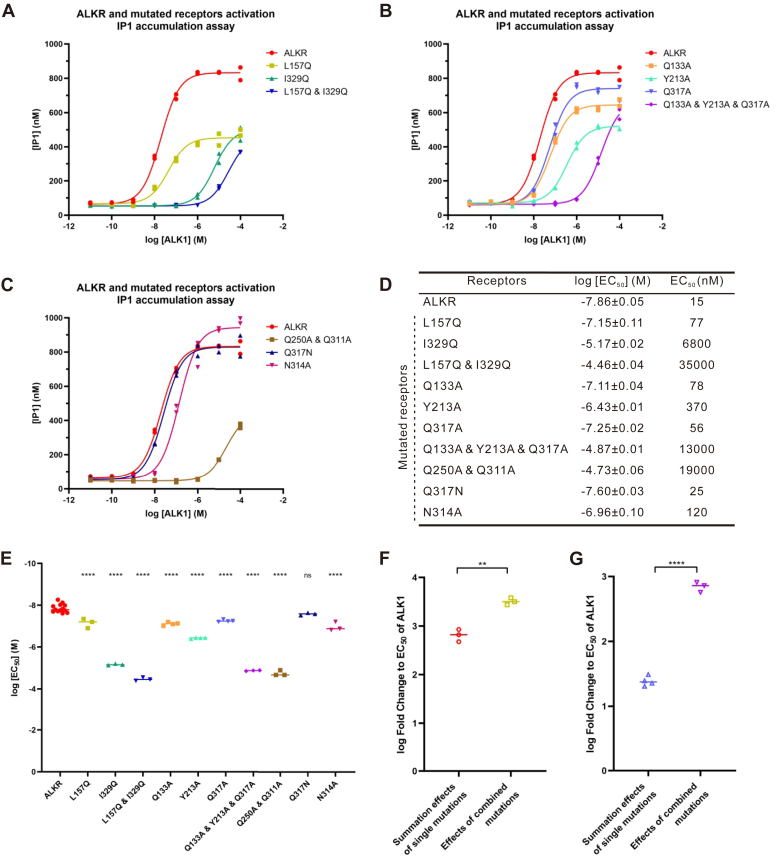
**Mutagenesis of specific residues in the ALKR.** These residues are predicted to be involved in hydrophobic interactions (L157 and I329), H-bonds (Q133, Y213, and Q317) or amide-pi stacking (Q250 and Q311), or are suspected to interact with the ligand (N314). *A*–*C*, dose-response curves showing the ability of ALK1 to activate the ALKR and the ALKR mutants expressed in CHO-K1 cells, as determined by IP1 accumulation assay. *D*, summary of the results shown in (*A–C*): ALKR (*n* = 13), L157Q (*n* = 3), I329Q (*n* = 3), L157Q & I329Q (*n* = 3), Q133A (*n* = 4), Y213A (*n* = 4), Q317A (*n* = 4), Q317N (*n* = 4), Q133A & Y213A & Q317A (*n* = 3), Q250A & Q311A (*n* = 3), and N314A (*n* = 3). *E*, group data comparing the effects of mutations (*F* (10, 35) = 307.5, *p* < 0.0001). Bonferroni post-hoc test: ns, not significant; ∗∗∗∗*p* < 0.0001 (All comparisons were made against ALKR). *F*, fold change of L157Q & I329Q combined effects was compared with that of summation effects of L157/I329 single-site mutations using *t* test: ∗∗*p* < 0.01. *G*, fold change of Q133A & Y213A & Q317A combined effects was compared with that of summation effect of Q133/Y213/Q317 single-site mutations using *t* test: ∗∗∗∗*p* < 0.0001. ALK, *Aplysia* leucokinin-like peptide; ALKR, *Aplysia* leucokinin-like peptide receptor; IP1, inositol monophosphate.

We next sought to determine whether the ALKR residues that are predicted to make contact with the peptide ligands in our model (L157, I329, Q133, Y213, Q317, Q250, Q311) are critical for receptor activation. To gain insight into the effect of each of these residues on receptor function, we generated point mutants using site-directed mutagenesis (Fig. 8). When L157 and I329, which were each predicted to form hydrophobic interactions with the peptide ligands, were individually mutated to the polar Q, the EC_50_ values for ALK1 activation were significantly increased (77 nM and 6800 nM, respectively). When both of these two residues were simultaneously mutated to Q, the experimental EC_50_ value (35,000 nM) became much larger and significantly different from those of the single-site mutant receptors (Fig. 8*A*). The summation effect from the individual mutations was much lower than the combined effect of the simultaneous mutations, indicating a synergistic action between two hydrophobic interactions (Fig. 8*F*). For Q133, Y213, and Q317, which were predicted to form H-bonds with the peptide ligands, we found that EC_50_ from ALK1 was significantly increased to 78 nM, 370 nM, and 56 nM, respectively, when each of these residues were mutated to A. When all three residues were simultaneously mutated to A, the experimental EC_50_ (13,000 nM) became much larger and significantly different from those of the single-site mutated receptors (Fig. 8*B*). In the same fashion, the summation effect from the individual mutations were much lower than the combined effect of the simultaneous mutations indicating a synergistic action between three H-bonds (Fig. 8*G*). Note that when Q317 was mutated to N, which has similar properties as Q, the experimental EC_50_ of the mutated receptor was not significantly different from the ALKR (Fig. 8*C*). For residues Q250 and Q311, which were predicted to be involved in amide-pi stacking interactions, simultaneous mutation of both of these residues to A significantly increased the EC_50_ value (19,000 nM) (Fig. 8*C*). These results indicate that the above residues play some roles in ligand activation of ALKR, and that the molecular docking results may be useful in predicting details of ligand–receptor interactions.

In addition to the residues predicted to directly interact with ALKs in the docking results, we also mutated the residue N314 to A (Fig. 8*C*). N314 corresponds to a relatively conserved residue (55% of N or Q) that might interact with ligands in other receptors for C-terminal amidated peptides (13) (see Table S7). In our model, N314 is within 3.3 to 3.4 Å of H4 in the effective peptide ligands, suggesting that these residues have the potential to interact (*e.g.*, *via* H-bonds). Consistent with this prediction, mutation of N314 to A significantly increased EC_50_ (120 nM), suggesting that N314 plays a role in receptor activity for ALKR (Supporting Results and Discussion).

To determine if mutagenesis could affect the expression of the receptors on the surface of CHO-K1 cells, we added FLAG tags to the ALKR and the mutant receptors (Figs. S13 and S14) to facilitate cell surface expression analysis. EC_50_ values for receptors with the FLAG tags (Fig. S13) showed a similar pattern of changes for mutant receptors without the FLAG tags (Fig. S13, *D*–*F*). In addition, cell surface receptor expression experiments showed that expression of the mutant receptors did not change significantly from that of the ALKR (Fig. S14), suggesting that changes in EC_50_ of mutant receptors (Figs. 8 and S13) compared to the ALKR were not due to changes in receptor expression.

## Discussion

We have previously characterized an LK precursor protein in *Aplysia* and showed that LK signaling plays a significant role in modulating the feeding circuit (60). In this work, we have identified the ALKR in *Aplysia* for the first time and through bioinformatics, cell-based assays, computer modeling, and mutagenesis, demonstrated how diverse ALK ligands or their analogs may interact with the receptor.

### Actions of ALKs on ALKR and cross-activity with Anopheles LKR

Although LK peptides were first identified in insects, previous work has shown that the LK signaling system is also important in molluscs, such as playing a major role in modulating feeding circuit in *Aplysia* (60). Interestingly, the first LKR was found in pond snail *L. stagnalis* (73). The LK precursor in *Aplysia*, perhaps a longest neuropeptide precursor that is known, generates a number of ligands (60) with shared motifs that are representative of LKs in other species, including arthropods (Fig. 1). Our present identification of ALKR demonstrates multiple notable features in the receptor. First, there is a long intron of about 136 kb between the second and third exons, which is uncommon in other species. In fact, the incomplete sequence currently deposited in NCBI only contains the first exon and some partial sequence in the second exon (Fig. 2). Perhaps, the long intron after the second exon might be partly the reason why the ALKR sequence in NCBI is incomplete. Second, there are a number of sites for various potential posttranslational modifications (94) (See Supporting Results and Discussion). Finally, it should be noted that only a single LKR sequence (no subtypes) appears to exist in the vast majority of known species, including all six verified LKRs (Fig. 3) (67). (Note that *C. secundus* might have two sequences, LKR1 and LKR2, which are nearly identical except that LKR2 has 19 fewer amino acids at the N-terminus than LKR1).

Receptor activity studies using cell-based assays with recombinant receptor demonstrated that ALKR is indeed the receptor for ALK peptides. Notably, all 13 native ALK peptide ligands are able to activate the receptor with various potencies, with EC_50_ values ranging from 10 to 330 nM (Figs. 4 and S4). Because all of the native ALK peptides share the F3, W6 (using ALK1 numbering), and C-terminal amidation, these results suggested that variations in other amino acids can moderately affect ALK activity.

The cross-species activity between the LK peptides and receptors of *Aplysia* and *A. stephensi* (77) also provides information on the roles of various residues. LKs do activate each other’s receptor, with EC_50_ ranging from 46 to 740 nM (Fig. 5), consistent with the idea that the shared residues and C-terminal amidation among LKs are important for receptor activity. However, the EC_50_ values for cross-species activity are significantly higher than the EC_50_ values for LKs activating native receptors, indicating that other residues in LK peptides also play roles in receptor potency. Interestingly, although ALK1, ALK8, ALK9 had similar EC_50_ values with the ALKR, their EC_50_ values with *Anopheles* LKR are significantly different, with ALK8 having the lowest EC_50_ value. This result further supports the observation that the residues other than F and W play roles in receptor activity. Given that LKs have been considered for pest control (67) and ALK8 had the lowest EC_50_ on *Anopheles* LKR and is shorter than *Anopheles* leucokinin 1 (7 *versus* 15 amino acid), ALK8 could potentially act as an economical insecticide. Finally, a notable difference between these receptors is that the *Aplysia* receptor does not need cotransfection of a promiscuous Gα_q_ protein to couple to phospholipase C in CHO-K1 cells, while the *Anopheles* receptor does. This suggests that although they are orthologous, the molluscan and insect receptors might differ in their association with G proteins and intracellular signaling pathways.

### Key residues involved in ligand–receptor interactions

The above analysis and previous work on the five receptors in insects and *Lymnaea* (73, 74, 75, 76, 77, 78, 79, 80) provided some basis for understanding the roles of each residue and the amidation on the receptor activity, but further work is needed to clarify their roles. Here, we first evaluated what elements of the ALK1 peptide sequence is critical for receptor activity by removing amidation or substituting each residue with alanine. The findings provided direct evidence that amidation and highly conserved residues (F and W) are indeed essential for receptor activity, whereas the other residues play a lesser role (Fig. 6).

Importantly, we have taken a further step in studying ligand–receptor interactions using computer modeling and mutagenesis of the receptor. We used the protein structure prediction server Robetta (62) to generate an ALKR structure. Subsequently, we used Autodock Vina (65) to predict the bound conformations, H-bonds, hydrophobic interactions, and amide-pi stacking interactions between ALK1 or ALK1 analogs with ALKR (Fig. 7, and Supporting Results and Discussion). Specifically, two amino acid residue side chains in ALKR are predicted to directly engage in hydrophobic interactions with the ligand: L157 and I329. Three amino acid residues in ALKR are predicted to form hydrogen bonds with ALK peptides: Q133, Y213, and Q317. Finally, two amino acid residues from ALKR form amide-pi stacking interactions (92, 93) with the ligands: Q250 and Q311. Moreover, comparison with other LKRs showed that several of the above important residues, including Q317, are largely conserved (Fig. S12), suggesting that similar ligand–receptor interacting mechanisms might operate in other LK signaling systems.

Notably, our ALKR mutagenesis experiments are consistent with the idea that hydrophobic interactions, H-bonds, and amide-pi stacking interactions may be involved in receptor activity and support the effectiveness of our molecular modeling. Our cell surface receptor expression experiments (Fig. S14) showed no significant changes for the expression of the mutant receptors compared with that of the ALKR, indicating that the changes in the activation of mutant receptors by ALK1 (Figs. 8 and S13) were not a result of changes in receptor expression. Overall, our computational and experimental evidence provided an account for specific roles of F, W, and amidation (affecting the bound confirmation) (Fig. 7, *C*–*E*). Specifically, F3 (Fig. 1) appears to be essential for the maintenance of the active conformation of ligands, as ALK1_A3 is not predicted to adopt the same conformation as active analogs when in the binding pocket. Similarly, the critical W6 residue of the ALK peptide is predicted to be involved in hydrophobic interactions (with I329) and H-bonds (with Q317) with ALKR. In cell-based assays, when either the nonpolar I329 was mutated to a polar Q or the polar Q317 was mutated to a nonpolar A, the experimental EC_50_ was increased. The I329Q mutation (EC_50_ = 6800 nM) had a much larger effect on EC_50_ than the Q317A mutation (EC_50_ = 56 nM), suggesting hydrophobic interactions involving W6 may be more critical for activity than possible H-bonding interactions.

Amidation is the most common posttranslational modification of neuropeptides and peptide hormones (*e.g.*, see (13)). For many peptides, this modification not only improves the stability and delivery of the peptide but is also necessary for its activity. At present, there are few crystal structures of a GPCR bound to an amidated peptide demonstrating direct interactions between the peptide C-terminal amide and the receptor. A validated homology model of amidated CCK4 with CCKR2 (*i.e.*, cholecystokinin B receptor) suggests that the amidated C-terminus forms an H-bond with the receptor (13, 95). The only currently available crystal structure is the bound conformation of amidated CCK8 with cholecystokinin A receptor, which directly demonstrated the presence of this H-bond (96). By contrast, our docking results did not find a specific interaction of the amidated C-terminus with ALKR. However, ALK1-OH, an ALK1 analog lacking C-terminal amidation, was not able to activate ALKR and does have difference in some interactions with ALKR, for example, missing the H-bond with Q317. Thus, the C-terminal amidation of different peptide ligands might have distinct roles during interactions with their receptors.

To examine the relevance of our work to other amidated peptide receptors, we also compared our findings with previous receptor comparison studies (13, 15) (See Supporting Results and Discussion and Table S7). Our results are consistent with a bound orientation of ALK peptides in which both the N- and C-termini of the peptide ligand are within the putative-binding pocket of the receptor, with the N-terminus pointing deep in the pocket. These results contrast with several other known examples of neuropeptide–receptor interactions where one terminus is critical for receptor activation and this terminus is buried deep in the binding pocket (13). In these cases, the critical terminus is oriented toward the receptor while the other terminus points toward solution. In our case, we showed that amidation at the C-terminus is essential for ALK’s activity in cell-based assays. Modeling then predicted that although the C-terminus is found in the binding pocket, it is oriented toward the extracellular region of the receptor whereas the N-terminus extends deeper into the pocket. There are a few other neuropeptides whose amino acids are all within the receptor-binding pockets, for example, thyrotropin-releasing hormone, which might be similar to ALKs. In the future, it will be interesting to determine how broadly the predicted bound conformations of ALKs are applicable to other peptide–receptor interactions.

We do note that although our work is relatively complete in its own right, there are outstanding issues that warrant future investigation. For example, can ALKR couple to G proteins other than those in the Gα_q_ family? Would ALKR behave similarly in cell lines other than CHO-K1 cells we used, for example, in *Drosophila* Schneider 2 cells (see also “IP1 accumulation assay” section in Experimental procedures)? What is the distribution of ALKR-positive neurons in the CNS of *Aplysia*? The answers to these and other questions could add important information to the mechanisms and functional roles of the LK signaling system in protostomes in general and in *Aplysia* in particular.

In summary, we have identified a LKR in *Aplysia* and studied ligand–receptor interactions using AI-based protein structure prediction, docking software, and mutagenesis of the receptor. Our results demonstrate how specific residues in both the peptide ligands and the receptor influence receptor activity, and molecular modeling predicts hydrophobic interactions, H-bonds, and amide-pi stacking that may be mediating this peptide–receptor interaction. We expect that our approach could be readily applied to other neuropeptide signaling systems, particularly to protostome peptide signaling systems that do not have homologs in deuterostomes. As more signaling systems in both protostomes and deuterostomes are being studied with an improved paradigm, we will gain a better insight on how broadly applicable the specific interactions we identified here are and on how diverse the operations of neuromodulatory systems in both protostomes and deuterostomes are.

## Experimental procedures

### Subjects

Experiments were performed on mollusc *A. californica* (100–350 g) obtained from Marinus. *Aplysia* are hermaphroditic (*i.e.*, each animal has reproductive organs normally associated with both male and female sexes). Animals were maintained in circulating artificial seawater at 14 to 16 °C and the animal room was equipped with a 12:12 h light-dark cycle with light period from 6:00 AM to 6:00 PM. All chemicals were purchased from Sigma-Aldrich unless otherwise stated.

### Bioinformatic analysis of peptide precursors and receptors

Initially, we used NCBI to search specific sequences of interests. In addition, we also searched AplysiaTools databases (Dr Thomas Abrams, University of Maryland (58)) to obtain additional sequences for comparison. These latter databases (http://aplysiatools.org) include databases for *Aplysia* transcriptome and *Aplysia* genome.

The ORFs of the putative receptor full-length cDNA sequence was obtained using ORF Finder (https://www.ncbi.nlm.nih.gov/orffinder/). We compared the LK peptides and receptor sequences with those of other species using BioEdit software (https://bioedit.software.informer.com/7.2/). Sequence logo plots (aligned from C-terminus) for LK peptides were generated using a Weblogo software (http://weblogo.berkeley.edu/logo.cgi). For the putative ALKR, TMs were predicted using TMHMM Server v. 2.0 (82, 83) (http://www.cbs.dtu.dk/services/TMHMM/). The phylogenetic trees of sequences from different species were constructed by MEGA X software (https://www.megasoftware.net/) using alignment by Clustal W and the maximum likelihood method with 1000 replicates, and JTT+G method was performed (Fig. 3). The selection of the models was based on the results of MEGA analysis.

### Cloning of receptor mRNA in Aplysia

#### RNA extraction

After anesthesia with 30 to 50% of the body weight with 333 mM MgCl_2_, *Aplysia* cerebral, pleural-pedal, buccal, and abdominal ganglia were dissected out and maintained in artificial seawater containing the following: 460 mM NaCl, 10 mM KCl, 55 mM MgCl_2_, 11 mM CaCl_2_, and 10 mM Hepes, pH 7.6, in a dish lined with Sylgard (Dow Corning). RNA was prepared from the *Aplysia* ganglia using the Trizol reagent method. Specifically, the dissected ganglia were placed into 200 μl Trizol (Sigma, T9424) and stored at −80 °C until use. The frozen ganglia in Trizol were thawed and homogenized with a plastic pestle, then Trizol was added to a total volume of 1 ml, which were incubated at room temperature for 10 min. Then, 200 μl chloroform was added, and the solution was mixed thoroughly by shaking and incubated on ice for 15 min. The solution was centrifuged (12,000*g*, 4 °C, 15 min), and the supernatant was added to an equal volume of isopropanol. The tube was shaken gently by hand and let stand at −20 °C for 2 h. After 2 h, the solution was centrifuged (12,000*g*, 4 °C, 15 min) again, the supernatant was discarded, and 1 ml of 75% ethanol/water was added, and the centrifuge tube was shaken gently by hand to suspend the pellet. The tube was centrifuged (12,000*g*, 4 °C, 10 min), the supernatant discarded, and the precipitant was dried at room temperature for 5 to 10 min. Finally, 30 μl of nuclease-free water was added to dissolve the RNA pellet, and the RNA concentration was determined with a Nanodrop ND-1000 spectrophotometer (Thermo Fisher Scientific).

#### Reverse transcription

Using the above extracted RNA as a template, cDNA was synthesized by reverse transcription using PrimeScript RT Master Mix Kit (Takara, RR036A) according to the instructions and then stored at −20 °C until use. The synthesized first-strand cDNA serves as a template for PCR.

#### PCR

The synthesized cDNA above was used as a template for PCR. Each pair of specific primers was designed (Table S3) in Primer Premier 6 and Oligo7, based on protein coding sequences for the putative receptor. The PCR reaction was performed with 98 °C/2 min predenaturing, 98 °C/10 s denaturing, ∼64 °C/15 s annealing, 72 °C/30 s extension, and 72 °C/5 min re-extension for 35 cycles. The PCR products were subcloned into vector pcDNA3.1(+) and sequenced to ensure the sequences were correct.

### IP1 accumulation assay

IP1 accumulation assay measures concentration of IP1 (88), that is hydrolyzed from the second messenger, inositol trisphosphate, generated by Gα_q_ pathway when GPCR expressed in CHO-K1 cells is activated by an appropriate ligand. We note two reasons that we selected mammalian CHO-K1 cells for assays for ligand-receptor (GPCR) activity: (1) there are no established molluscan cell lines available; (2) CHO-K1 cells have been extensively utilized not only for vertebrate GPCRs (*e.g.*, (97)) but also for invertebrate/protostome GPCRs, including other molluscs (9, 11, 73), annelids (8), jellyfish (98), *Caenorrhabditis elegans* (99), and arthropods (76, 78, 79, 80). Given that LKRs are only present in protostomes (6, 7, 66, 67), an additional advantage of using mammalian cells to assay ligand-receptor activity is that the effects of a possible native LKR in protostome cell lines could be excluded.

In order to express the *Aplysia* putative receptor transiently in CHO-K1, the cDNA was cloned into the mammalian expression vector pcDNA3.1(+). CHO-K1 cells (Procell, CL-0062) were cultured in F-12K medium (Gibco, 21127-022) with 10% fetal bovine serum (FBS, Genial, G11-70500) at 37 °C in 5% CO_2_. Transfection experiments were performed when the cells were grown to 70 to 90% confluence. For the ALKR, in each dish (60-mm diameter), 4 μg of the putative receptor plasmids [in pcDNA3.1(+)] were mixed with 400 μl of Opti-MEM (Gibco, 11058021), followed by the addition of 15 μl of Turbofect (Thermo Fisher Scientific, R0531). For the *Anopheles* LKR, we could not obtain IP1 responses with the above procedure, suggesting that this receptor may not associate with the native Gα_q_ in CHO-K1 cells. Thus, 3 μg of the putative receptor plasmids [in pcDNA3.1(+)] and 3 μg of promiscuous Gα_q_ protein (89) [in pcDNA3.1(+)] were cotransfected in the above procedure for the *Anopheles* LKR (8, 10). The CHO-K1 cells with the reagents added above were mixed gently and incubated at room temperature for 15 min. The DNA/Turbofect mixture dropwise was then added to the dish, and the cells were incubated at 37 °C in 5% CO_2_ overnight. The next day, the cells were trypsinized and reseeded in opaque white 96-well half-area (Corning, 3688) or 384-well tissue culture–treated plates (Corning, 3570) at a density of 20,000 cells/well in F-12K and 10% FBS and incubated at 37 °C in 5% CO_2_ overnight. On the third day, the activation of the receptors was detected by monitoring IP1 accumulation using IP1 detection kit (Cisbio, 62IPAPEB) with a Tecan Spark plate reader. Except for using 0.5 × reagents for the anti-IP1-cryptate and IP1-d2 reagents, all other procedures were performed in accordance with the IP1 detection kit manufacturer’s instructions.

### Molecular modeling of the interactions of peptide ligands and the receptor

The topology files of peptide ligands, that is, ALK1, ALK2, and ALK1 analogs, were synthesized with SYBYL X-2.0 and optimized with Amber FF99SB force field. Max Iteration = 100,000, gradient = 0.005. The topology file of the receptor was predicted by Robetta Server (http://robetta.bakerlab.org/). Five receptor structures which differ primarily at the N- and C-termini were obtained from Robetta, and we selected the best model. The quality of the generated model was evaluated using the online servers of QMEAN (https://swissmodel.expasy.org/qmean) and PROCHECK v.3.5.4 (https://saves.mbi.ucla.edu/) (Supporting Results and Discussion).

Autodock Vina is used for semi-flexible docking, and all ligands adopt the same parameters for docking. We set a grid box (37.5 Å × 37.5 Å × 37.5 Å) centered at (36.787, −2.312, −15.122) Å. Due to flexibility of the peptide ligands, we modified the following two parameters: modes = 1000, exh. = 10. Other parameters were set to default. We performed molecular docking five times for each peptide and made sure the conformations that had the lowest affinity energy are similar at least four times. We then used the similar one for further analysis.

### Mutagenesis of the ALKR

In a first set of experiments, mutagenesis of the ALKR was performed without FLAG tag (Fig. 8). Specifically, construction of the ALKR mutants was performed employing the full-length ALKR cDNA cloned into the pcDNA3.1(+) plasmid. Site-directed mutagenesis was performed using the site-directed mutagenesis kit (Sangon Biotech), following the manufacturer’s instructions. Briefly, forward and reverse primers containing the expected mutation were mixed with kit components, and 10 ng of pcDNA3.1(+)-ALKR was used as the mutation template. After 14 to 18 rounds of PCR amplification, 1 μl of DpnI was added and incubated at 37 °C for 1 h in order to digest the template. The primers used to obtain the mutants were designed based on the ALKR cDNA sequence and listed in Table S3. Mutants were confirmed *via* DNA sequencing. IP1 accumulation assay with ALKR and mutants without FLAG tags (Fig. 8) was performed using procedures described in “IP1 accumulation assay” section.

In order to determine receptor expression, we added a FLAG tag to the ALKR and mutant receptors by taking advantage of the recombinant plasmids of pcDNA3.1(+)-ALKR (ALKR or mutants) we constructed above. For each mutant, the pcDNA3.1(+)-ALKR recombinant plasmid was first cleaved with the restriction enzymes HindⅢ and ApaⅠ to obtain the DNA sequence of the receptor. The cleavage product was subcloned into vector pcDNA3.1(+)-FLAG to obtain pcDNA3.1-FLAG-ALKRs (ALKR or mutants) recombinant plasmids (FLAG tag at the N-terminus of each respective receptor). The final products were confirmed by DNA sequencing.

To perform IP1 accumulation assay with the receptors with FLAG tags (Fig. S13), transfection experiments with recombinant plasmids were performed in accordance with the jetPRIME (Polyplus Transfection, PT-114-15) manufacturer’s instructions. In each well (6-well plates), 2 μg of the ALKR (ALKR or mutants) recombinant plasmids [in pcDNA3.1(+)-FLAG] were mixed with 200 μl of jetPRIME buffer, followed by the addition of 4 μl of jetPRIME and incubated at room temperature for 10 min. The DNA/jetPRIME mixture dropwise was then added to the dish. The cells were incubated at 37 °C in 5% CO_2_ overnight. Other procedures followed “IP1 accumulation assay” section described earlier.

### Cell surface expression analysis by ELISA

Cell surface expression determination of ALKR and mutant receptors, all with FLAG tags (Fig. S14), was performed using a procedure modified from previous work (100, 101, 102). Specifically, CHO-K1 cells (Procell, CL-0062) were maintained in F-12K medium (Procell, PM150910) with 10% FBS (Genial, G11-70500) at 37 °C in 5% CO_2_. Cells were cultured in 6-well plates (BIOFIL, TCP011006). When the cells were grown to 70 to 90% confluence, transfection experiments were performed in accordance with the jetPRIME (Polyplus Transfection, PT-114-15) manufacturer’s instructions. Briefly, in each well, 2 μg of the receptor plasmids [in pcDNA3.1(+)-FLAG] were mixed with 200 μl of jetPRIME buffer, followed by the addition of 4 μl of jetPRIME and incubated at room temperature for 10 min. The DNA/jetPRIME mixture dropwise was then added to the dish. The cells were incubated at 37 °C in 5% CO_2_ overnight. After 24 h, transfected cells were plated in 96-well white clear-bottom cell culture plates (Corning, 3610) at a density of 20,000 cells in 100 μl per well and incubated overnight. The following day, culture media was aspirated and cells were washed twice with 200 μl of 1× PBS (Procell, PB180327). Then, 100 μl of 1× PBS containing 5% (w/v) bovine serum albumin was added to each well and incubated at room temperature. After 30 min, 100 μl of 1:10,000 anti-FLAG M2-HRP conjugate (Sigma-Aldrich, Cat A8592) was added to each well and incubated for 30 min at 37 °C. Cells were washed twice with 200 μl of 1× PBS and then incubated with 200 μl of TMB Chromogen Solution (Sangon Biotech, E661007) for 30 min at 37 °C in the dark. Finally, 50 μl of ELISA Stopping Solution (Sangon Biotech, E661006) was added to each well to stop the reaction. The absorbance at 450 nm was measured using a Tecan Spark microplate reader. In each experiment, expression of the mutant ALKRs was assessed and compared with that measured for the ALKR in the same experiment.

### Peptide and DNA synthesis

Peptides were synthesized by Synpeptide Co, Ltd, Guoping Pharmaceutical Co, Ltd, or ChinaPeptides Co, Ltd (Fig. S15) and were aliquoted in 50 nmol per microcentrifuge tubes, stored at −20 °C until use. The DNA sequence of *Anopheles* LKR (Fig. S15) was synthesized by Tsingke Biotechnology Co, Ltd and was stored at −20 °C until use.

### Data and statistical analyses

Dose-response curves and bar graphs for experimental data were plotted using Prism software (GraphPad). Data are expressed as the mean ± SEM. Final EC_50_ values are rounded to two significant figures. All experimental data were taken from individual animals or preparations, and n refers to the number of preparations unless otherwise stated. Statistical tests were performed using Prism software. They included Student’s *t* test, one-way ANOVA, as appropriate. Data that showed significant effects in ANOVA were further analyzed in individual comparisons with Bonferroni’s correction.

## Data availability

Structural model and docking result files used to generate Figure 7 are available on github (https://github.com/li-yadong/ALKR-paper). All other data are included in this article and the supporting information. The nucleotide sequence(s) reported in this article has been submitted to the GenBank/EBI Data Bank with accession number(s): OP292655.

## Supporting information

This article contains supporting information, and there are the reference citations which are located in the supporting data file.

## Conflict of interest

The authors declare that they have no conflicts of interest with the contents of this article.

## Supporting information

Supporting Results and Discussion and Figures S1–S14
