# Supplementary material for: AI protein structure prediction-based modeling and mutagenesis of a protostome receptor and peptide ligands reveal key residues for their interaction
Source: J Biol Chem. 2022 Aug 30;298(10):102440. doi: 10.1016/j.jbc.2022.102440 (PMC9562341; doi:10.1016/j.jbc.2022.102440)
Supplement: Supporting Results and Discussion and Figures S1–S14 [file mmc1.docx]

**Supporting Information**

**AI protein structure prediction-based modeling and mutagenesis of a protostome receptor and peptide ligands reveal key residues for their interactions**

**Shi-Qi Guo^1,^**^‡^**, Ya-Dong Li^1,^**^‡^**, Ping Chen^1,^**^‡^**, Guo Zhang^1,^**^‡^**, Hui-Ying Wang^1^, Hui-Min Jiang^1^, Wei-Jia Liu^1^, Ju-Ping Xu^1^, Xue-Ying Ding^1^, Ping Fu^1^, Ke Yu^1^, Hai-Bo Zhou^2,3,^*****, James W. Checco^4,^*****, Jian Jing^1,3,5,^***

*From the ^1^State Key Laboratory of Pharmaceutical Biotechnology, Institute for Brain Sciences, Chinese Academy of Medical Sciences Research Unit of Extracellular RNA, Jiangsu Engineering Research Center for MicroRNA Biology and Biotechnology, Advanced Institute for Life Sciences, Chemistry and Biomedicine Innovation Center, School of Life Sciences, Nanjing University, Nanjing, Jiangsu 210023, China; ^2^School of Electronic Science and Engineering, Nanjing University, Nanjing, Jiangsu 210023, China; ^3^Peng Cheng Laboratory, Shenzhen 518000, China; ^4^Department of Chemistry and the Nebraska Center for Integrated Biomolecular Communication (NCIBC), University of Nebraska-Lincoln, Lincoln, NE 68588, USA; ^5^Department of Neuroscience and Friedman Brain Institute, Icahn School of Medicine at Mount Sinai, New York, NY 10029, USA*

‡ These authors contributed equally to this work.

* For correspondence: Jian Jing, [jingj01@live.com](mailto:jingj01@live.com); Hai-Bo Zhou, [haibozhou@nju.edu.cn](mailto:haibozhou@nju.edu.cn); James W. Checco, [checco@unl.edu](mailto:checco@unl.edu).

There is Supporting Results and Discussion in this document. Additionally, there are a total of fifteen supporting figures (Figs. S1-S15) and six supporting tables. Figs. S1-S14 are included in this document, whereas Fig. S15 is included as a separate PDF document with its legend provided below. The six supporting tables are included as six separate Excel files (Table S1-S6.xlsx), whereas the legends for the tables are provided below.

Figure S15. Peptide and DNA (*Anopheles* LKR) synthesis information from the commercial companies.

Table S1. LKR sequences for Fig. 3;

Table S2. Putative post-translational modifications (PTMs);

Table S3. Primers;

Table S4. ALKs and LKs for Fig. 1

Table S5. Statistical results for Fig. 4;

Table S6. List of docking results;

Table S7. List of polar residues conserved for amidated peptide receptors (Tikhonova et al, 2019) and comparison with those in ALKR

**A note on the selection of transfection reagents**:

In the earlier experiments, we used Turbofect (Thermo Fisher Scientific, R0531) for the transfection of plasmids. However, during the more recent experiments carried out for Fig. S6, S13 and S14, we used a new transfection reagent (jetPRIME) (see Experimental procedures) because Turbofect is no longer available in China. Based on the results generated from Fig. S6 and S13, the two transfection reagents made no apparent difference on EC_50_ measurement in the IP1 accumulation assay experiments.

**Contents**

Supporting Results and Discussion 4

Possible post-translational modifications in ALKR 4

Validation of the ALKR predicted model 4

Docking of the ALKR with ALK1 analogues and comparisons of specific residues with other protostome LK receptors 5

Putative ligand-receptor interactions: Hydrophobic interactions, H-bonds and amide-pi stacking interactions 6

Comparison with structural work on receptors for other amidated peptides 6

Supporting Figures 8

Figure S1. *Aplysia* LKR is a GPCR. 8

Figure S2. cDNA and protein sequence of ALKR. 9

Figure S3. Cloning of the putative ALK receptor. 10

Figure S4. All of peptides generated from ALK precursor. 11

Figure S5. Summary of activation of the ALKR by native ALK peptides. 12

Figure S6. Selectivity of ALK1. 13

Figure S7. Structure of the ALKR as predicted by Robetta. 14

Figure S8. PROCHECK-Ramachandran plot of the ALKR predicted model. 15

Figure S9. The main-chain parameters of ALKR modeled structure. 16

Figure S10. The side-chain parameters of ALKR modeled structure. 17

Figure S11. Comparison plot indicates the quality of the model in comparison with experimental structures of other proteins with similar sizes. 18

Figure S12. Comparison of ALKR with LKR sequences in other species illustrating the possibly conserved residues that may be interacting with LKs. 20

Figure S13. Mutagenesis of specific residues in the ALKR and mutant receptors containing FLAG tags at the N-termini. 21

Figure S14. Cell surface expression of ALKR and 10 mutant receptors with FLAG tag. 23

Supporting references 24

Supporting Results and Discussion

Possible post-translational modifications in ALKR

We analyzed sites for possible post-translational modifications in the ALKR (Table S2). Two Cysteine residues, located in the extracellular loop 1 (ECL1) (C149) and the ECL2 (C227), are expected to form a disulfide bond to stabilize the receptor. Analysis with NetNGlyc 1.0 server (1) (https://services.healthtech.dtu.dk/service.php?NetNGlyc-1.0) identified five potential N-glycosylation sites at asparagine residues (N27, N53, N62, N161, N219) with NXS/T (Asn-Xaa-Ser/Thr) motif. However, because N161 is found within a transmembrane helix (TM3), it is not expected to be glycosylated. NetPhos 3.1 server (2) (https://services.healthtech.dtu.dk/service.php?NetPhos-3.1) identified potential phosphorylation sites at serine, threonine and tyrosine residues, with a total of 42 potential phosphorylation sites. Among these sites, 8 serine residues (S20, S31, S33, S34, S37, S39, S40, S44) and one tyrosine residue (Y69) have phosphorylation potential scores of 0.9 or higher on a scale of 0-1. Cysteine residues C369, C371 and C391 could be palmitoylated, as is common for many GPCRs. Based on its structure predicted using Robetta (https://robetta.bakerlab.org/) (3) as used for structure-functional analysis described in a later section, C369 has the higher probability among the three cysteine residues.

Validation of the ALKR predicted model

The predicted structural model of ALKR from Robetta was evaluated with two programs. First, the ALKR model was validated by the PROCHECK-Ramachandran plot (4) (Fig. S8-S10). The model comprised of 391 (91.1%) non-glycine and non-proline residues of 429 amino acid, 23 (5.4%) glycine residues, 13 (3.0%) proline residues, and 2 (0.4%) end-residues (excl. Gly and Pro). The results demonstrated that 357 of 391 (91.3%) non-glycine and non-proline amino acids were located in the most favored regions; 28 (7.2%) in the additional allowed regions; 2 (0.5%) in the generously allowed regions; and 4 (1.0%) in the disallowed regions (Fig. S8). The overall average of the G-factors was 0.36, which was favorable. A low or negative G-factor is an unfavorable parameter in model quality estimation, and values less than -0.5 and -1 are interpreted as unusual and very unusual, respectively. Among the stereochemical parameters of the main chain, the Zeta angle standard deviation and overall G-factor represented better quality compared to the ideal values and the remaining four properties were inside the suitable regions (Fig. S9). Besides, all of the side-chain stereochemical parameters also showed better quality in comparison with the ideal values (Fig. S10).

Second, model quality estimation by QMEAN (5) provided a QMEAN Z-score of -2.03. Fig. S11 is a comparison plot that shows the ALKR model quality score in comparison with experimental structures of similar size. A QMEAN Z-score of -2.03 lies in the range for “good” and “medium” models (5), and slightly below the majority of structures in the PDB reference set (Fig. S11). It should be noted that QMEAN scores are designed primarily for soluble proteins, and membrane proteins are known to deviate from the score distributions as soluble proteins (5). Several prior studies modeling membrane proteins have yielded QMEAN Z-scores similar to that of our model (6-9).

Docking of the ALKR with ALK1 analogues and comparisons of specific residues with other protostome LK receptors

The ineffective analogues can be classified into two groups based on their predicted interactions with the receptor. First, ineffective analogues ALK1_A6 (W6 replaced by A) (Fig. 7*C*) and ALK1-OH (no C-terminal amidation) (Fig. 7*D*) also bound to the receptor in the similar conformation with the active ones, but lacked the same binding site interactions (i.e., H-bonds) with Q317. Specifically, ALK1_A6 formed H-bonds with Y213 and Q133, formed hydrophobic interaction with L157. ALK1-OH formed H-bonds with Y213, Q133 and C227, formed hydrophobic interactions with L157 and I329. Neither of these two analogues was predicted to form H-bonds with Q317 (even though ALK1-OH had a W6), suggesting that Q317 could be important for the activity of the receptor. Second, the other ineffective analogue, ALK1_A3 (F3 replaced by A), did not bind to ALKR in the common conformation. Instead, it was predicted bind to ALKR in a different conformation and forms two H-bonds with N330 and Q311 (Fig. 7*E*), and these H-bonds are also different from the above (e.g., lacking the H-bond with Q317). The inability to form the common conformation was probably partly due to the fact that, in the effective analogous, F3 side chain formed hydrophobic interaction with L157 to maintain the active conformation. In addition, p-pi conjugates of Q250 and Q311 side chain (amidations) and pi-pi conjugate of F3 (aromatic ring) might form amide-pi stackings when the effective analogues bound to the receptor with the active conformation, which likely played a role in maintaining conformation

To determine whether the critical residues in the ALKR might be of general significance, we compared the ALKR and 12 LKRs in other species in BioEdit (Fig. S12). Several amino acids in the ALKR that interacted with ALK1 and other effective ligands were mostly conserved or had similar properties in LKRs of other species. For example, the amidation oxygen of Q317 of ALKR near helix 6 formed an N-H…O type H-bond with indole nitrogen of W6 of ALK1, ALK2, and their analogues. Near this position is N, which has a similar property as Q, in lymnokinin receptor, which is closely related to ALKR. Among the other functionally identified leucokinin receptors, in *A. aegypti*, *A. stephensi* and *D. melanogaser* were T, in *R. microplus* was Q at this position. In these 13 leucokinin receptors (including ALKR), there were 7 Q, 1R. In other cases, there were amino acids with similar characteristics one or two amino acid away: 4 Y, 1 N. Most of these residues (Q, N, T) had oxygen atoms (whereas it is nitrogen atom in R) to form H-bonds in their side chains. Q133 of ALKR at TM2 forms N-H…O type H-bond with P1 of these ligands (A of ALK1_A1), in which oxygen atoms of P/A in the peptide chain contributed to lone pair electrons. I329 near TM7 of ALKR formed hydrophobic interaction with W6 of ligands, respectively. Q250 at TM5 and Q311 at TM6 of ALKR formed amide-pi stacking with ligands, and the positive center (C atom) of amidation in Q250 and Q311 side chain were attracted to the negative center (benzene ring plane) of F3. The above four amino acids (Q133, Q250, Q311 and I329) are identical in their corresponding positions of other LKRs. L157 at TM 3 of ALKR also formed hydrophobic interaction with the ligands. There are L (3 receptors), V (5 receptors) or A (3 receptor) in this position, and all of these are non-polar amino acids. In other cases, there were amino acids with similar characteristics one or two amino acid away: i.e., V (2 receptors). Y213 at TM4 of ALKR formed N-H…O type H-bond with S5 (A in ALK2), the oxygen atom in Y side chain contributed to the lone pair electrons. At this position were Y in all mollusks and *C. teleta*, R in *A. cerana cerana* and *F. varia*. The others are non-polar amino acids (5 L, 1 F and 2 W).

Putative ligand-receptor interactions: Hydrophobic interactions, H-bonds and amide-pi stacking interactions

Hydrophobic interactions are among the most common type of protein-ligand interactions, often involving the contact of carbon with carbon, halogen or sulfur atoms. The most abundant type is formed by aliphatic carbon in the receptor and aromatic carbon in the ligand, such as the interactions of ALKs with L157 and I329. The difference is that I329 is located above the W heterocyclic plane (more common), while L157 is around the F benzene plane (less common).

H-bonds are a ubiquitous directional intermolecular interaction in biological complexes and contribute to the specificity of molecular recognition. The most common is N-H…O H-bonds, and ALKs interacting with Q133, Y213 and Q317 of ALKR are of this type. The bond lengths of the three H-bonds are not very different, at 1.9-2.2 Å, but the bond angles are different (Table S6). The bond angles of P3: Q133 is about 163°, W6: Q317 is about 142°, S5: Y213 is about 174°, and the optimal Angle of N-H…O H-bond is about 180°, which can partly explain the increase of EC_50_ when Y213 is mutated into A, and the effect is significantly greater than that of receptors with Q133 and Q317 individually mutated into A. However, when these three amino acids were mutated into A simultaneously, the EC_50_ showed much bigger increase, indicating that these three amino acids forming the H-bonds might have a synergistic effect (Fig 8*G*) on the activation of receptor.

Amide-pi stacking interactions are also common in ligand-protein binding, although less common than hydrophobicity and H-bonds (10). There are two types of amide-pi stackings: face to face and edge to face, respectively. According to the docking results, Q250 and Q311 interact with ALKs in the latter manner and mainly stabilize the active conformation of ligand by forming amide-pi stackings with the benzene ring of F3. However, because we performed semi-flexible docking, which means that the receptor molecule is rigid by default, that might not be the case if the movement of the side chain of these two residues is considered. In addition, the measured distances of these two amide-pi interactions are 4.1 Å for Q250 or 4.3 Å for Q311, which are slightly longer than the range specified by Ferreira de Freitas et al 2017 (10) but are in similar range as specified by Krone et al 2020 (11).

Comparison with structural work on receptors for other amidated peptides

Previous work has compared multiple vertebrate GPCRs for C-terminal amidated peptides and determined conserved polar residues that might be of importance for receptor activity (12) (see also Table S7 and reference (13)). Among these known neuropeptide receptors, there are three binding-site residues significantly conserved: asparagine or glutamine at positions 3.32, 4.60, and 6.55, with the conservation score of 66%, 44%, and 52%. Based on the comparisons of CCKBR (NCBI: NP_795344.1) and NPYR2 (neuropeptide Y receptor 2, NCBI: NP_001357109.1) in humans (as used in their analysis (12)) with the ALKR by BioEdit, these three residues correspond to L139, Y213 (H-bond in ALKR) and N314 of the ALKR, respectively, which are not exactly the same as the amino acids that bind to the ALK ligands in our docking results. Specifically, Y213 is similar, and is important in ALKR activity based on mutagenesis experiments (Fig. 8). L139 is actually a nonpolar residue and is located at ECL2 of ALKR. Because ALKs are small, and so are deep in the binding pocket in bound conformations, we expect that this residue likely does not play a major role. By contrast, although in the result of our molecular docking, N314 did not directly interact with the ligand, amidation in the N314 side chain might also be involved in the formation of H-bond or amide-pi stacking (see Results in the main text). We therefore mutated it and found that after replacing N314 with A, the EC_50_ of ALK1-receptor activation increased significantly (Fig. 8). Thus, although our molecular modeling is quite useful, there may be other residues, such as N314, that are important for receptor activation that we haven’t yet found.

There are additional conserved residues of other receptors, for example, a conserved residue in RF-amide receptors is D6.59 (12), which corresponds to Q317 on the ALKR. The mutation of this residue to A significantly attenuates peptide activity. From functional and modeling studies, it has been suggested that D6.59 interacts with the R of the peptide RF-amide motif, which is similar to our docking results. In our case, Q317 of ALKR forms hydrogen bonds with W6 of the active ALKs.

Supporting Figures


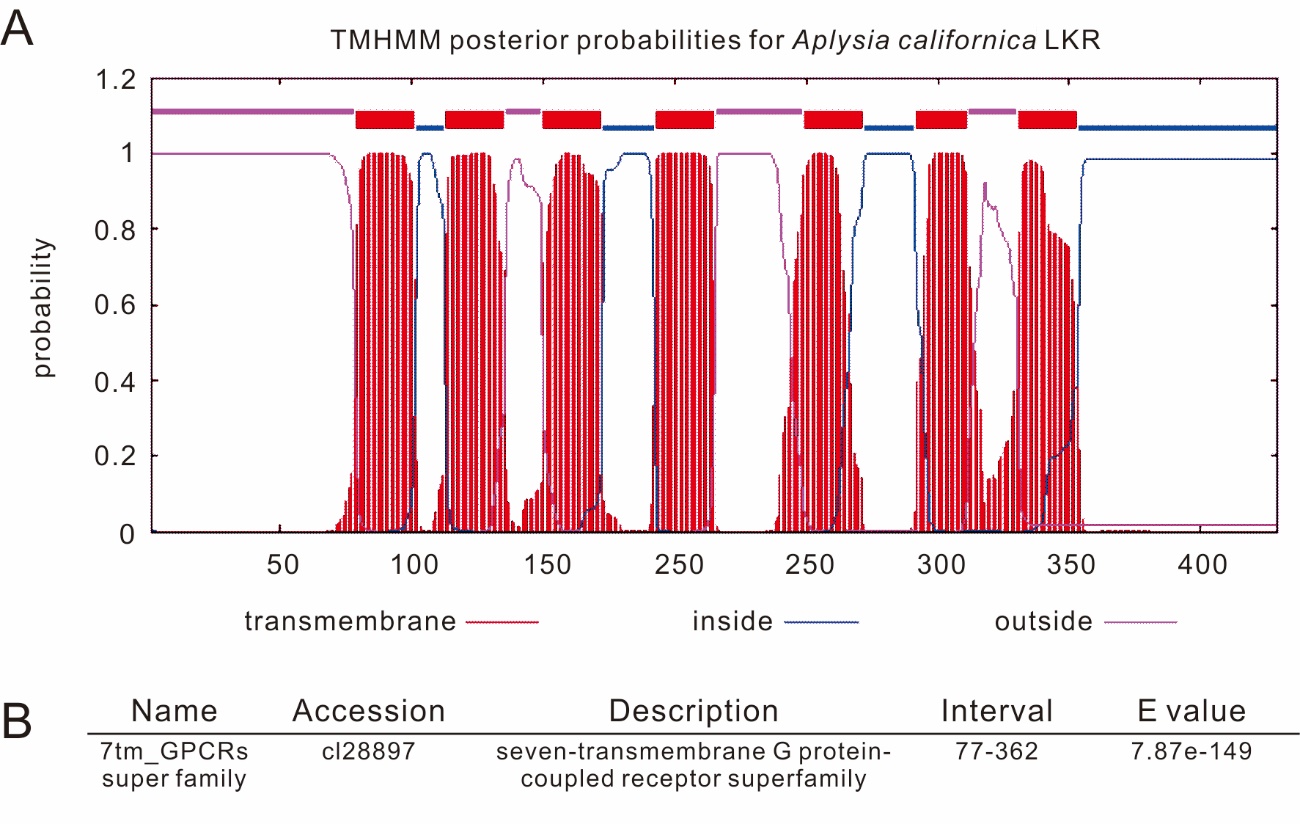


Figure S1. *Aplysia* LKR is a GPCR. TMHMM (*A*) and NCBI Conserved Domains Database (*B*) both predicted ALKR to be a GPCR with seven transmembrane domains.


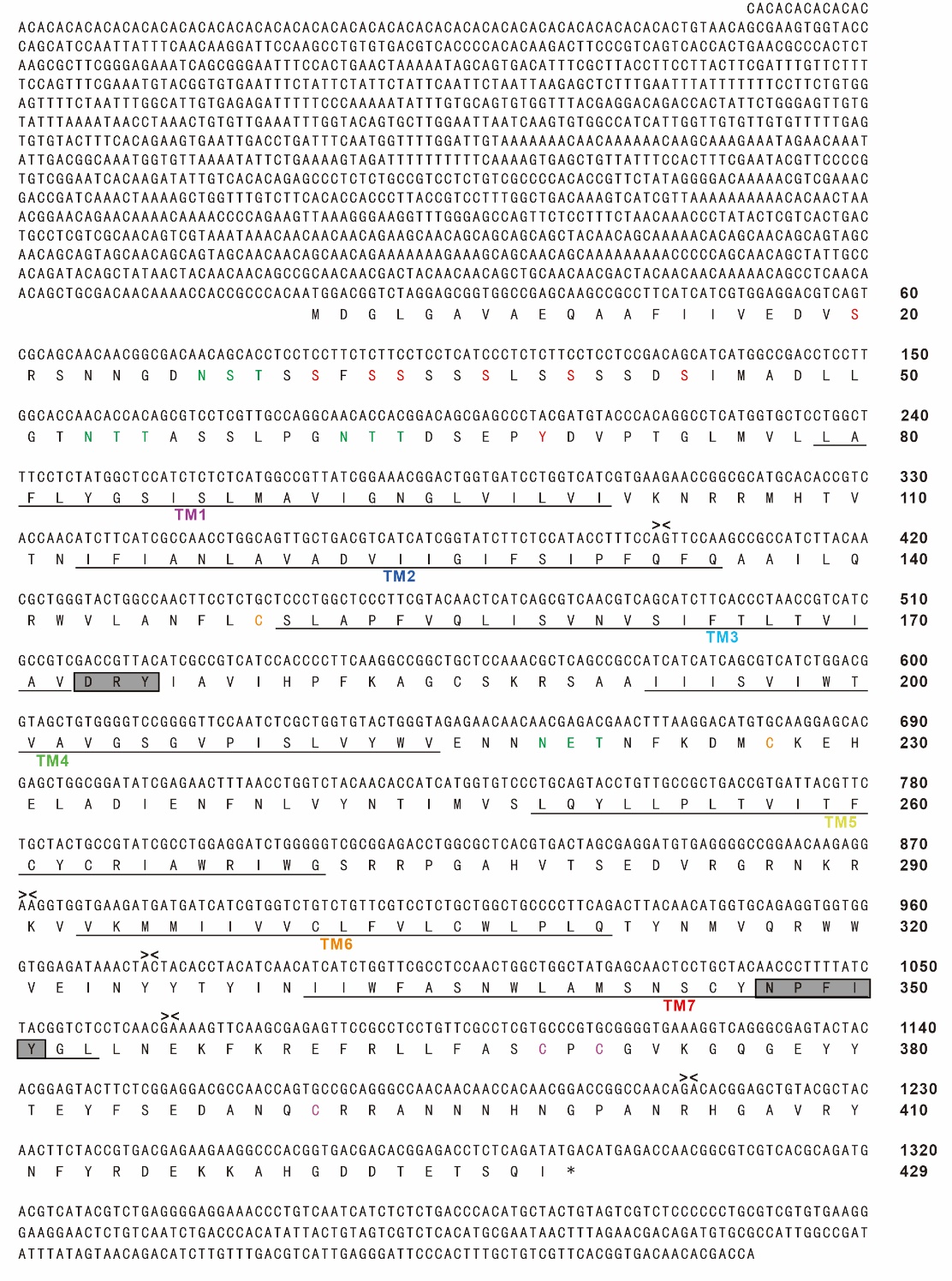


Figure S2. cDNA and protein sequence of ALKR. The open reading frame is 1290 bp long and encodes a protein with 429 amino acids. Predicted transmembrane domains (TMs) were underlined, while the potential positions of introns are marked by (><). Shaded boxes indicate motifs in TM3 (DRY) and TM 7 (NPFIY) that are conserved among class A GPCRs. Possible N-glycosylation sites (NXS/T) were shown in green, potential phosphorylation sites with high scores (> 0.9) were shown in red, two Cysteine residues that could form a disulfide bond (C149 and C227) were shown in orange, potential palmitoylation (C369, C371 and C391) sites were shown in purple.


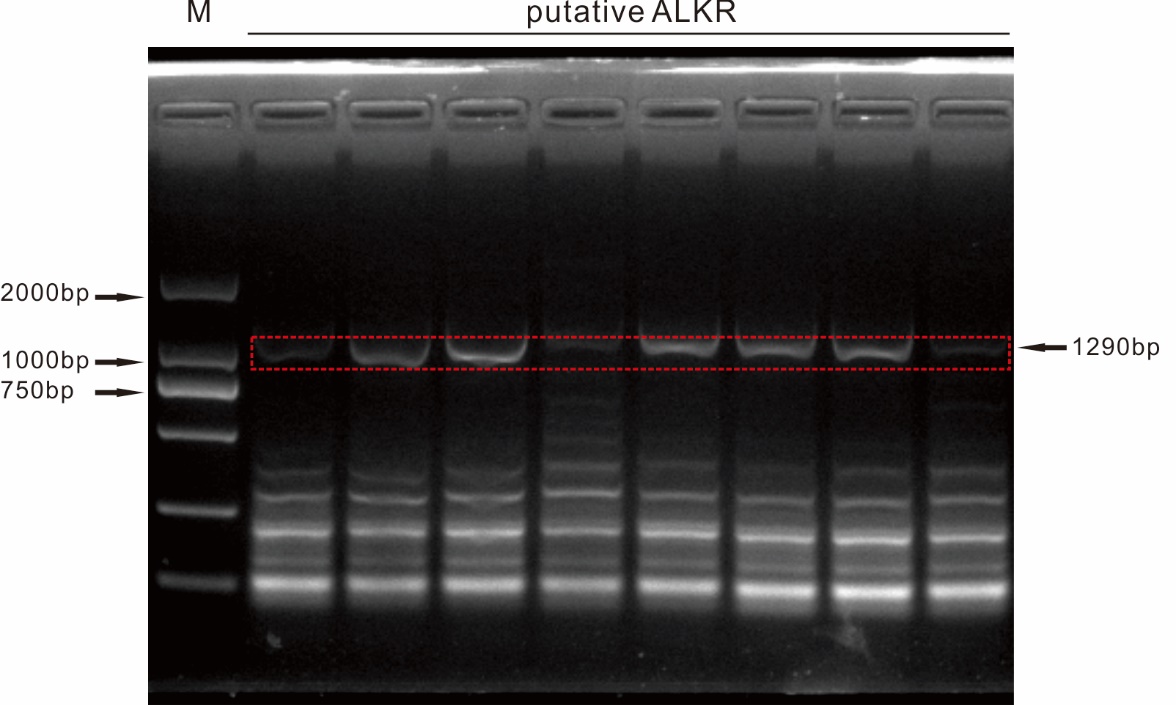


Figure S3. Cloning of the putative ALK receptor. A complete gel for the PCR product for the putative ALK receptor with a length of 1290 bp (marked by a red box). Lane 1 was DNA marker (M), the other lanes were all PCR products of putative ALK receptor.


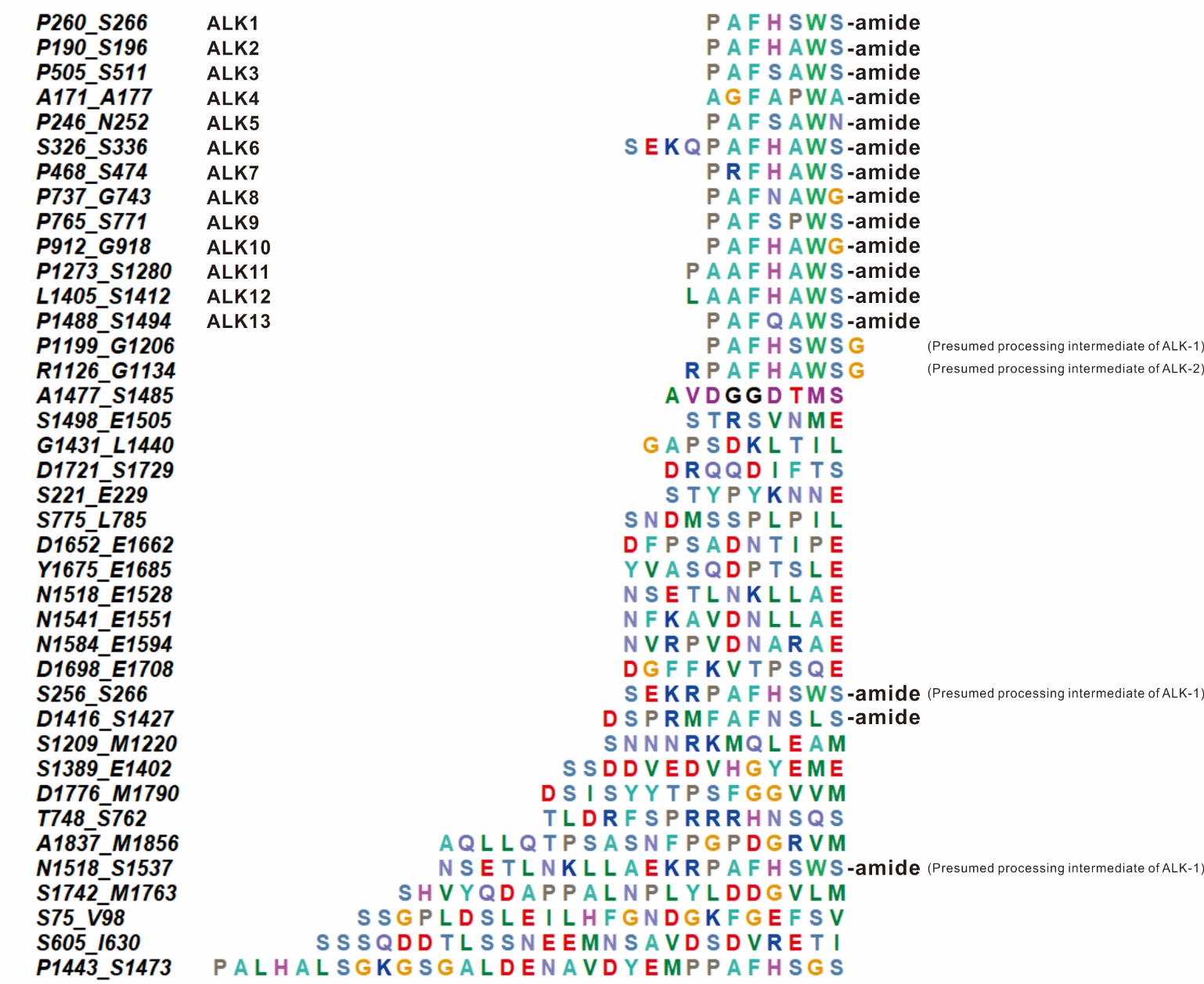


Figure S4. All of peptides generated from ALK precursor. Except for ALK1, ALK2 and ALK3 (P505_S511), other peptides were single copies from the precursor. The first 13 peptides (labelled as ALK1-13) are ALKs with the FXXWX-amide motif.


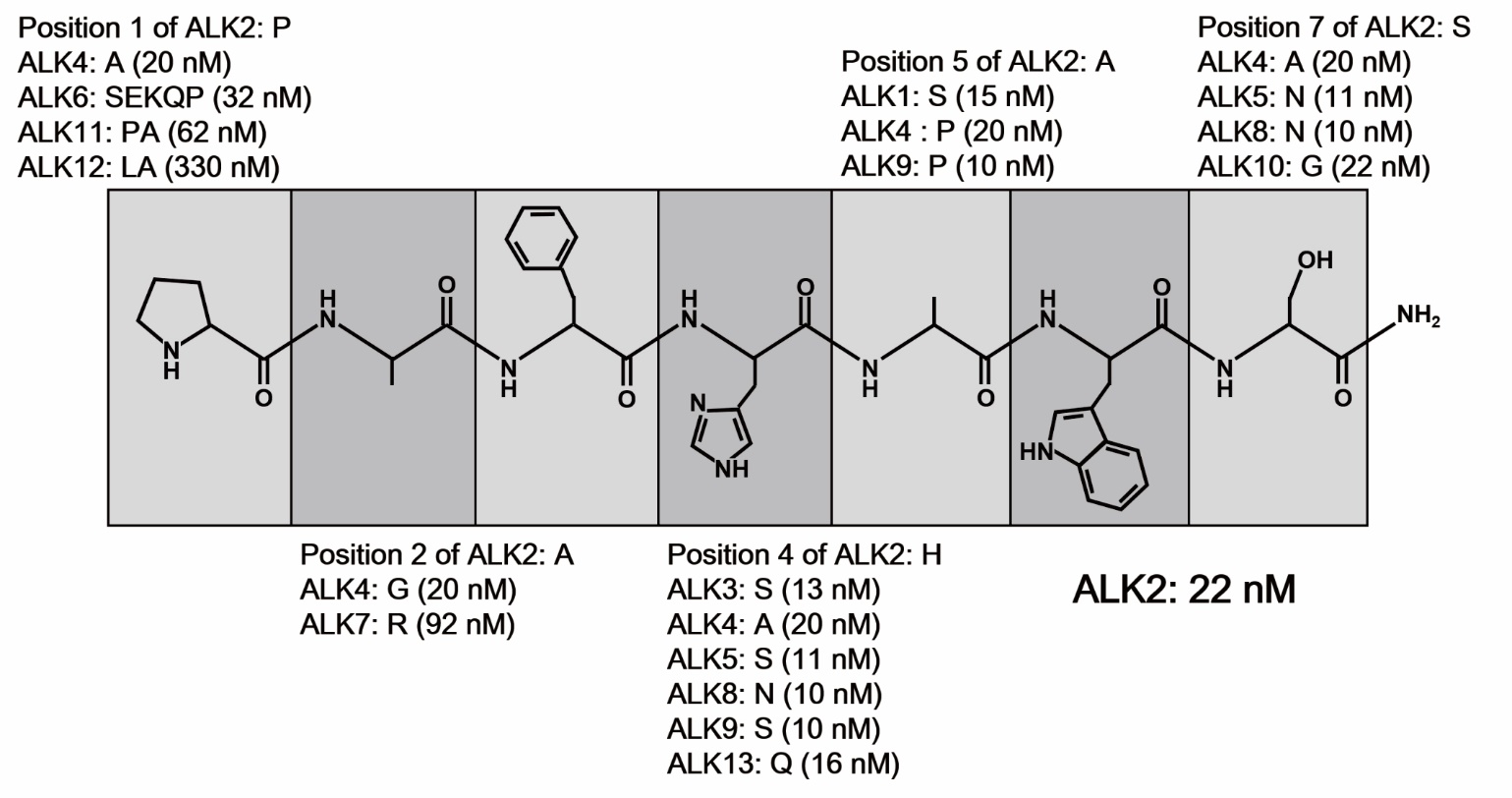


Figure S5. Summary of activation of the ALKR by native ALK peptides. EC_50_ values are shown for ALKs at the residue that is different from that in ALK2.


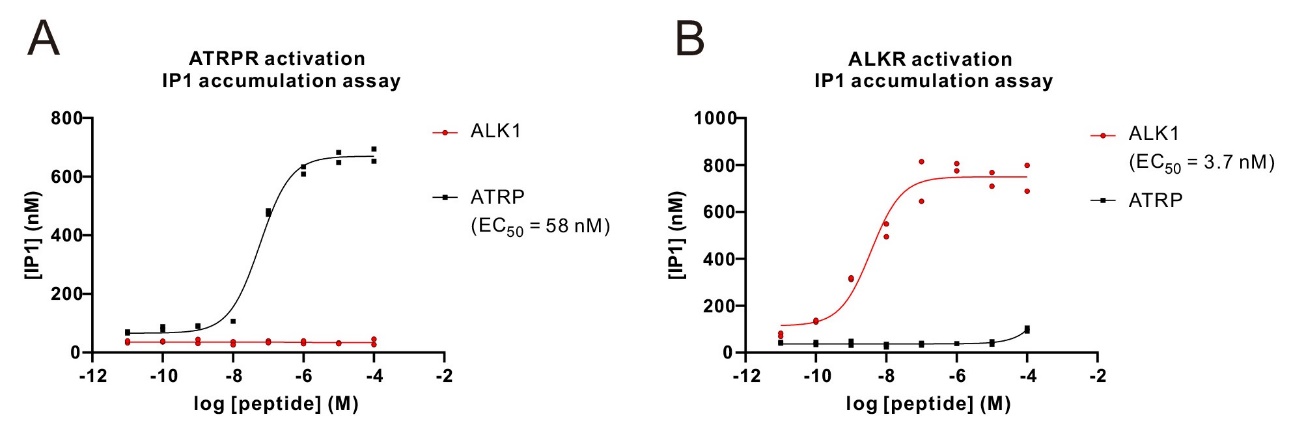


Figure S6. Selectivity of ALK1. *A,* Effects of ALK1 (PAFHSWS-amide) and ATRP (GFRLNSASRVAHGY-amide) on ATRPR. ATRP activates ATRPR, whereas ALK1 cannot. *B,* Effects of ALK1 and ATRP on ALKR. ALK1 activates ALKR, whereas ATRP cannot.


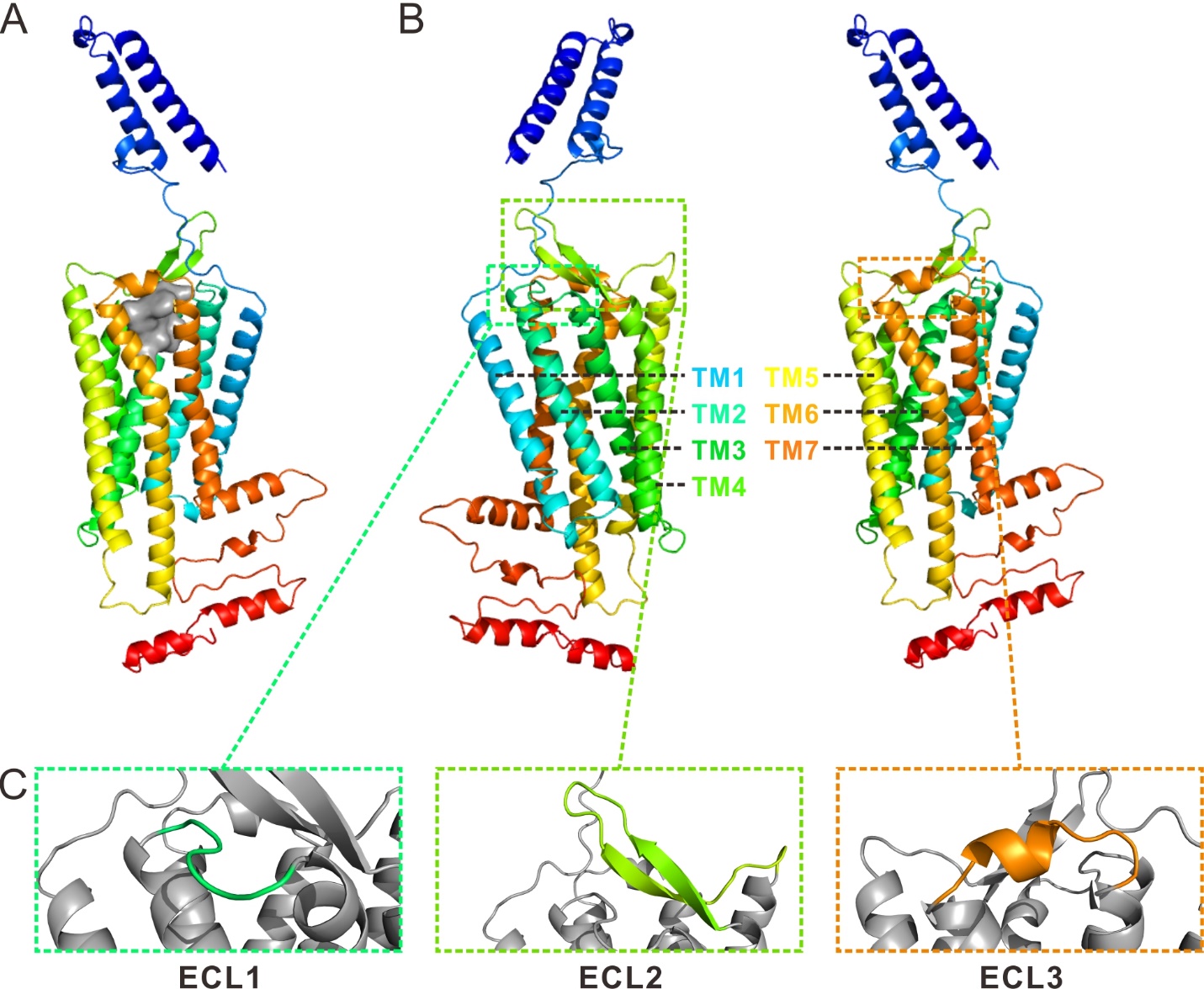


Figure S7. Structure of the ALKR as predicted by Robetta. *A,* Structure of the ALKR together with the ligand ALK1 (shown in grey) from docking results of Autodock Vino. *B,* Structure of the ALKR illustrating the transmembrane domain 1-7 (TM1-7). The view in the right panel is similar to the view in *A*. The view in the left panel is the opposite view of the right panel. *C,* Enlarged view of the ALKR in panel *B* illustrating the extracellular loop 1-3 (ECL1-3).


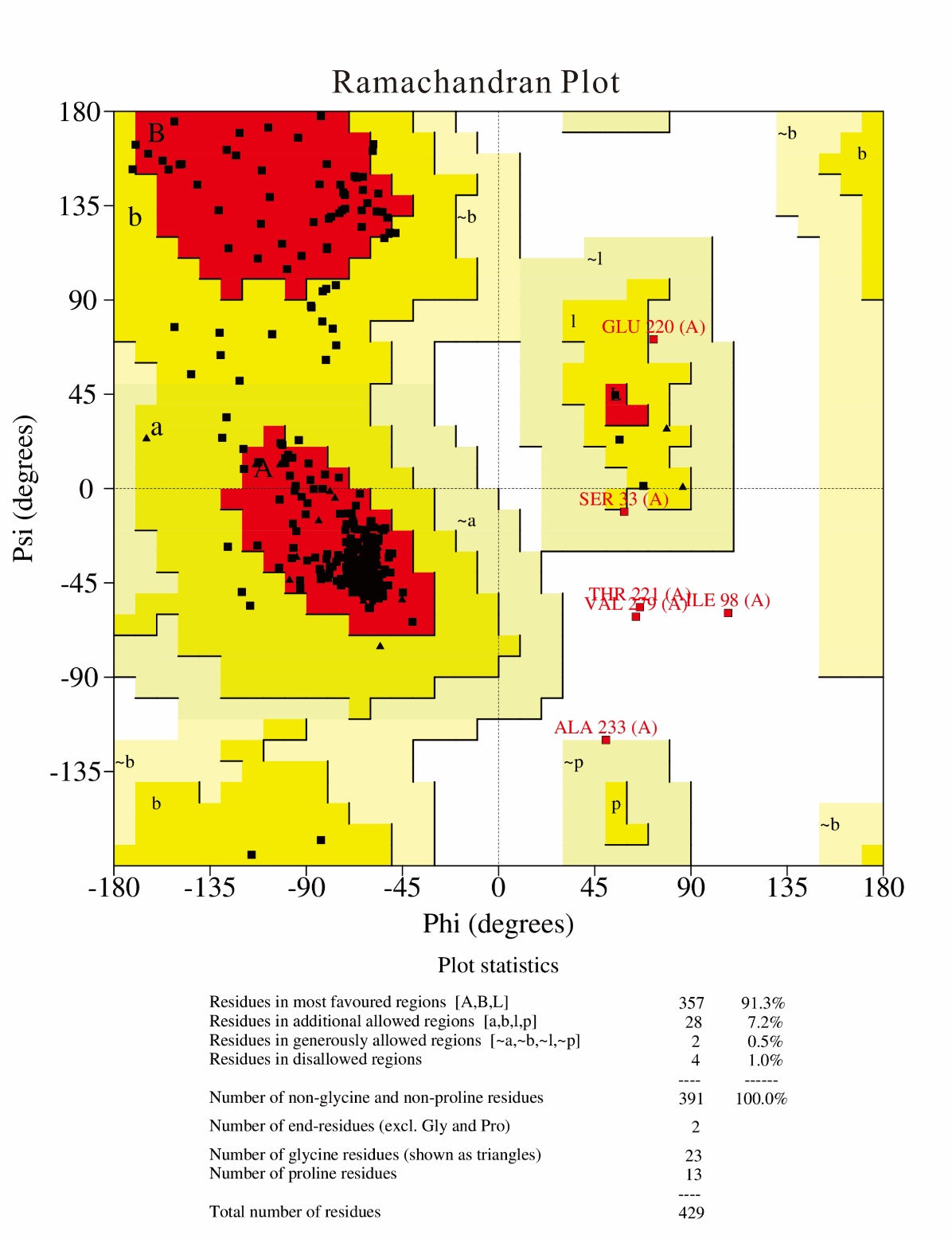


Figure S8. PROCHECK-Ramachandran plot of the ALKR predicted model. The most favored, additional allowed, generously allowed, and disallowed regions are colored in red, yellow, light yellow, and white respectively. Every dot (in squares or triangles) represents a single residue in the ALKR model, and most of the dots fall into red or yellow area, indicating the model is appropriate.


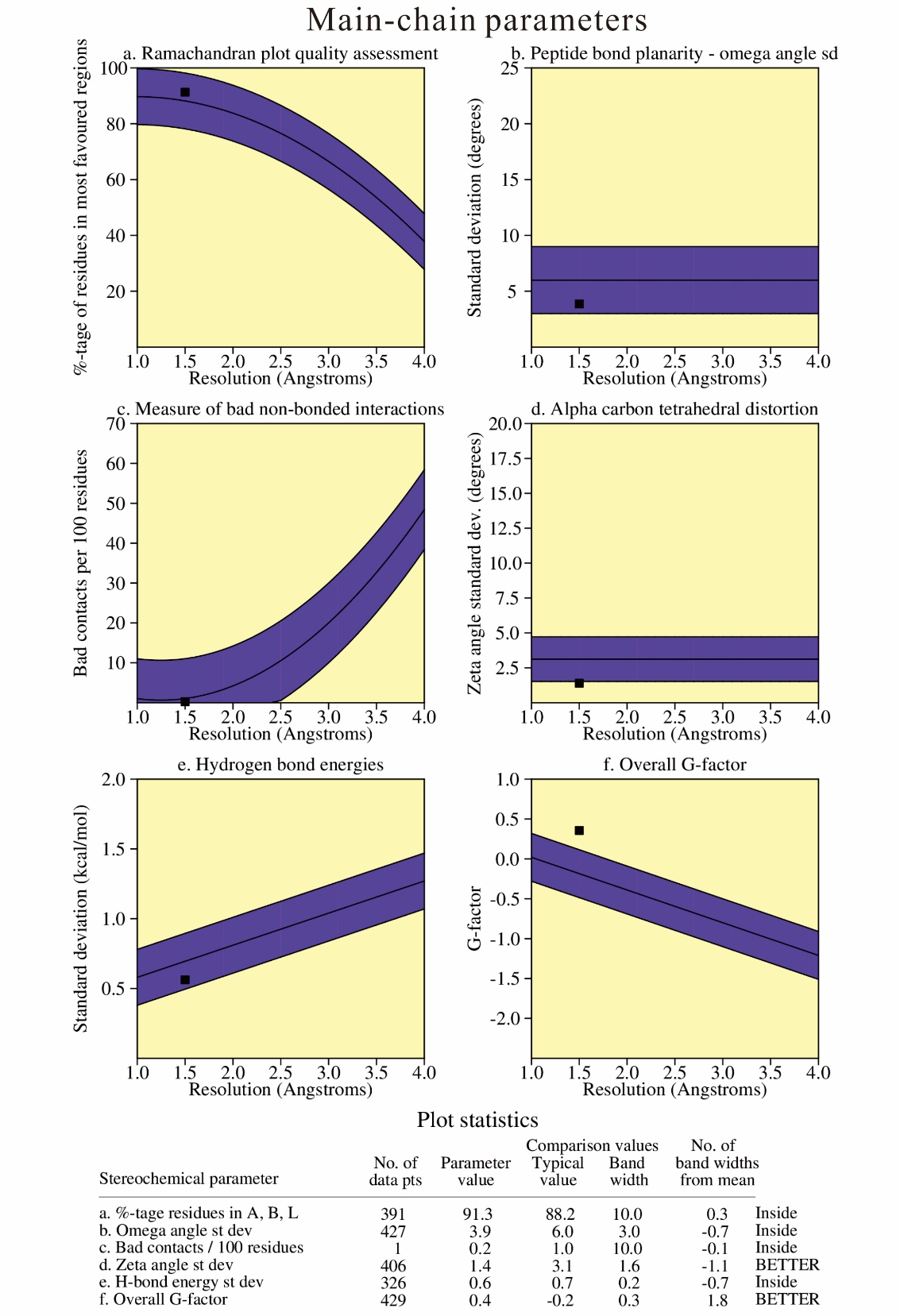


Figure S9. The main-chain parameters of ALKR modeled structure. The plots are generated by PROCHECK v.3.5.4. The small squares represent the ALKR model, and most of the squares are within the better regions or inside parameter regions, supporting that the model is appropriate.


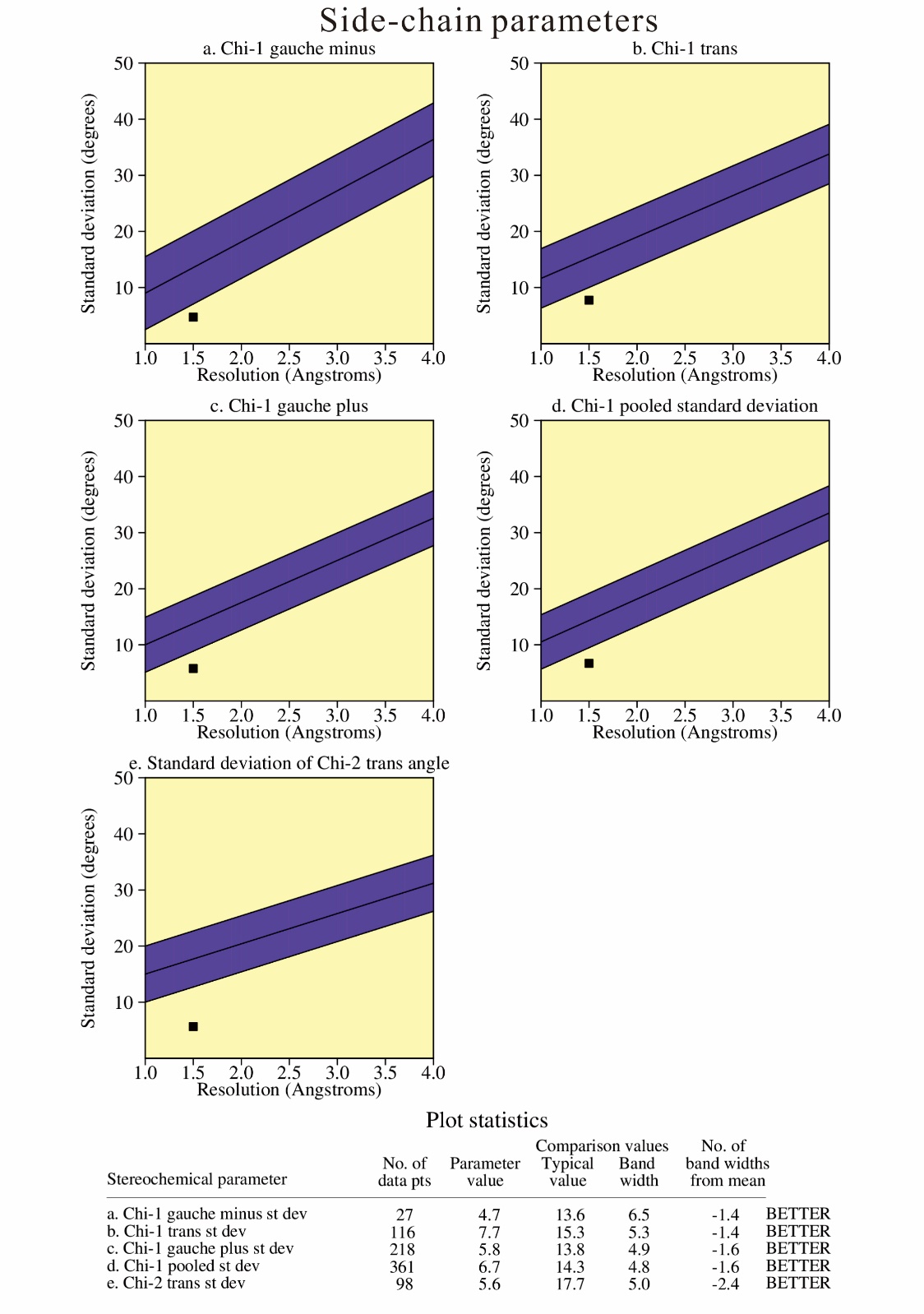


Figure S10. The side-chain parameters of ALKR modeled structure. The plots are generated by PROCHECK v.3.5.4. The small squares represent the ALKR model, and all of the squares are within the better regions, supporting that the model is appropriate.


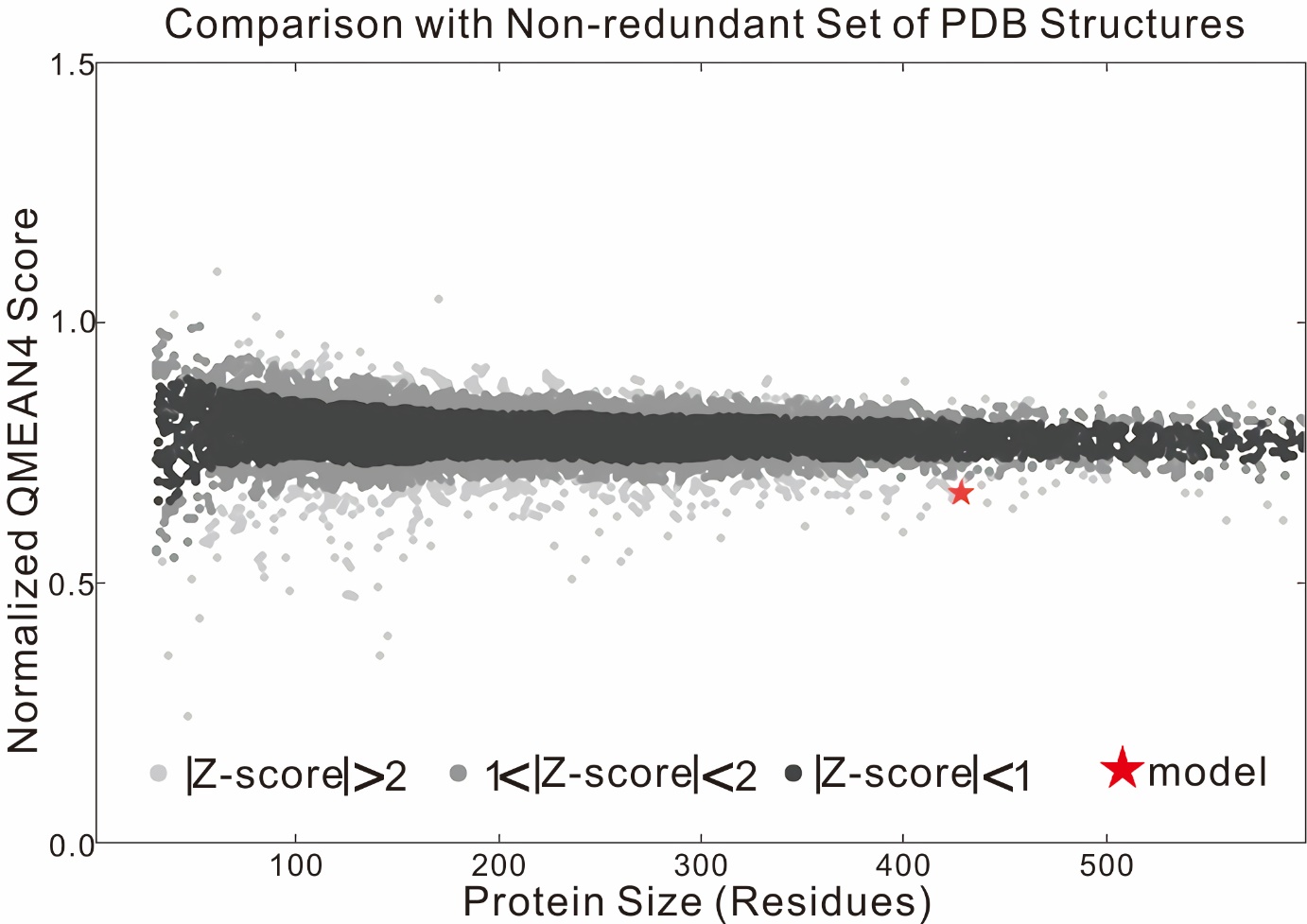


Figure S11. Comparison plot indicates the quality of the model in comparison with experimental structures of other proteins with similar sizes. The x-axis shows the protein length. The y-axis is the normalized QMEAN score. The ALKR predicted model is represented as a red star.


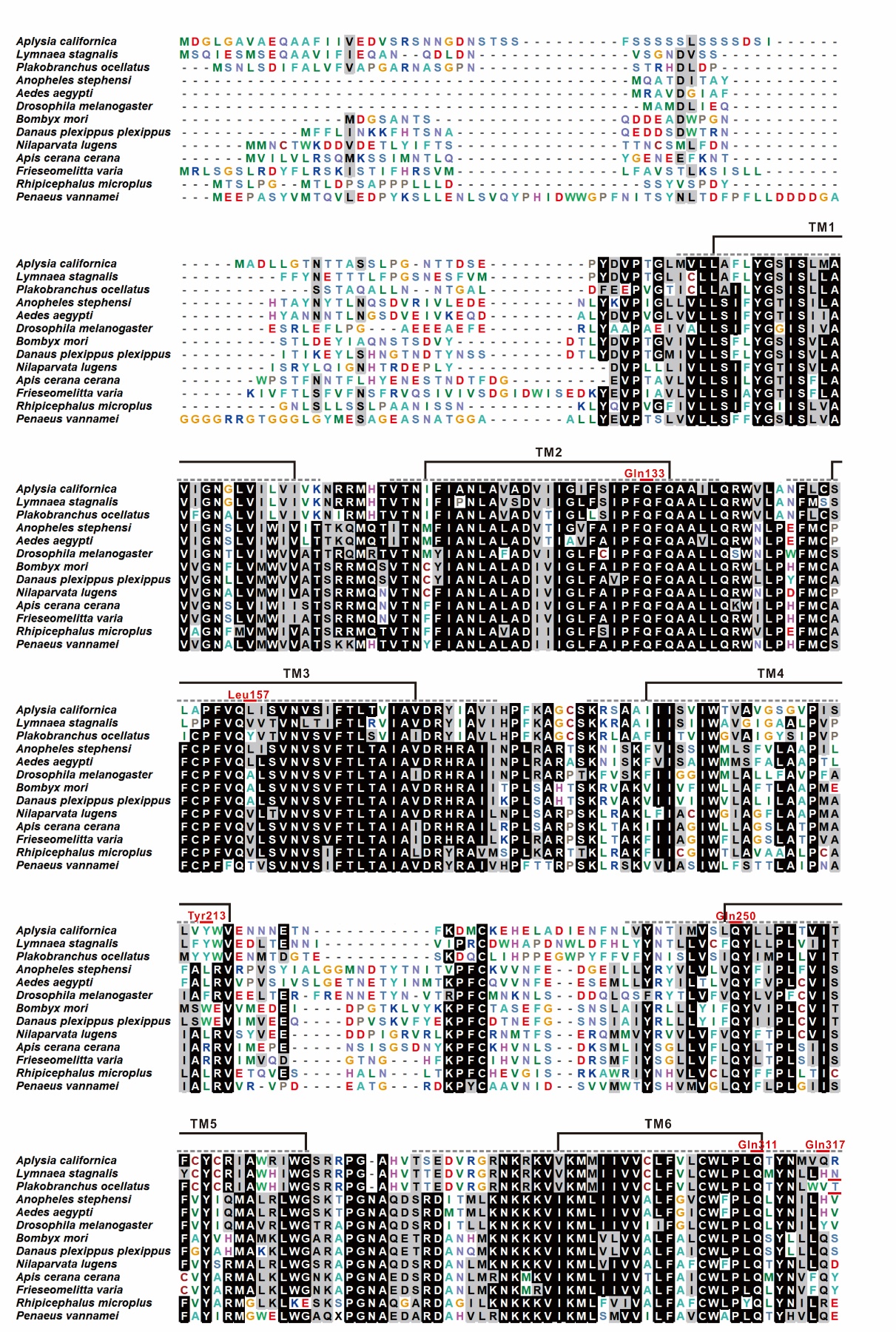


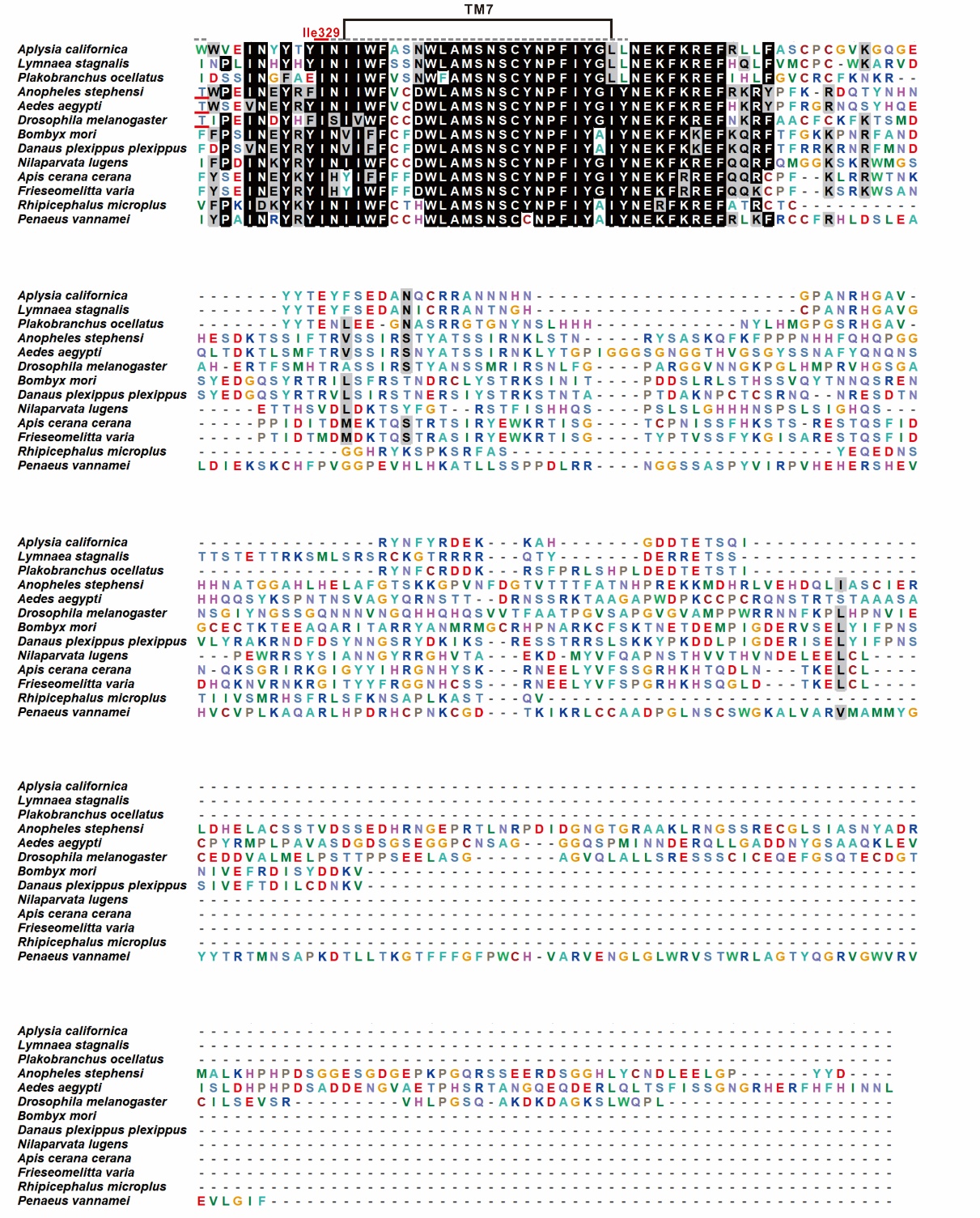


Figure S12. Comparison of ALKR with LKR sequences in other species illustrating the possibly conserved residues that may be interacting with LKs. The alignment is performed using BioEdit v5.0.6 (Clustal W Multiple alignment - Graphic View). The seven amino acids that are predicted to interact with active ALK peptides based on the docking model are marked in red on top of the ALKR.


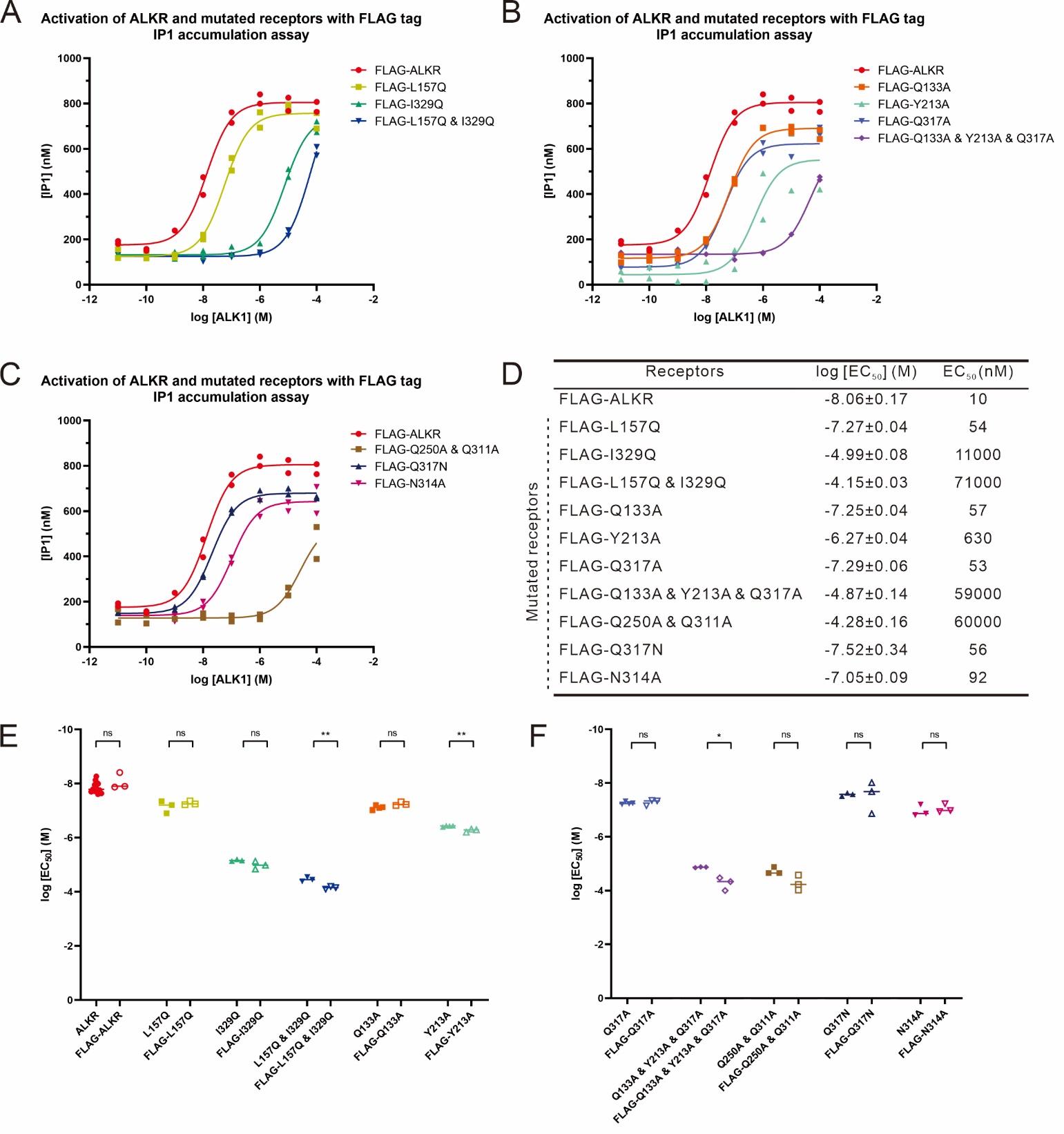


Figure S13. Mutagenesis of specific residues in the ALKR and mutant receptors containing FLAG tags at the N-termini. These residues are predicted to be involved in hydrophobic interactions (L157 and I329), H-bonds (Q133, Y213 and Q317) or amide-pi stackings (Q250 and Q311), or are suspected to interact with the ligand (N314). *A-C,* Dose-response curves showing the ability of ALK1 to activate the ALKR and the ALKR mutants with FLAG tags expressed in CHO-K1 cells, as determined by IP1 accumulation assay. *D,* Summary of the results shown in *A-C*: *n* = 3 for all experiments. Panels *A-D* correspond to panels *A-D* in Fig. 8, respectively, where experiments were carried out with receptors without FLAG tags. Note that the pattern of changes with receptors with FLAG tags shown in *D* is similar to those shown Fig. 8*D* for receptors without FLAG tags. *E* and *F,* Comparison of log [EC_50_] for the activation the receptor by ALK1 between the one without FLAG tag and the one with FLAG tag. Paired T-test: ns, *p* > 0.05; *, *p* < 0.05; **, *p* < 0.01.


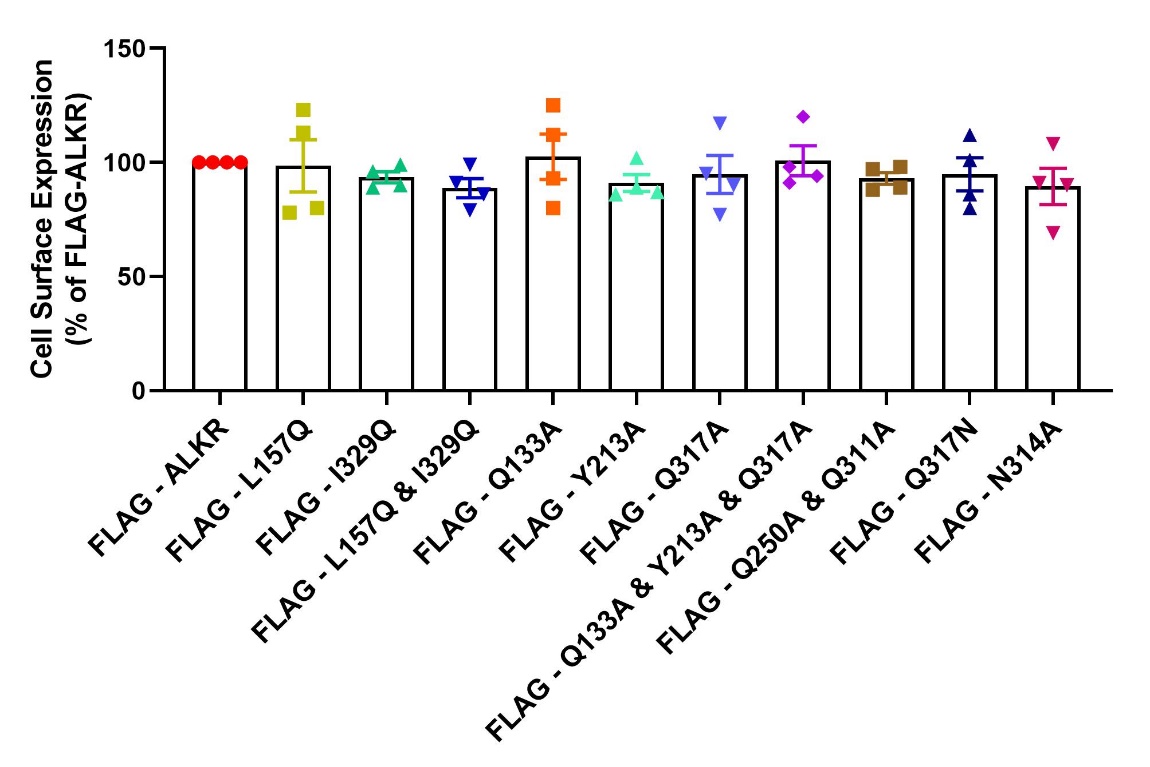


Figure S14. Cell surface expression of ALKR and 10 mutant receptors with FLAG tag. There were no significant changes for the expression of mutant receptors compared with that of the ALKR (*F* (10, 33) = 0.476, *p* = 0.8936).

Supporting references

1. Gupta, R., and Brunak, S. (2002) Prediction of glycosylation across the human proteome and the correlation to protein function. *Pac Symp Biocomput*, 310-322

2. Blom, N., Gammeltoft, S., and Brunak, S. (1999) Sequence and structure-based prediction of eukaryotic protein phosphorylation sites. *J Mol Biol* **294**, 1351-1362

3. Baek, M., DiMaio, F., Anishchenko, I., Dauparas, J., Ovchinnikov, S., Lee, G. R., Wang, J., Cong, Q., Kinch, L. N., Schaeffer, R. D., Millan, C., Park, H., Adams, C., Glassman, C. R., DeGiovanni, A., Pereira, J. H., Rodrigues, A. V., van Dijk, A. A., Ebrecht, A. C., Opperman, D. J., Sagmeister, T., Buhlheller, C., Pavkov-Keller, T., Rathinaswamy, M. K., Dalwadi, U., Yip, C. K., Burke, J. E., Garcia, K. C., Grishin, N. V., Adams, P. D., Read, R. J., and Baker, D. (2021) Accurate prediction of protein structures and interactions using a three-track neural network. *Science* **373**, 871-876

4. Laskowski, R. A., Rullmannn, J. A., MacArthur, M. W., Kaptein, R., and Thornton, J. M. (1996) AQUA and PROCHECK-NMR: programs for checking the quality of protein structures solved by NMR. *J Biomol NMR* **8**, 477-486

5. Benkert, P., Biasini, M., and Schwede, T. (2011) Toward the estimation of the absolute quality of individual protein structure models. *Bioinformatics* **27**, 343-350

6. Ujiantari, N. S. O., Ham, S., Nagiri, C., Shihoya, W., Nureki, O., Hutchinson, D. S., and Schuster, D. (2022) Pharmacophore-guided virtual screening to identify new β3-adrenergic receptor agonists. *Molecular Informatics* **41**, e2100223

7. Nagarajan, S. K., Babu, S., Devaraju, P., Sohn, H., and Madhavan, T. (2022) Structure and dynamics of the somatostatin receptor 3-ligand binding in the presence of lipids examined using computational structural biology methods. *Proteins: Structure, Function, and Bioinformatics* **90**, 704-719

8. Borovsky, D., Deckers, K., Vanhove, A. C., Verstraete, M., Rougé, P., Shatters, R. G., and Powell, C. A. (2021) Cloning and characterization of *Aedes aegypti* trypsin modulating oostatic factor (TMOF) gut receptor. *Biomolecules* **11**, 934

9. González-Beltrán, M., and Gómez-Alegría, C. (2021) Molecular modeling and bioinformatics analysis of drug-receptor interactions in the system formed by glargine, its metabolite M1, the insulin receptor, and the IGF1 receptor. *Bioinform Biol Insig* **15**, 11779322211046403

10. Ferreira de Freitas, R., and Schapira, M. (2017) A systematic analysis of atomic protein-ligand interactions in the PDB. *Medchemcomm* **8**, 1970-1981

11. Krone, M. W., Travis, C. R., Lee, G. Y., Eckvahl, H. J., Houk, K. N., and Waters, M. L. (2020) More than pi-pi-pi stacking: Contribution of amide-pi and CH-pi interactions to crotonyllysine binding by the AF9 YEATS domain. *J Am Chem Soc* **142**, 17048-17056

12. Tikhonova, I. G., Gigoux, V., and Fourmy, D. (2019) Understanding Peptide Binding in Class A G Protein-Coupled Receptors. *Mol. Pharmacol.* **96**, 550-561

13. Vu, O., Bender, B. J., Pankewitz, L., Huster, D., Beck-Sickinger, A. G., and Meiler, J. (2021) The structural basis of peptide binding at class A G protein-coupled receptors. *Molecules* **27**, 210
