# Supplementary material for: AI protein structure prediction-based modeling and mutagenesis of a protostome receptor and peptide ligands reveal key residues for their interaction
Source: J Biol Chem. 2022 Aug 30;298(10):102440. doi: 10.1016/j.jbc.2022.102440 (PMC9562341; doi:10.1016/j.jbc.2022.102440)

Contents

ALK1……………………………………………………………………………..1

ALK2……………………………………………………………………………..4

ALK3……………………………………………………………………………..7

ALK4……………………………………………………………………………10

ALK5……………………………………………………………………………13

ALK6……………………………………………………………………………16

ALK7……………………………………………………………………………19

ALK8……………………………………………………………………………22

ALK9……………………………………………………………………………25

ALK10…………………………………………………………………………..28

ALK11…………………………………………………………………………..31

ALK12…………………………………………………………………………..34

ALK13…………………………………………………………………………..37

ALK1_A1………………………………………………………………………40

ALK1_A3………………………………………………………………………43

ALK1_A4………………………………………………………………………46

ALK1_A6………………………………………………………………………49

ALK1_A7………………………………………………………………………52

ALK1-OH………………………………………………………………………55

Anopheles LK1……………………………………………………………….58

Anopheles LKR DNA sequence………………………………………………...61

ATRP……………………………………………………………………………64

ALK1

安徽省国平药业有限公司

CERTIFICATE OF ANALYSIS

**Order ID**	GP120371-1
**Name**	ALK1
**Lot No.**	GP120371-1-0216
**Sequence**	PAFHSWS[amide]
**Dissolution condition**	100%H2O
**Length**	7AA
**Modification**	N/A
**Molecular Weight (MW)**	829.89
**Storage**	-20°C

**Test Items**	**Specifications**	**Results**
**MW by MS**	829.60	Conforms
**Purity by HPLC**	>95%	97.501%
**Peptide Content**	N/A	N/A
**Moisture content**	N/A	N/A
**Acetic acid content**	N/A	N/A
**Appearance**	White to off-white lyophilized powder	Conforms
**Quantity**	10mg	2.0mg*5

Certified by: LiuHui Date 02/28/2020

Quality Assurance Department

Note: this product is intended for research use only; not for diagnostic or human use.

Guoping Pharmaceutical Co., LTD

地址:合肥市经开区桃花工业园拓展区工投立恒工业广场A2西F1,电话:0551-62841987 传真:0551-62841765 www.guopingyaoye.com

ALK1

Sample Information

Order ID :GP120371-1
Name :ALK1
Sequence :PAFHSWS[amide]
Lot.No :GP120371-1-0216
Pump A :0.1%Trifluoroacetic in 100% water
Pump B :0.1%Trifluoroacetic in 100% acetonitrile
Total Flow :1ml/min
Wavelength :220nm
Analytical column type :SHIMADZU Inertsil ODS-SP(4.6*250mm*5um)
Dissolution method :100%H2O
Inj. Volume :15 uL

Time	Module	Action	Value
0.01	Pumps	B.Conc	1
20.00	Pumps	B.Conc	41
23.00	Pumps	B.Conc	100
38.00	Pumps	B.Conc	100
40.00	Pumps	B.Conc	1
50.00	Controller	Stop	

Chromatogram

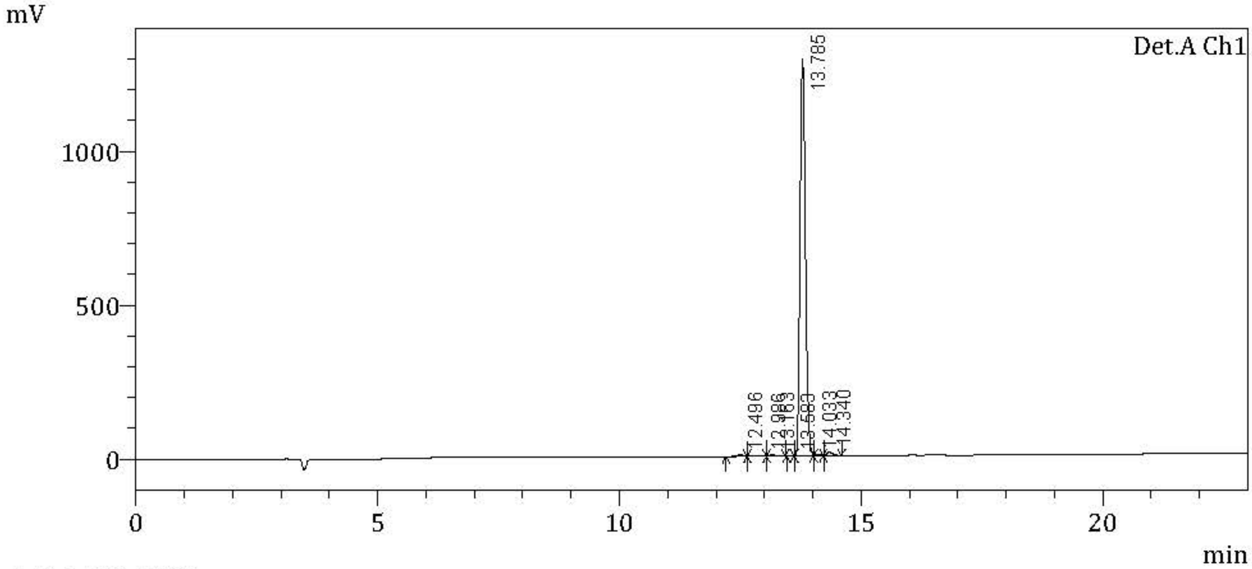

1 Det.A Ch1/220nm

PeakTable

Detector A Ch1 220nm

Peak#	Ret. Time	Area	Height	Area %	Height %
1	12.496	43547	4919	0.427	0.372
2	12.986	26738	2839	0.262	0.215
3	13.163	37407	3302	0.367	0.250
4	13.583	10222	1771	0.100	0.134
5	13.785	9945806	1291168	97.501	97.650
6	14.033	39453	6565	0.387	0.497
7	14.340	97562	11673	0.956	0.883
Total		10200736	1322236	100.000	100.000

ALK1

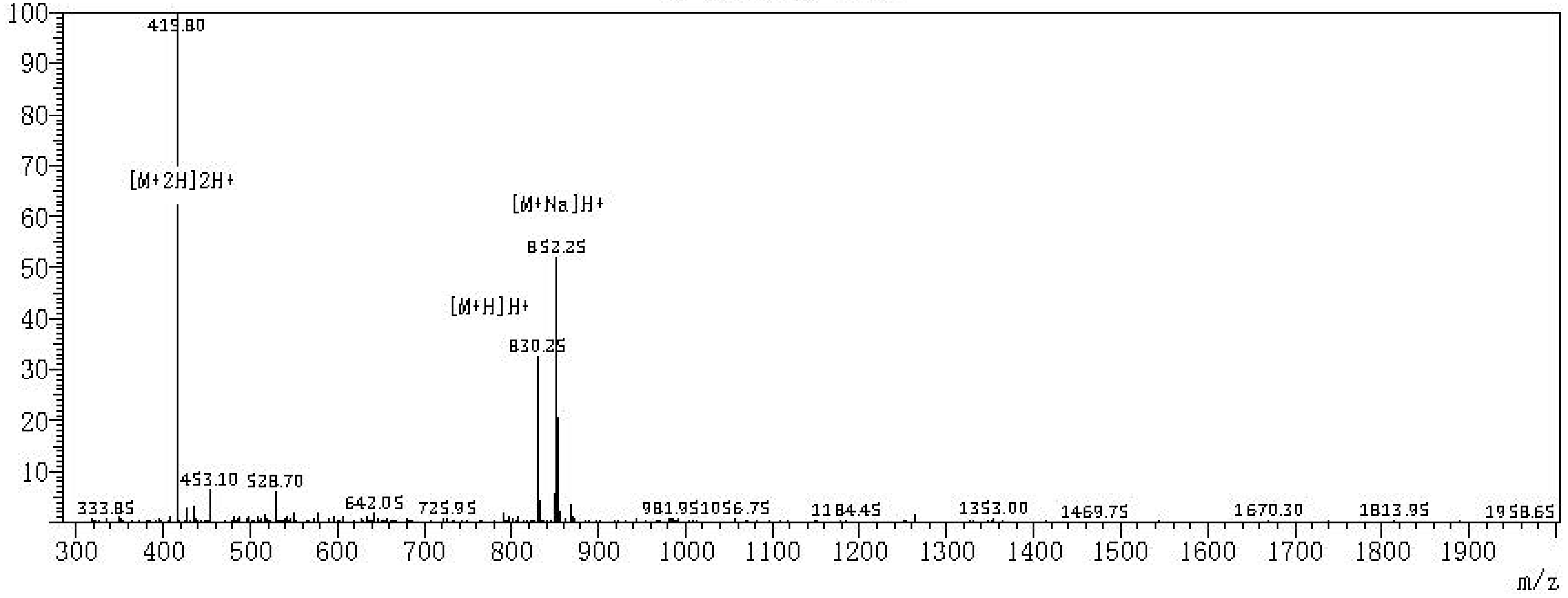

Sample Information

Dissolution method	:5%HAC+8%ACN+87%H2O	Interface	:ESI	Prerod Bias	:+1.5kv
Modified Date	:2020/02/26	Nebulizing Gas Flow	:1.50L/min	Detector	:-0.2kv
Injection Volume	:1ul	CDL Temp	:250C	T.Flow	:0.2ml/min
Heat Block Temp	:200	CDL Volt	:0v	B.conc	:50%H2O/50%MEOH

Order ID	:GP120371-1
Name	:ALK1
Sequence	:PAFHSWS[amide]
Lot.No	:GP120371-1-0216
Theoretical	:829.89
Observed	:829.60

安徽省国平药业有限公司

CERTIFICATE OF ANALYSIS

**Order ID**	GP120371-2
**Name**	ALK2
**Lot No.**	GP120371-2-0216
**Sequence**	PAFHAWS[amide]
**Dissolution condition**	100%H2O
**Length**	7AA
**Modification**	N/A
**Molecular Weight (MW)**	813.89
**Storage**	-20°C

**Test Items**	**Specifications**	**Results**
**MW by MS**	813.60	Conforms
**Purity by HPLC**	>95%	96.753%
**Peptide Content**	N/A	N/A
**Moisture content**	N/A	N/A
**Acetic acid content**	N/A	N/A
**Appearance**	White to off-white lyophilized powder	Conforms
**Quantity**	10mg	2.0mg*5

Certified by: LiuHui Date 02/28/2020

Quality Assurance Department

Note: this product is intended for research use only; not for diagnostic or human use.

Guoping Pharmaceutical Co., LTD

地址:合肥市经开区桃花工业园拓展区工投立恒工业广场A2西F1,电话:0551-62841987 传真:0551-62841765 www.guopingyaoye.com

Sample Information

Order ID :GP120371-2
Name :ALK2
Sequence :PAFHAWS[amide]
Lot.No :GP120371-2-0216
Pump A :0.1%Trifluoroacetic in 100% water
Pump B :0.1%Trifluoroacetic in 100% acetonitrile
Total Flow :1ml/min
Wavelength :220nm
Analytical column type :SHIMADZU Inertsil ODS-SP(4.6*250mm*5um)
Dissolution method :100%H2O
Inj. Volume :8 uL

Time	Module	Action	Value
0.01	Pumps	B.Conc	10
20.00	Pumps	B.Conc	50
23.00	Pumps	B.Conc	100
38.00	Pumps	B.Conc	100
40.00	Pumps	B.Conc	10
50.00	Controller	Stop	

Chromatogram

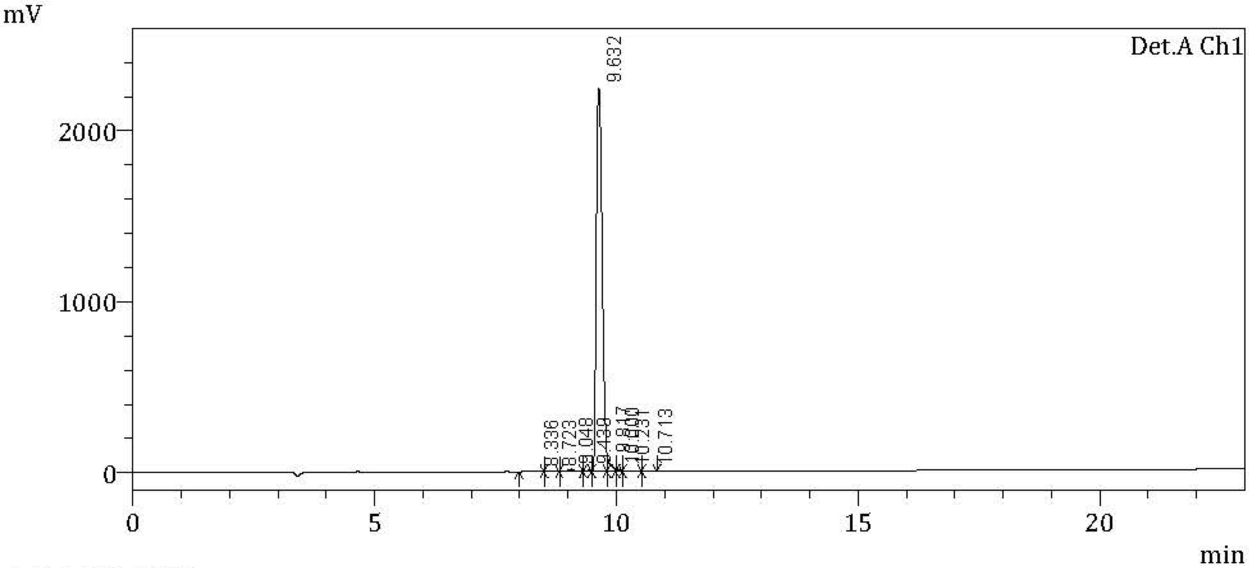

1 Det.A Ch1/220nm

PeakTable

Detector A Ch1 220nm

Peak#	Ret. Time	Area	Height	Area %	Height %
1	8.336	10682	985	0.055	0.042
2	8.723	10508	1083	0.054	0.046
3	9.048	139885	15448	0.723	0.655
4	9.439	49780	8206	0.257	0.348
5	9.632	18728195	2240636	96.753	95.065
6	9.817	333694	77820	1.724	3.302
7	10.000	45715	8958	0.236	0.380
8	10.231	34681	3539	0.179	0.150
9	10.713	3540	279	0.018	0.012
Total		19356680	2356955	100.000	100.000

ALK2

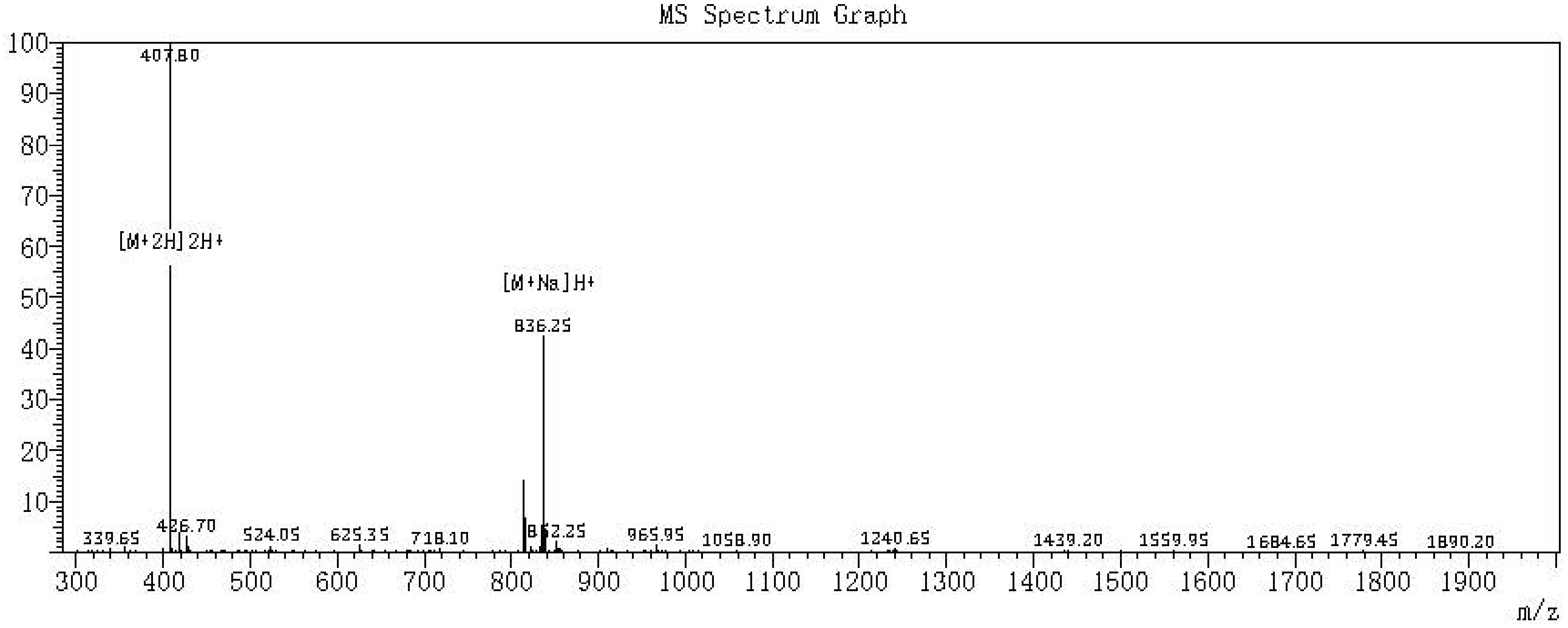

Sample Information

Dissolution method	:5%HAC+8%ACN+87%H2O	Interface	:ESI	Prerod Bias	:+1.5kv
Modified Date	:2020/02/28	Nebulizing Gas Flow	:1.50L/min	Detector	:-0.2kv
Injection Volume	:1ul	CDL Temp	:250C	T.Flow	:0.2ml/min
Heat Block Temp	:200	CDL Volt	:0v	B.conc	:50%H2O/50%MEOH

Order ID	:GP120371-2
Name	:ALK2
Sequence	:PAFHAWS[amide]
Lot.No	:GP120371-2-0216
Theoretical	:813.89
Observed	:813.60

CERTIFICATE OF ANALYSIS

**Product Name**	P505_S511
**Lot No**	JT-91974
**Sequence**	PAFSAWS-NH2
**Dissolution condition**	15%ACN+85%H2O
**Length**	7AA
**Modification**	N/A
**Molecular Weight (MW)**	763.82
**Storage**	-20℃

**Test Items**	**Specifications**	**Results**
**Purity by HPLC**	95%	98.94%
**Peptide Content**	N/A	N/A
**Moisture content**	N/A	N/A
**Acetic acid content**	N/A	N/A
**Appearance**	White to off-white lyophilized powder	Conforms
**Quantity**	2mg	2.0mg

Certified by:
Quality Assurance Department

Date 10-22-2020

Note: this product is intended for research use only; not for diagnostic or human use.

Synpeptide Co., Ltd

Address: 320 Lvlin Road, Beicai, Pudong new area, Shanghai, China 201204

Tel: +86-21-50835580 Fax: +86-21-50835580 P/C: 201204 http://www.synpeptide.com

ALK3

Sample Information

Order ID	: Syn-91974
Name	: P505_S511
Sequence	: PAFSAWS-NH2
Lot No	: JT-91974
Pump A	: 0.1% Trifluoroacetic in 100% Water
Pump B	: 0.1% Trifluoroacetic in 100% Acetonrtrile
Total Flow	: 1ml/min
Wavelength	: 220nm
Analytial column type	: SHIMADZU Inertsil ODS-SP（4.6*250mm*5um）
Inj. Volume	: 30ul

Time	Module	Action	Value
0.00	Pumps	B.Conc	10
25.00	Pumps	B.Conc	70
25.01	Pumps	B.Conc	100
30.00	Pumps	B.Conc	100
30.01	Pumps	Stop	

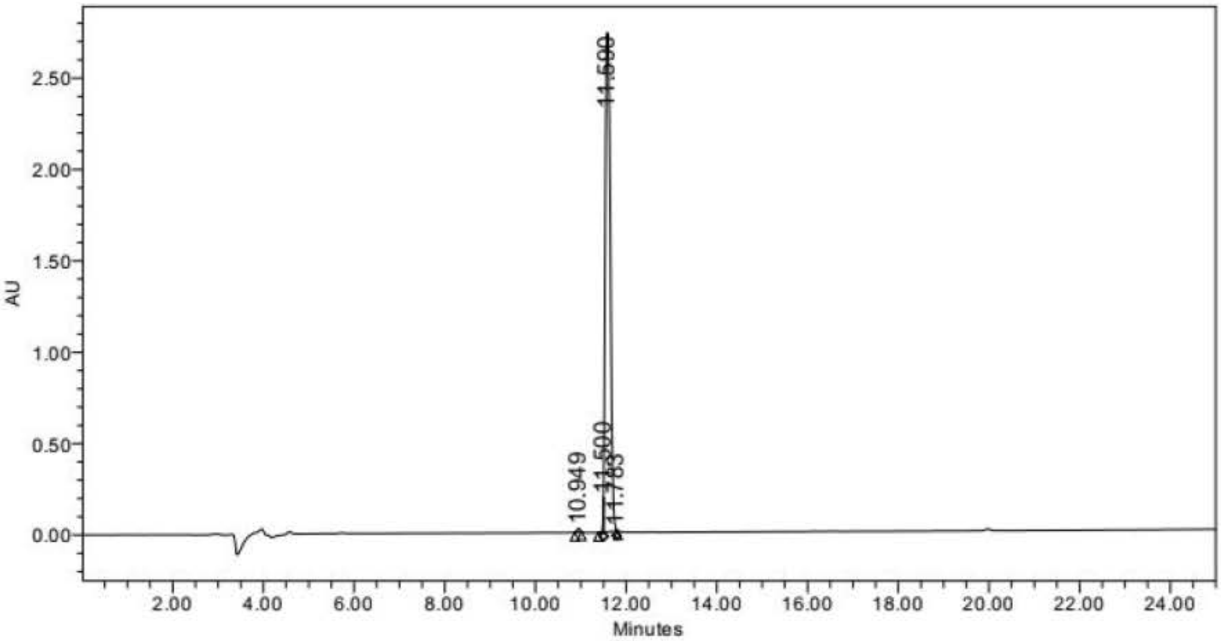

	RT	Area	% Area	Height
1	10.949	88528	0.41	18640
2	11.500	133747	0.62	186938
3	11.590	21402927	98.94	2760822
4	11.783	6999	0.03	8345

Synpeptide Co., Ltd

Address: 320 Lvlin Road, Beicai, Pudong new area, Shanghai, China 201204

Tel: +86-21-50835580 Fax: +86-21-50835580 P/C: 201204 http://www.synpeptide.com

ALK3

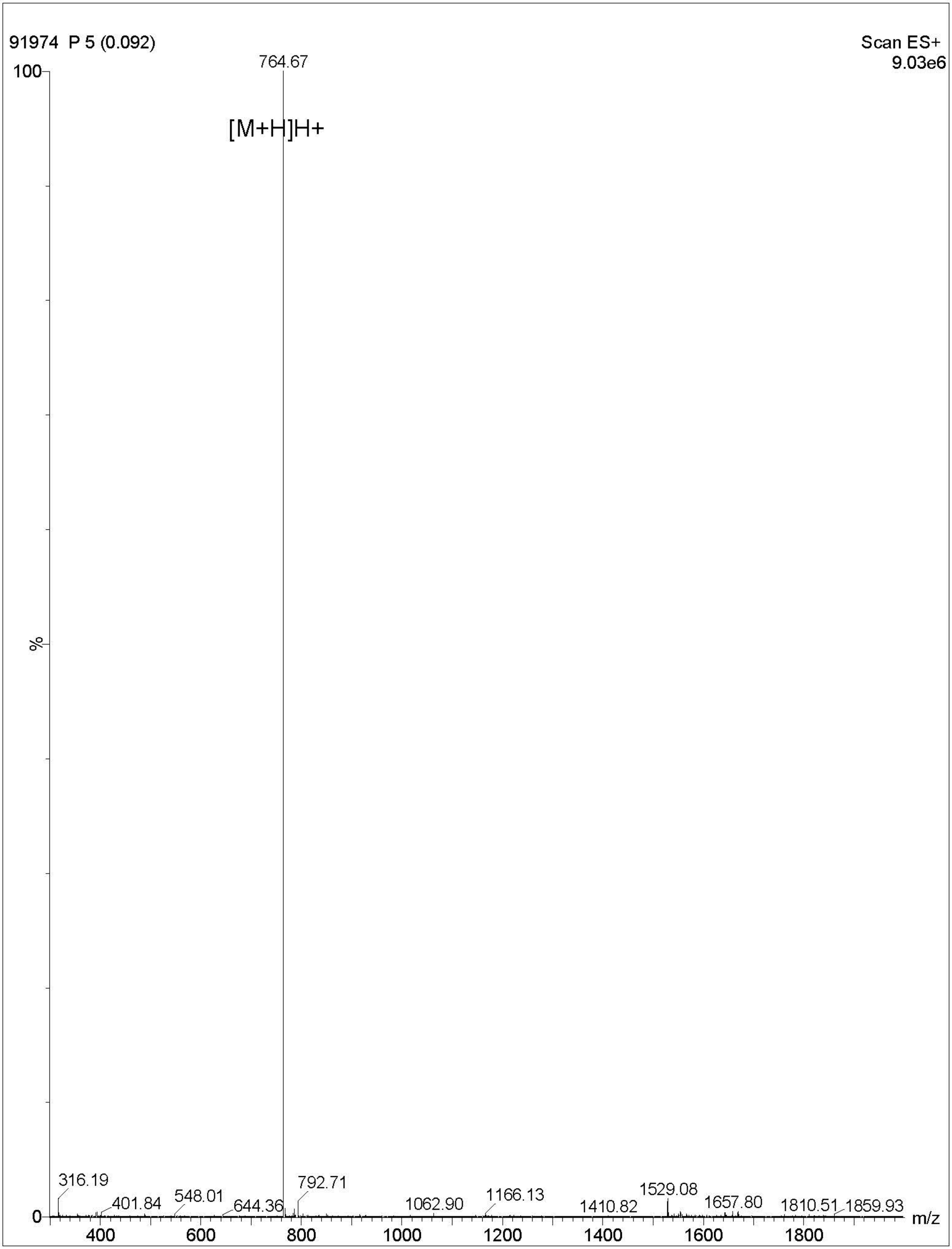

91974 P 5 (0.092)
Scan ES+
9.03e6
100
764.67
[M+H]H+
%
316.19
401.84
548.01
644.36
792.71
1062.90
1166.13
1410.82
1529.08
1657.80
1810.51
1859.93
0
400
600
800
1000
1200
1400
1600
1800
m/z

ALK4

安徽省国平药业有限公司

CERTIFICATE OF ANALYSIS

**Order ID**	GP120424-5
**Name**	A171_A177
**Lot No.**	GP120424-5-0413
**Sequence**	AGFAPWA-NH2
**Dissolution condition**	100%H2O
**Length**	7AA
**Modification**	N/A
**Molecular Weight (MW)**	717.80
**Storage**	-20°C

**Test Items**	**Specifications**	**Results**
**MW by MS**	717.20	Conforms
**Purity by HPLC**	>95%	98.760%
**Peptide Content**	N/A	N/A
**Moisture content**	N/A	N/A
**Acetic acid content**	N/A	N/A
**Appearance**	White to off-white lyophilized powder	Conforms
**Quantity**	2mg	2.0mg

Certified by: LiuHui   Date 04/30/2021

Quality Assurance Department

Note: this product is intended for research use only; not for diagnostic or human use.

Guoping Pharmaceutical Co., LTD

地址:合肥市经开区桃花工业园拓展区工投立恒工业广场A2西F1,电话:0551-62841987 传真:0551-62841765 www.guopingyaoye.com

Sample Information

Order ID :GP120424-5
Name :A171_A177
Sequence :AGFAPWA-NH2
Lot.No :GP120424-5-0413
Pump A :0.1%Trifluoroacetic in 100% water
Pump B :0.1%Trifluoroacetic in 100% acetonitrile
Total Flow :1ml/min
Wavelength :220nm
Analytical column type :SHIMADZU Inertsil ODS-SP(4.6*250mm*5um)
Dissolution method :100%H2O
Inj. Volume :9 uL

Time	Module	Action	Value
0.01	Pumps	B.Conc	15
20.00	Pumps	B.Conc	55
30.00	Pumps	B.Conc	100
38.00	Pumps	B.Conc	100
40.00	Pumps	B.Conc	15
50.00	Controller	Stop	

Chromatogram

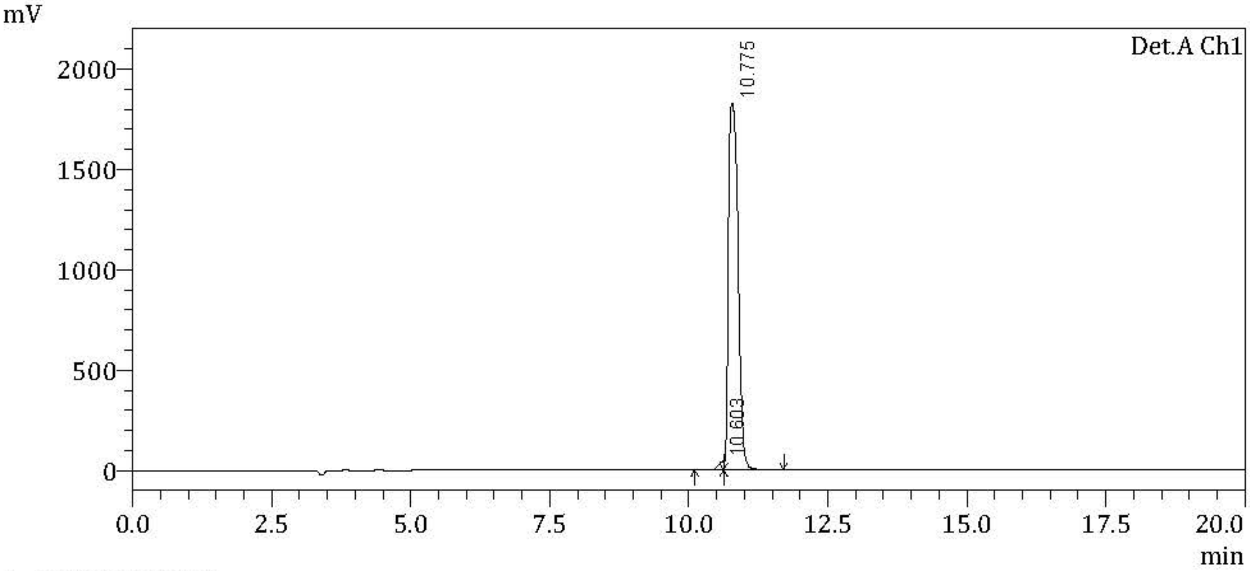

1 Det.A Ch1/220nm

PeakTable

Detector A Ch1 220nm

Peak#	Ret. Time	Area	Height	Area %	Height %
1	10.603	274752	41658	1.240	2.234
2	10.775	21881297	1823400	98.760	97.766
Total		22156050	1865058	100.000	100.000

ALK4

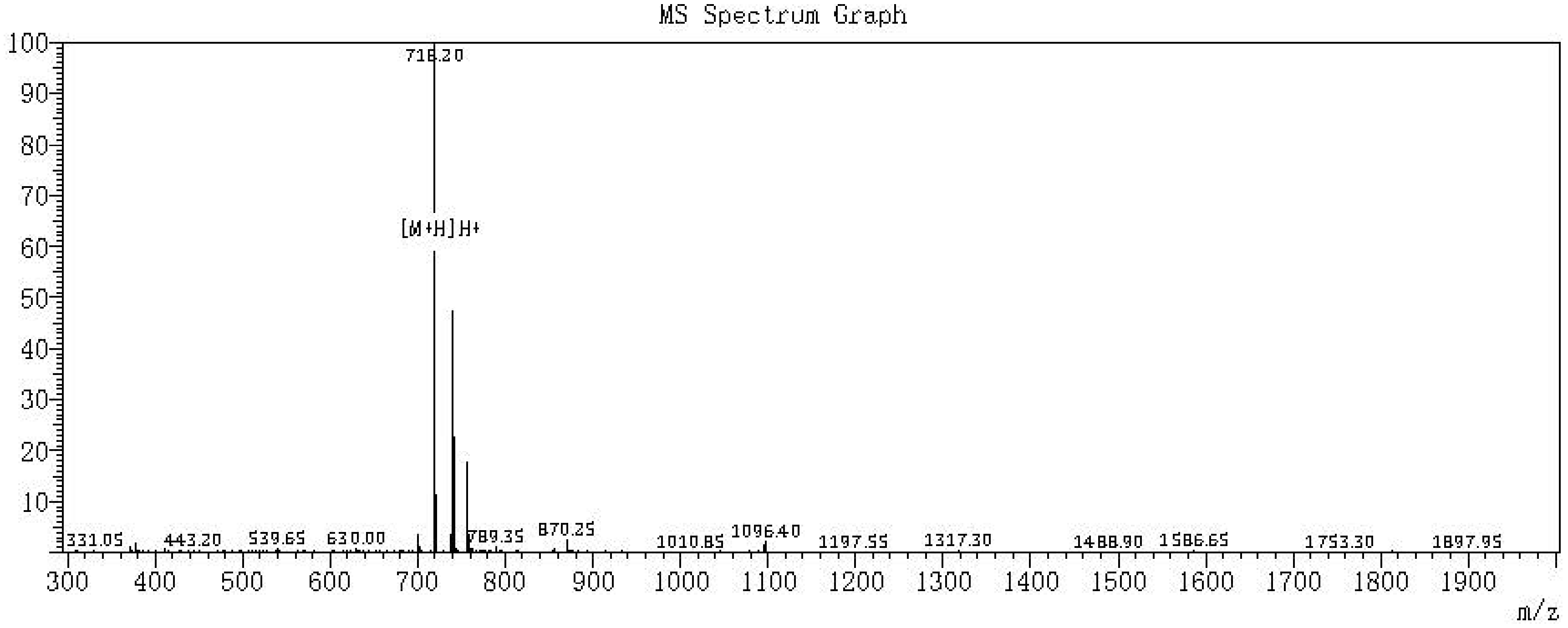

Sample Information

Dissolution method	:5%HAC+8%ACN+87%H2O	Interface	:ESI	Prerod Bias	:+1.5kv
Modified Date	:2021/04/30	Nebulizing Gas Flow	:1.50L/min	Detector	:-0.2kv
Injection Volume	:1ul	CDL Temp	:250C	T.Flow	:0.2ml/min
Heat Block Temp	:200	CDL Volt	:0v	B.conc	:50%H2O/50%MEOH

Order ID	:GP120424-5
Name	:A171_A177
Sequence	:AGFAPWA-NH2
Lot.No	:GP120424-5-0413
Theoretical	:717.80
Observed	:717.20

CERTIFICATE OF ANALYSIS

**Product Name**	P246_N252
**Lot No**	JT-91976
**Sequence**	PAFSAWN-NH2
**Dissolution condition**	15%ACN+85%H2O
**Length**	7AA
**Modification**	N/A
**Molecular Weight (MW)**	790.85
**Storage**	-20℃

**Test Items**	**Specifications**	**Results**
**Purity by HPLC**	95%	95.06%
**Peptide Content**	N/A	N/A
**Moisture content**	N/A	N/A
**Acetic acid content**	N/A	N/A
**Appearance**	White to off-white lyophilized powder	Conforms
**Quantity**	2mg	2.0mg

Certified by:
Quality Assurance Department

Date 10-26-2020

Note: this product is intended for research use only; not for diagnostic or human use.

Synpeptide Co., Ltd

Address: 320 Lvlin Road, Beicai, Pudong new area, Shanghai, China 201204

Tel: +86-21-50835580 Fax: +86-21-50835580 P/C: 201204 http://www.synpeptide.com

Sample Information

Order ID : Syn-91976
Name : P246_N252
Sequence : PAFSAWN-NH2
Lot No : JT-91976
Pump A : 0.1% Trifluoroacetic in 100% Water
Pump B : 0.1% Trifluoroacetic in 100% Acetonrtrile
Total Flow : 1ml/min
Wavelength : 220nm
Analytial column type : SHIMADZU Inertsil ODS-SP（4.6*250mm*5um）
Inj. Volume : 30ul

Time	Module	Action	Value
0.00	Pumps	B.Conc	10
25.00	Pumps	B.Conc	70
25.01	Pumps	B.Conc	100
30.00	Pumps	B.Conc	100
30.01	Pumps	Stop	

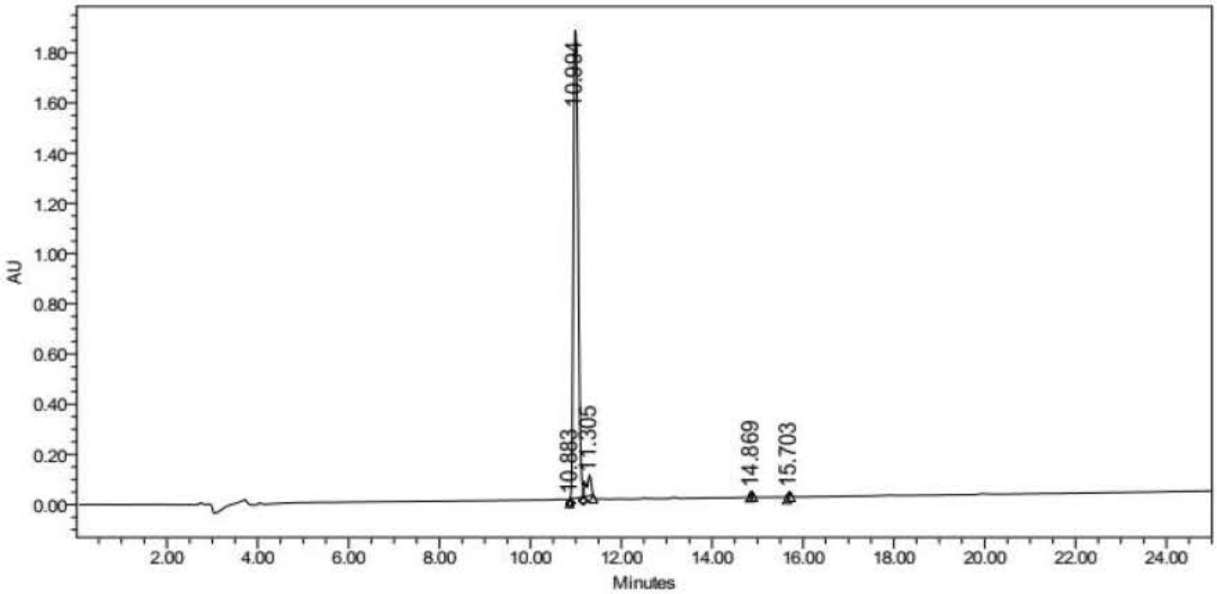

	RT	Area	% Area	Height
1	10.883	1737	0.01	3473
2	10.994	13499542	95.06	1904849
3	11.305	644460	4.54	81197
4	14.869	24513	0.17	9838
5	15.703	30491	0.21	9919

Synpeptide Co., Ltd

Address: 320 Lvlin Road, Beicai, Pudong new area, Shanghai, China 201204
Tel: +86-21-50835580 Fax: +86-21-50835580 P/C: 201204 http://www.synpeptide.com

ALK5

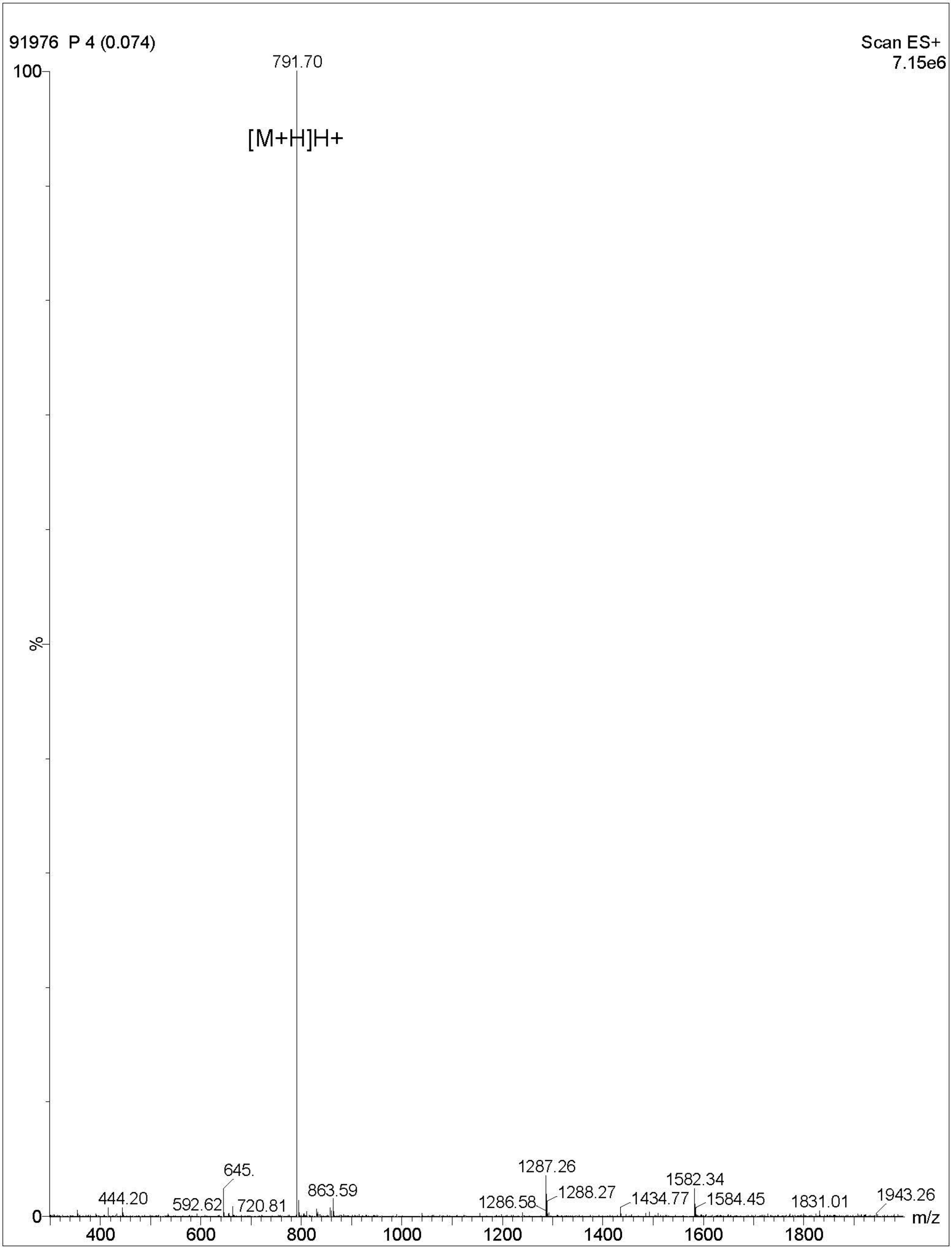

91976 P 4 (0.074)
Scan ES+
7.15e6
100
%
0
791.70
[M+H]H+
645.
444.20
592.62
720.81
863.59
1287.26
1286.58
1288.27
1434.77
1582.34
1584.45
1831.01
1943.26
400
600
800
1000
1200
1400
1600
1800
m/z

ALK6

CERTIFICATE OF ANALYSIS

**Product Name**	S326_S336
**Lot No**	JT-91978
**Sequence**	SEKQPAFHAWS-NH2
**Dissolution condition**	15%ACN+85%H2O
**Length**	11AA
**Modification**	N/A
**Molecular Weight (MW)**	1286.39
**Storage**	-20℃

**Test Items**	**Specifications**	**Results**
**Purity by HPLC**	95%	98.58%
**Peptide Content**	N/A	N/A
**Moisture content**	N/A	N/A
**Acetic acid content**	N/A	N/A
**Appearance**	White to off-white lyophilized powder	Conforms
**Quantity**	2mg	2.0mg

Certified by:
Quality Assurance Department

Date 10-26-2020

Note: this product is intended for research use only; not for diagnostic or human use.

Synpeptide Co., Ltd

Address: 320 Lvlin Road, Beicai, Pudong new area, Shanghai, China 201204
Tel: +86-21-50835580 Fax: +86-21-50835580 P/C: 201204 http://www.synpeptide.com

ALK6

Sample Information

Order ID : Syn-91978
Name : S326_S336
Sequence : SEKQPAFHAWS-NH2
Lot No : JT-91978
Pump A : 0.1% Trifluoroacetic in 100% Water
Pump B : 0.1% Trifluoroacetic in 100% Acetonrtrile
Total Flow : 1ml/min
Wavelength : 220nm
Analytial column type : SHIMADZU Inertsil ODS-SP（4.6*250mm*5um）
Inj. Volume : 30ul

Time	Module	Action	Value
0.00	Pumps	B.Conc	10
25.00	Pumps	B.Conc	70
25.01	Pumps	B.Conc	100
30.00	Pumps	B.Conc	100
30.01	Pumps	Stop	

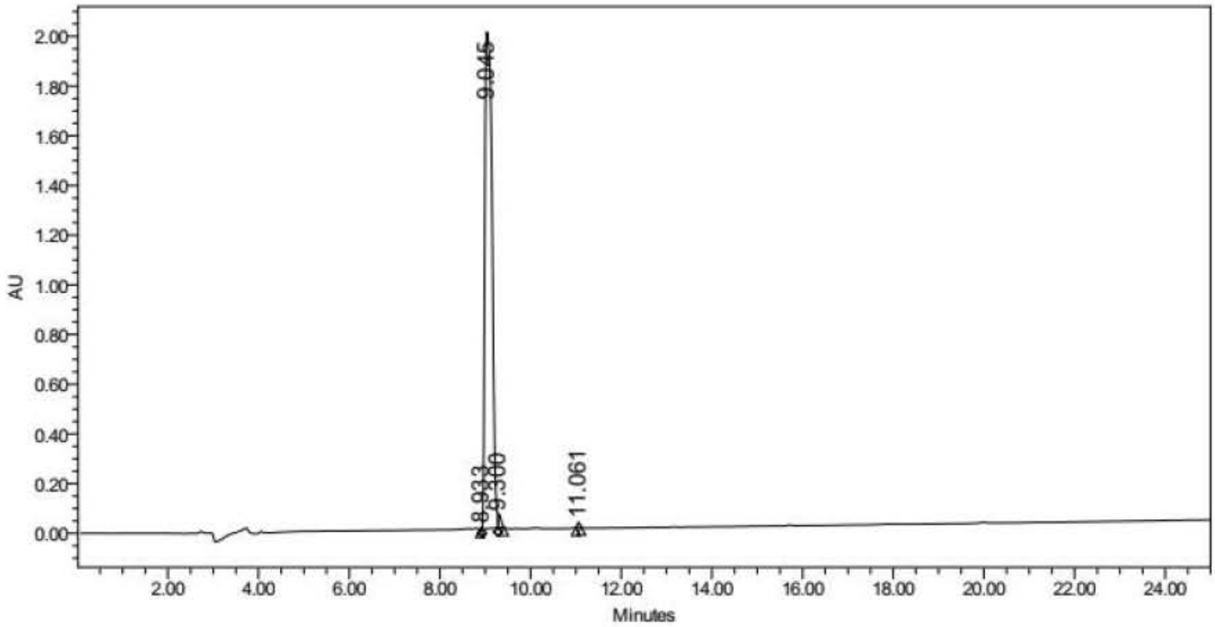

	RT	Area	% Area	Height
1	8.933	6957	0.03	7823
2	9.045	21943295	98.58	2037462
3	9.300	239689	1.08	53334
4	11.061	68836	0.31	17416

Synpeptide Co., Ltd

Address: 320 Lvlin Road, Beicai, Pudong new area, Shanghai, China 201204
Tel: +86-21-50835580 Fax: +86-21-50835580 P/C: 201204 http://www.synpeptide.com

ALK6

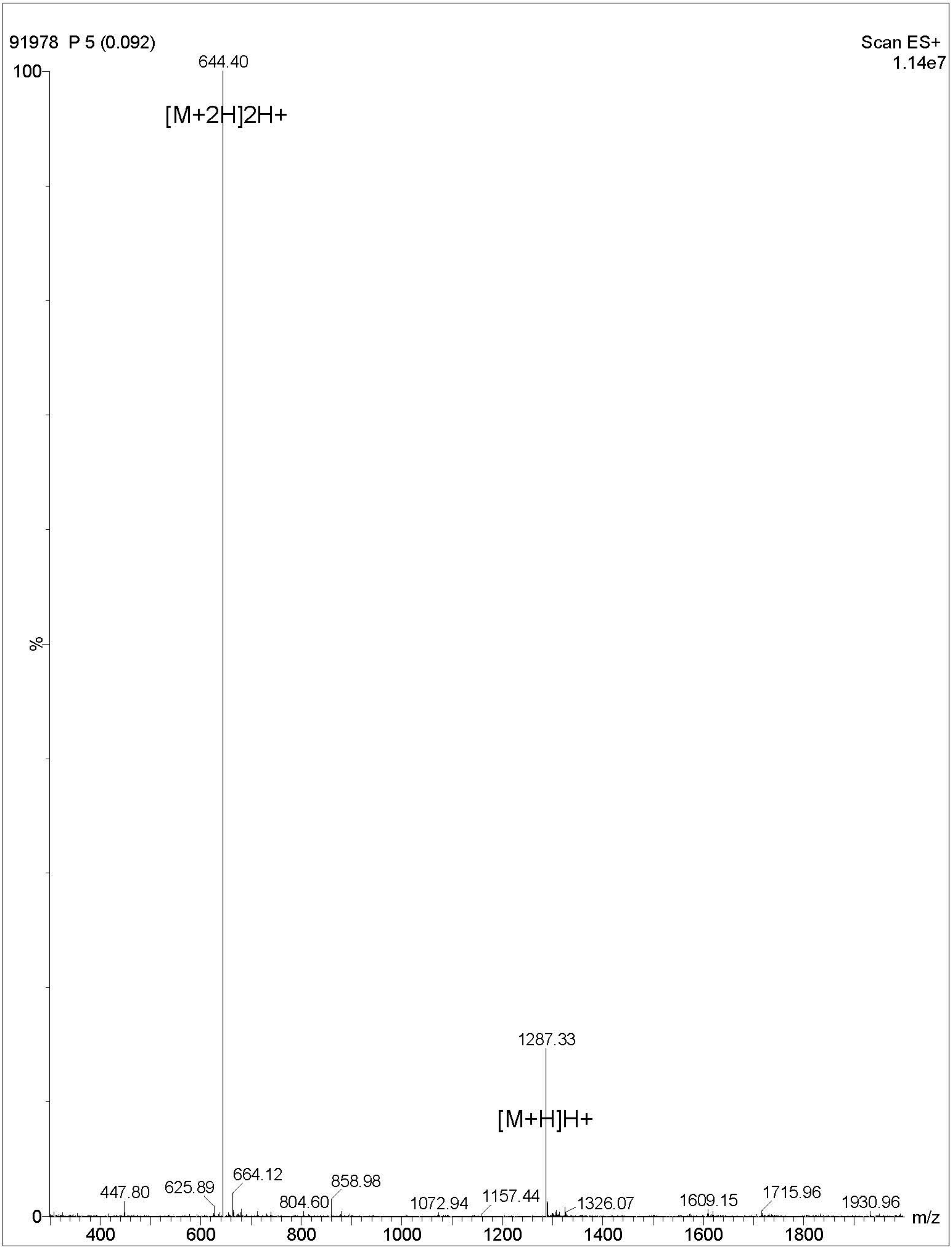

91978 P 5 (0.092)
Scan ES+
1.14e7
644.40
[M+2H]2H+
1287.33
[M+H]H+
447.80
625.89
664.12
804.60
858.98
1072.94
1157.44
1326.07
1609.15
1715.96
1930.96
100
%
0
400
600
800
1000
1200
1400
1600
1800
m/z

安徽省国平药业有限公司

CERTIFICATE OF ANALYSIS

**Order ID**	GP120424-2
**Name**	P468_S474
**Lot No.**	GP120424-2-0413
**Sequence**	PRFHAWS-NH2
**Dissolution condition**	100%H2O
**Length**	7AA
**Modification**	N/A
**Molecular Weight (MW)**	899.00
**Storage**	-20°C

**Test Items**	**Specifications**	**Results**
**MW by MS**	898.70	Conforms
**Purity by HPLC**	>95%	95.338%
**Peptide Content**	N/A	N/A
**Moisture content**	N/A	N/A
**Acetic acid content**	N/A	N/A
**Appearance**	White to off-white lyophilized powder	Conforms
**Quantity**	2mg	2.0mg

Certified by: LiuHui　　Date 04/30/2021

Quality Assurance Department

Note: this product is intended for research use only; not for diagnostic or human use.

Guoping Pharmaceutical Co., LTD

地址:合肥市经开区桃花工业园拓展区工投立恒工业广场A2西F1,电话:0551-62841987 传真:0551-62841765 www.guopingyaoye.com

ALK7

Sample Information

Order ID	:GP120424-2
Name	:P468_S474
Sequence	:PRFHAWS-NH2
Lot.No	:GP120424-2-0413
Pump A	:0.1%Trifluoroacetic in 100% water
Pump B	:0.1%Trifluoroacetic in 100% acetonitrile
Total Flow	:1ml/min
Wavelength	:220nm
Analytical column type	:SHIMADZU Inertsil ODS-SP(4.6*250mm*5um)
Dissolution method	:100%H2O
Inj. Volume	:14 uL

Time	Module	Action	Value
0.01	Pumps	B.Conc	15
20.00	Pumps	B.Conc	55
30.00	Pumps	B.Conc	100
38.00	Pumps	B.Conc	100
40.00	Pumps	B.Conc	15
50.00	Controller	Stop	

Chromatogram

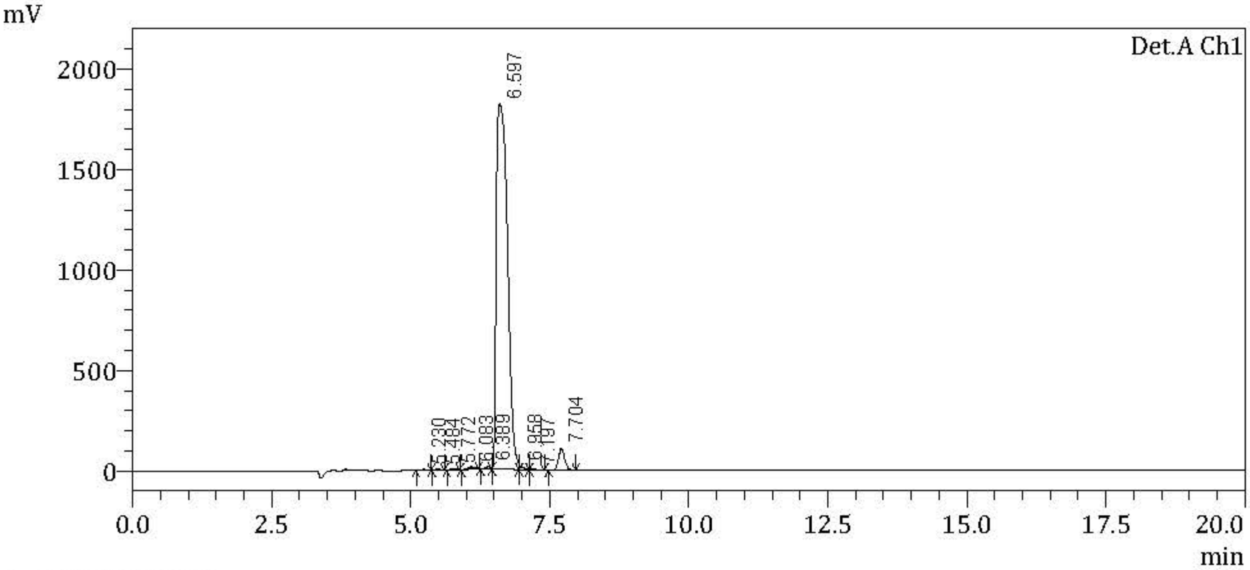

1 Det.A Ch1/220nm

PeakTable

Detector A Ch1 220nm

Peak#	Ret. Time	Area	Height	Area %	Height %
1	5.230	37337	4678	0.141	0.234
2	5.484	29523	4948	0.111	0.248
3	5.772	53211	7985	0.201	0.400
4	6.083	104140	12167	0.392	0.610
5	6.389	84556	13620	0.319	0.683
6	6.597	25301814	1818823	95.338	91.156
7	6.958	89806	20069	0.338	1.006
8	7.197	22072	3260	0.083	0.163
9	7.704	816492	109745	3.077	5.500
Total		26538951	1995296	100.000	100.000

ALK7

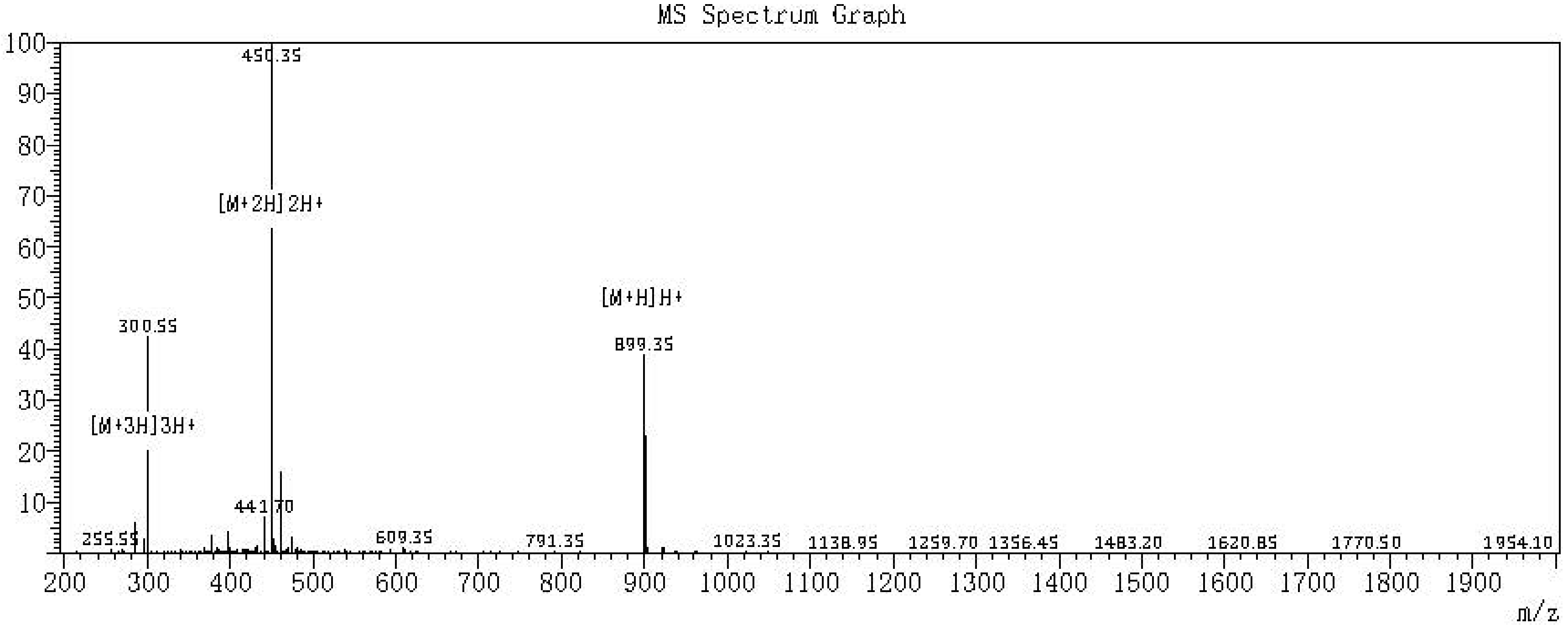

Sample Information

Dissolution method	:5%HAC+8%ACN+87%H2O	Interface	:ESI	Prerod Bias	:+1.5kv
Modified Date	:2021/04/25	Nebulizing Gas Flow	:1.50L/min	Detector	:-0.2kv
Injection Volume	:1ul	CDL Temp	:250C	T.Flow	:0.2ml/min
Heat Block Temp	:200	CDL Volt	:0v	B.conc	:50%H2O/50%MEOH

Order ID	:GP120424-2
Name	:P468_S474
Sequence	:PRFHAWS-NH2
Lot.No	:GP120424-2-0413
Theoretical	:899.00
Observed	:898.70

安徽省国平药业有限公司

CERTIFICATE OF ANALYSIS

**Order ID**	GP120424-1
**Name**	P737_G743
**Lot No.**	GP120424-1-0413
**Sequence**	PAFNAWG-NH2
**Dissolution condition**	100%H2O
**Length**	7AA
**Modification**	N/A
**Molecular Weight (MW)**	760.83
**Storage**	-20°C

**Test Items**	**Specifications**	**Results**
**MW by MS**	760.20	Conforms
**Purity by HPLC**	>95%	96.721%
**Peptide Content**	N/A	N/A
**Moisture content**	N/A	N/A
**Acetic acid content**	N/A	N/A
**Appearance**	Light yellow lyophilized powder	Conforms
**Quantity**	2mg	2.0mg

Certified by: LiuHui　　Date 04/30/2021

Quality Assurance Department

Note: this product is intended for research use only; not for diagnostic or human use.

Guoping Pharmaceutical Co., LTD

地址:合肥市经开区桃花工业园拓展区工投立恒工业广场A2西F1,电话:0551-62841987 传真:0551-62841765 www.guopingyaoye.com

ALK8

Sample Information

Order ID	:GP120424-1
Name	:P737_G743
Sequence	:PAFNAWG-NH2
Lot.No	:GP120424-1-0413
Pump A	:0.1%Trifluoroacetic in 100% water
Pump B	:0.1%Trifluoroacetic in 100% acetonitrile
Total Flow	:1ml/min
Wavelength	:220nm
Analytical column type	:SHIMADZU Inertsil ODS-SP(4.6*250mm*5um)
Dissolution method	:100%H2O
Inj. Volume	:22 uL

Time	Module	Action	Value
0.01	Pumps	B.Conc	10
20.00	Pumps	B.Conc	50
30.00	Pumps	B.Conc	100
38.00	Pumps	B.Conc	100
40.00	Pumps	B.Conc	10
50.00	Controller	Stop	

Chromatogram

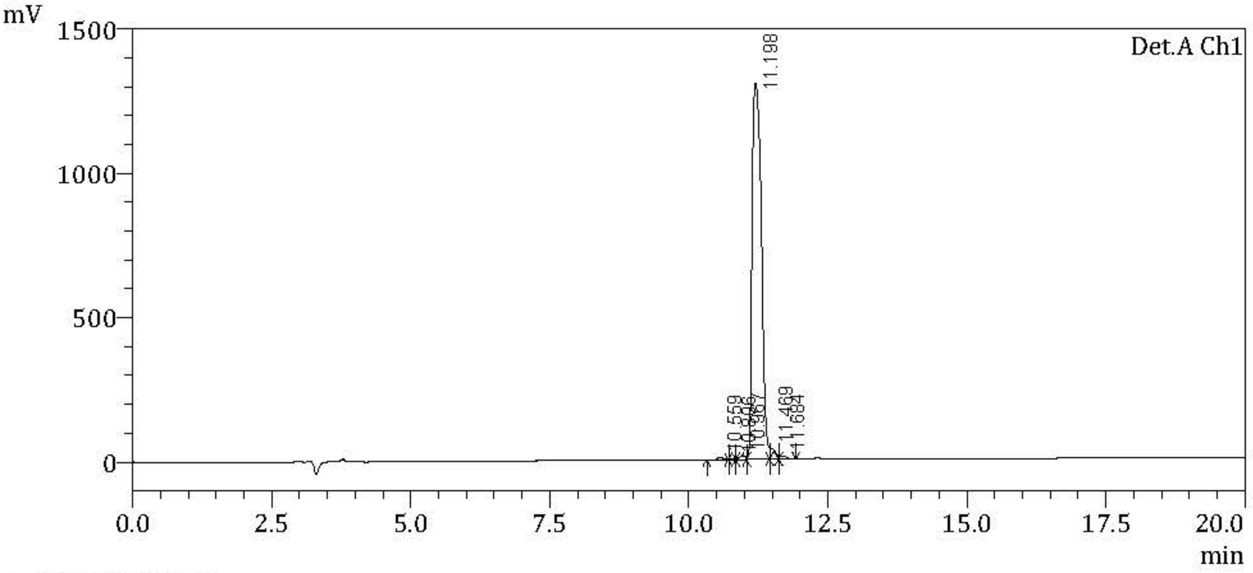

1 Det.A Ch1/220nm

PeakTable

Detector A Ch1 220nm

Peak#	Ret. Time	Area	Height	Area %	Height %
1	10.559	60316	8654	0.390	0.629
2	10.806	14764	2706	0.095	0.197
3	10.967	103484	14490	0.669	1.053
4	11.198	14958992	1301459	96.721	94.578
5	11.469	247090	38254	1.598	2.780
6	11.684	81519	10510	0.527	0.764
Total		15466165	1376074	100.000	100.000

ALK8

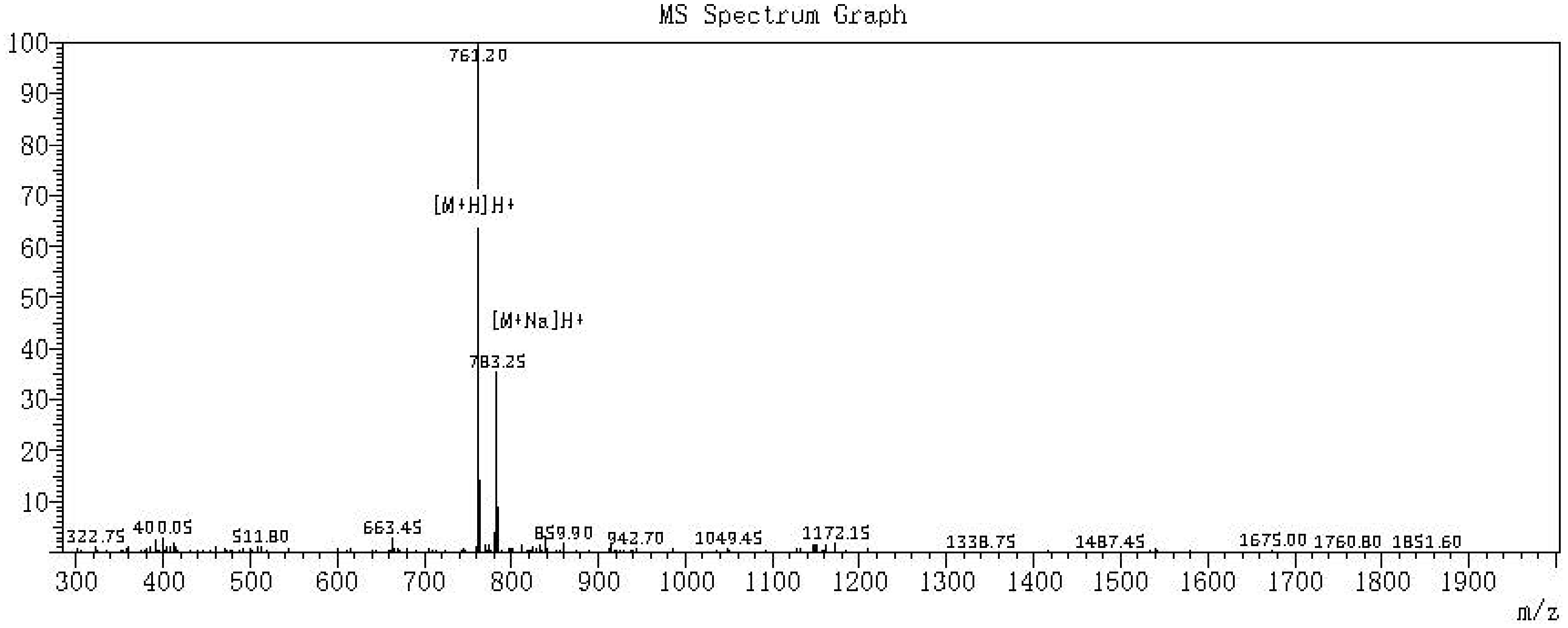

Sample Information

Dissolution method	:5%HAC+8%ACN+87%H2O	Interface	:ESI	Prerod Bias	:+1.5kv
Modified Date	:2021/04/23	Nebulizing Gas Flow	:1.50L/min	Detector	:-0.2kv
Injection Volume	:1ul	CDL Temp	:250C	T.Flow	:0.2ml/min
Heat Block Temp	:200	CDL Volt	:0v	B.conc	:50%H2O/50%MEOH

Order ID	:GP120424-1
Name	:P737_G743
Sequence	:PAFNAWG-NH2
Lot.No	:GP120424-1-0413
Theoretical	:760.83
Observed	:760.20

CERTIFICATE OF ANALYSIS

**Product Name**	P765_S771
**Lot No**	JT-91975
**Sequence**	PAFSPWS-NH2
**Dissolution condition**	15%ACN+85%H2O
**Length**	7AA
**Modification**	N/A
**Molecular Weight (MW)**	789.86
**Storage**	-20℃

**Test Items**	**Specifications**	**Results**
**Purity by HPLC**	95%	98.38%
**Peptide Content**	N/A	N/A
**Moisture content**	N/A	N/A
**Acetic acid content**	N/A	N/A
**Appearance**	White to off-white lyophilized powder	Conforms
**Quantity**	2mg	2.0mg

Certified by:
Quality Assurance Department

Date 10-22-2020

Note: this product is intended for research use only; not for diagnostic or human use.

Synpeptide Co., Ltd

Address: 320 Lvlin Road, Beicai, Pudong new area, Shanghai, China 201204

Tel: +86-21-50835580 Fax: +86-21-50835580 P/C: 201204 http://www.synpeptide.com

ALK9

Sample Information

Order ID : Syn-91975
Name : P765_S771
Sequence : PAFSPWS-NH2
Lot No : JT-91975
Pump A : 0.1% Trifluoroacetic in 100% Water
Pump B : 0.1% Trifluoroacetic in 100% Acetonrtrile
Total Flow : 1ml/min
Wavelength : 220nm
Analytial column type : SHIMADZU Inertsil ODS-SP（4.6*250mm*5um）
Inj. Volume : 30ul

Time	Module	Action	Value
0.00	Pumps	B.Conc	15
25.00	Pumps	B.Conc	75
25.01	Pumps	B.Conc	100
30.00	Pumps	B.Conc	100
30.01	Pumps	Stop	

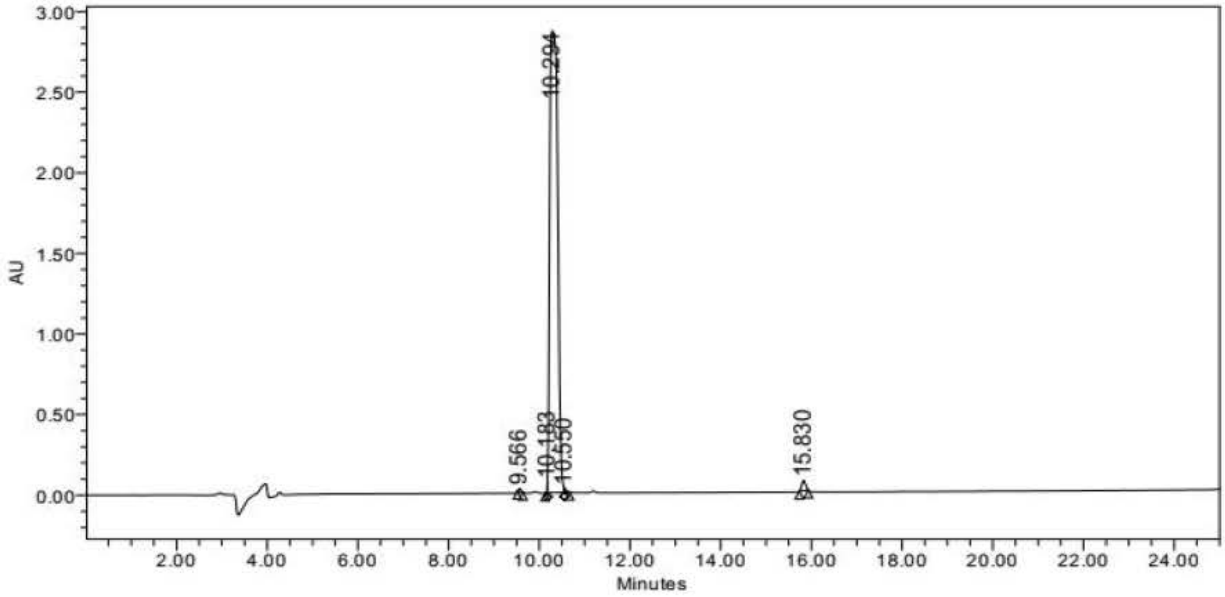

	RT	Area	% Area	Height
1	9.566	77434	0.22	19095
2	10.183	42488	0.12	68143
3	10.294	35015735	98.38	2880716
4	10.550	72108	0.20	24820
5	15.830	384397	1.08	62106

Synpeptide Co., Ltd

Address: 320 Lvlin Road, Beicai, Pudong new area, Shanghai, China 201204
Tel: +86-21-50835580 Fax: +86-21-50835580 P/C: 201204 http://www.synpeptide.com

ALK9

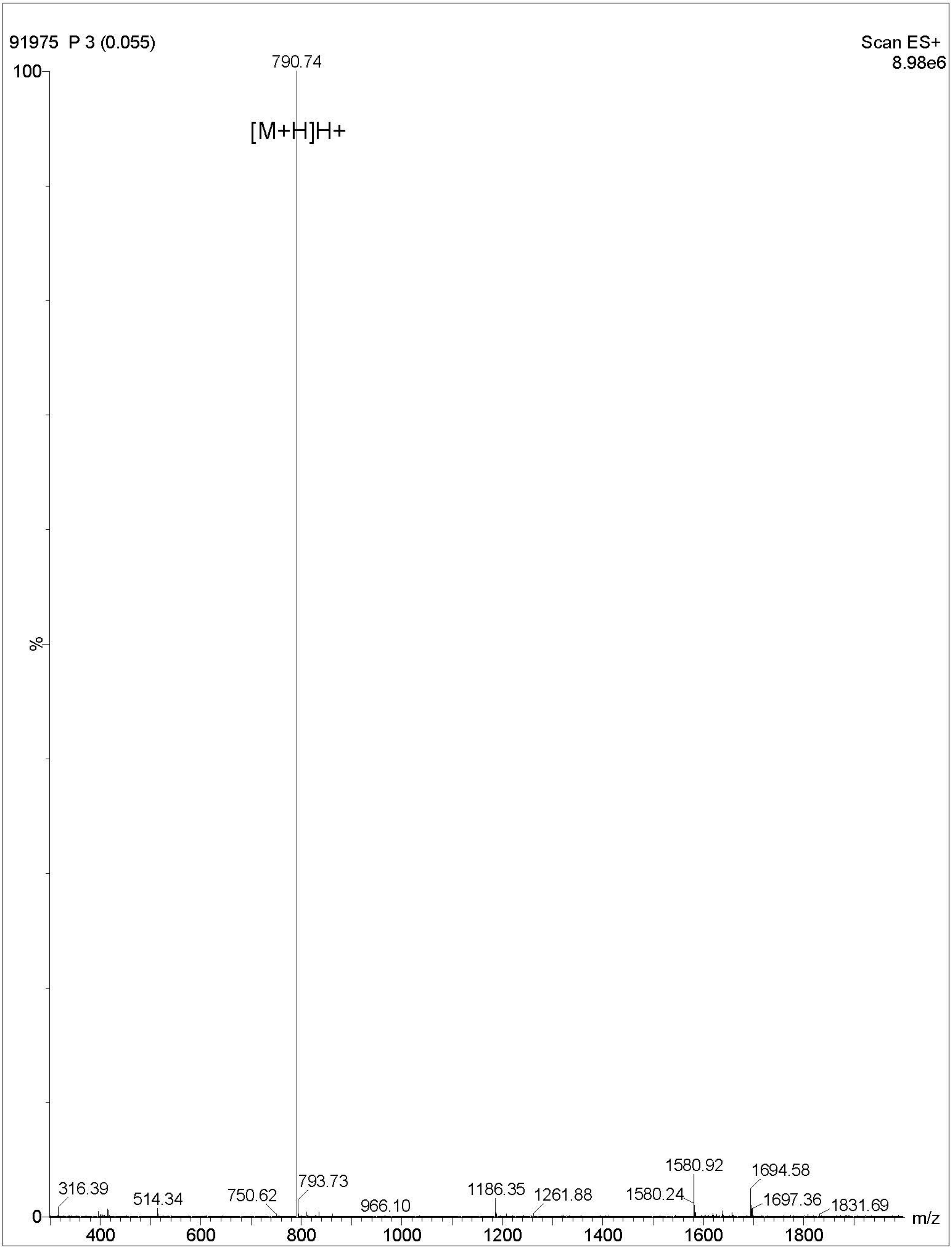

91975 P 3 (0.055)
Scan ES+
8.98e6
100
%
0
790.74
[M+H]H+
316.39
514.34
750.62
793.73
966.10
1186.35
1261.88
1580.24
1580.92
1694.58
1697.36
1831.69
400
600
800
1000
1200
1400
1600
1800
m/z

CERTIFICATE OF ANALYSIS

**Product Name**	P912_G918
**Lot No**	JT-91973
**Sequence**	PAFHAWG-NH2
**Dissolution condition**	15%ACN+85%H2O
**Length**	7AA
**Modification**	N/A
**Molecular Weight (MW)**	783.87
**Storage**	-20℃

**Test Items**	**Specifications**	**Results**
**Purity by HPLC**	95%	99.63%
**Peptide Content**	N/A	N/A
**Moisture content**	N/A	N/A
**Acetic acid content**	N/A	N/A
**Appearance**	White to off-white lyophilized powder	Conforms
**Quantity**	2mg	2.0mg

Certified by:
Quality Assurance Department

Date 10-22-2020

Note: this product is intended for research use only; not for diagnostic or human use.

Synpeptide Co., Ltd

Address: 320 Lvlin Road, Beicai, Pudong new area, Shanghai, China 201204

Tel: +86-21-50835580 Fax: +86-21-50835580 P/C: 201204 http://www.synpeptide.com

ALK10

Sample Information

Order ID : Syn-91973
Name : P912_G918
Sequence : PAFHAWG-NH2
Lot No : JT-91973
Pump A : 0.1% Trifluoroacetic in 100% Water
Pump B : 0.1% Trifluoroacetic in 100% Acetonrtrile
Total Flow : 1ml/min
Wavelength : 220nm
Analytial column type : SHIMADZU Inertsil ODS-SP（4.6*250mm*5um）
Inj. Volume : 30ul

Time	Module	Action	Value
0.00	Pumps	B.Conc	10
25.00	Pumps	B.Conc	70
25.01	Pumps	B.Conc	100
30.00	Pumps	B.Conc	100
30.01	Pumps	Stop	

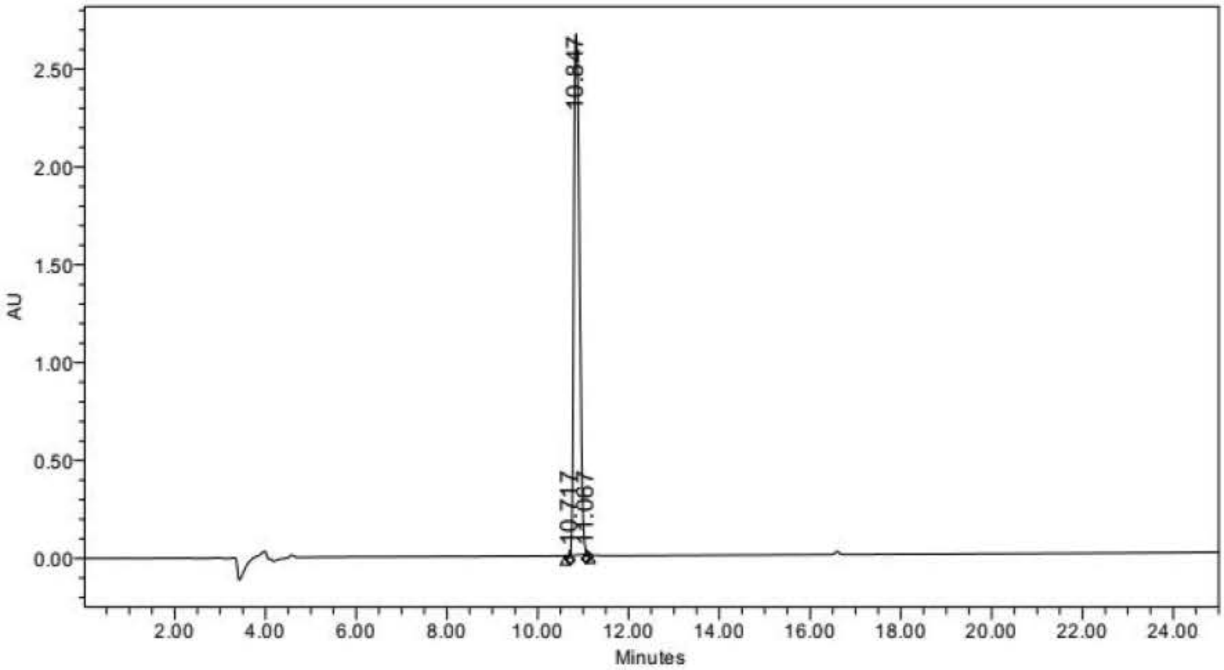

	RT	Area	% Area	Height
1	10.717	27600	0.12	24152
2	10.847	23248034	99.63	2673313
3	11.067	58960	0.25	19041

Synpeptide Co., Ltd

Address: 320 Lvlin Road, Beicai, Pudong new area, Shanghai, China 201204
Tel: +86-21-50835580 Fax: +86-21-50835580 P/C: 201204 http://www.synpeptide.com

ALK10

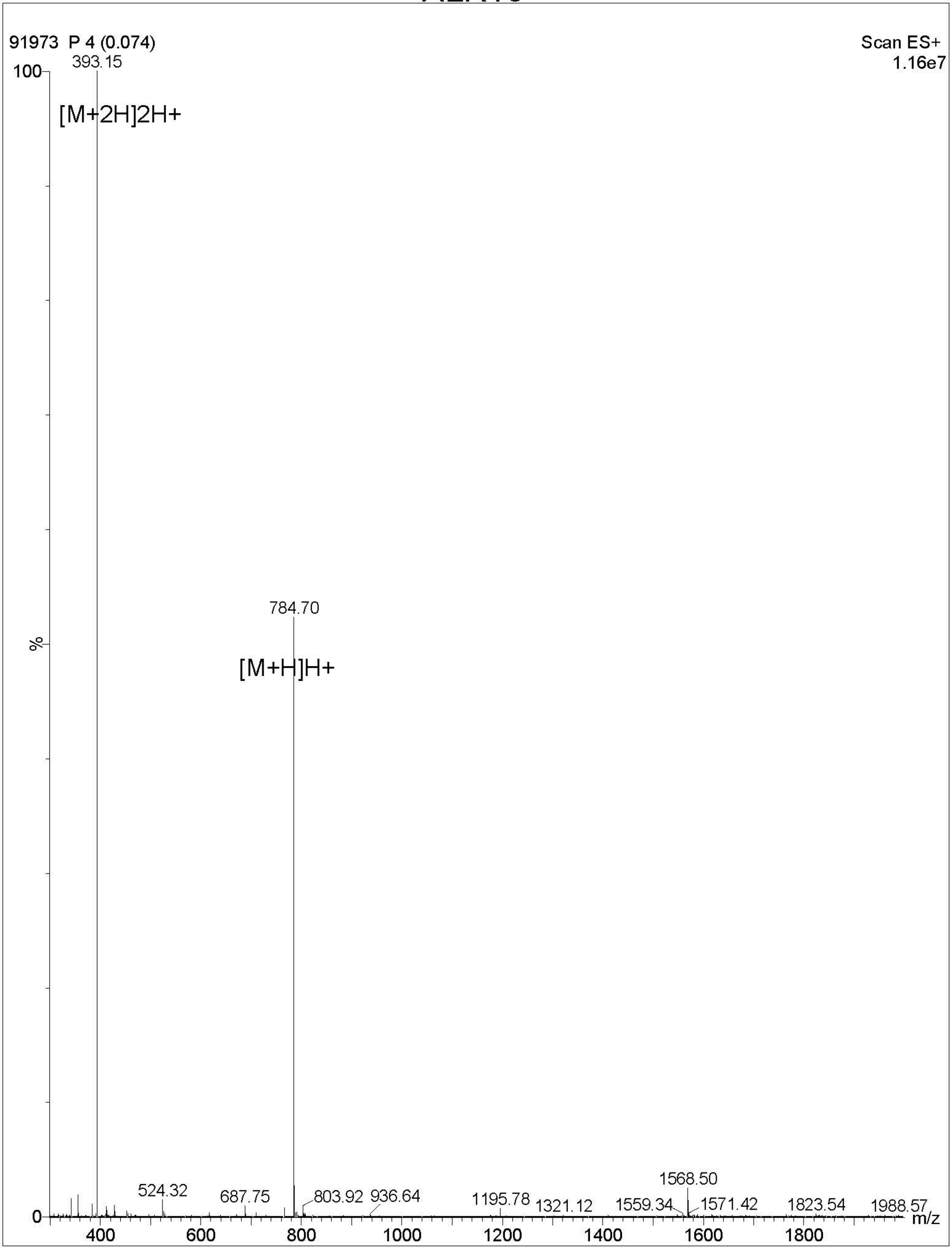
91973 P 4 (0.074)
Scan ES+
1.16e7
100
393.15
[M+2H]2H+
784.70
[M+H]H+
%
524.32
687.75
803.92
936.64
1195.78
1321.12
1559.34
1568.50
1571.42
1823.54
1988.57
0
m/z
400
600
800
1000
1200
1400
1600
1800

CERTIFICATE OF ANALYSIS

**Product Name**	P1273_S1280
**Lot No**	JT-91977
**Sequence**	PAAFHAWS-NH2
**Dissolution condition**	15%ACN+85%H2O
**Length**	8AA
**Modification**	N/A
**Molecular Weight (MW)**	884.96
**Storage**	-20℃

**Test Items**	**Specifications**	**Results**
**Purity by HPLC**	95%	98.77%
**Peptide Content**	N/A	N/A
**Moisture content**	N/A	N/A
**Acetic acid content**	N/A	N/A
**Appearance**	White to off-white lyophilized powder	Conforms
**Quantity**	2mg	2.0mg

Certified by:
Quality Assurance Department

Date 10-29-2020

Note: this product is intended for research use only; not for diagnostic or human use.

Synpeptide Co., Ltd

Address: 320 Lvlin Road, Beicai, Pudong new area, Shanghai, China 201204

Tel: +86-21-50835580 Fax: +86-21-50835580 P/C: 201204 http://www.synpeptide.com

ALK11

Sample Information

Order ID	: Syn-91977
Name	: P1273_S1280
Sequence	: PAAFHAWS-NH2
Lot No	: JT-91977
Pump A	: 0.1% Trifluoroacetic in 100% Water
Pump B	: 0.1% Trifluoroacetic in 100% Acetonrtrile
Total Flow	: 1ml/min
Wavelength	: 220nm
Analytial column type	: SHIMADZU Inertsil ODS-SP（4.6*250mm*5um）
Inj. Volume	: 30ul

Time	Module	Action	Value
0.00	Pumps	B.Conc	10
25.00	Pumps	B.Conc	70
25.01	Pumps	B.Conc	100
30.00	Pumps	B.Conc	100
30.01	Pumps	Stop	

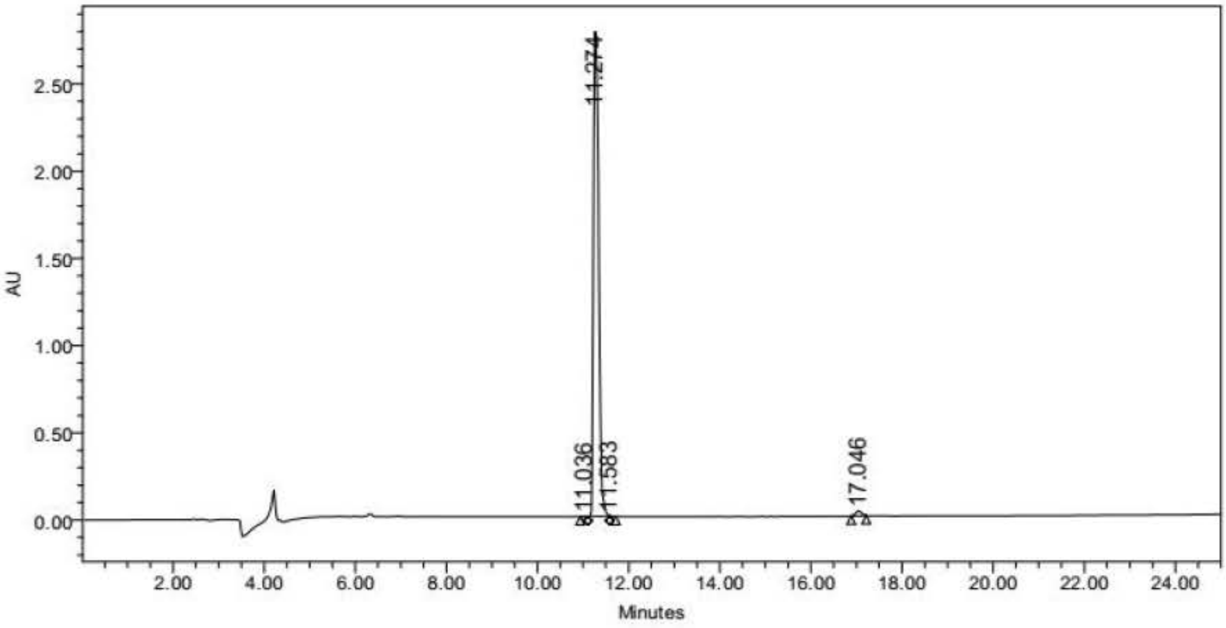

	RT	Area	% Area	Height
1	11.036	16351	0.07	2890
2	11.274	22440991	98.77	2821205
3	11.583	11456	0.05	4328
4	17.046	252751	1.11	27369

Synpeptide Co., Ltd

Address: 320 Lvlin Road, Beicai, Pudong new area, Shanghai, China 201204

Tel: +86-21-50835580 Fax: +86-21-50835580 P/C: 201204 http://www.synpeptide.com

ALK11

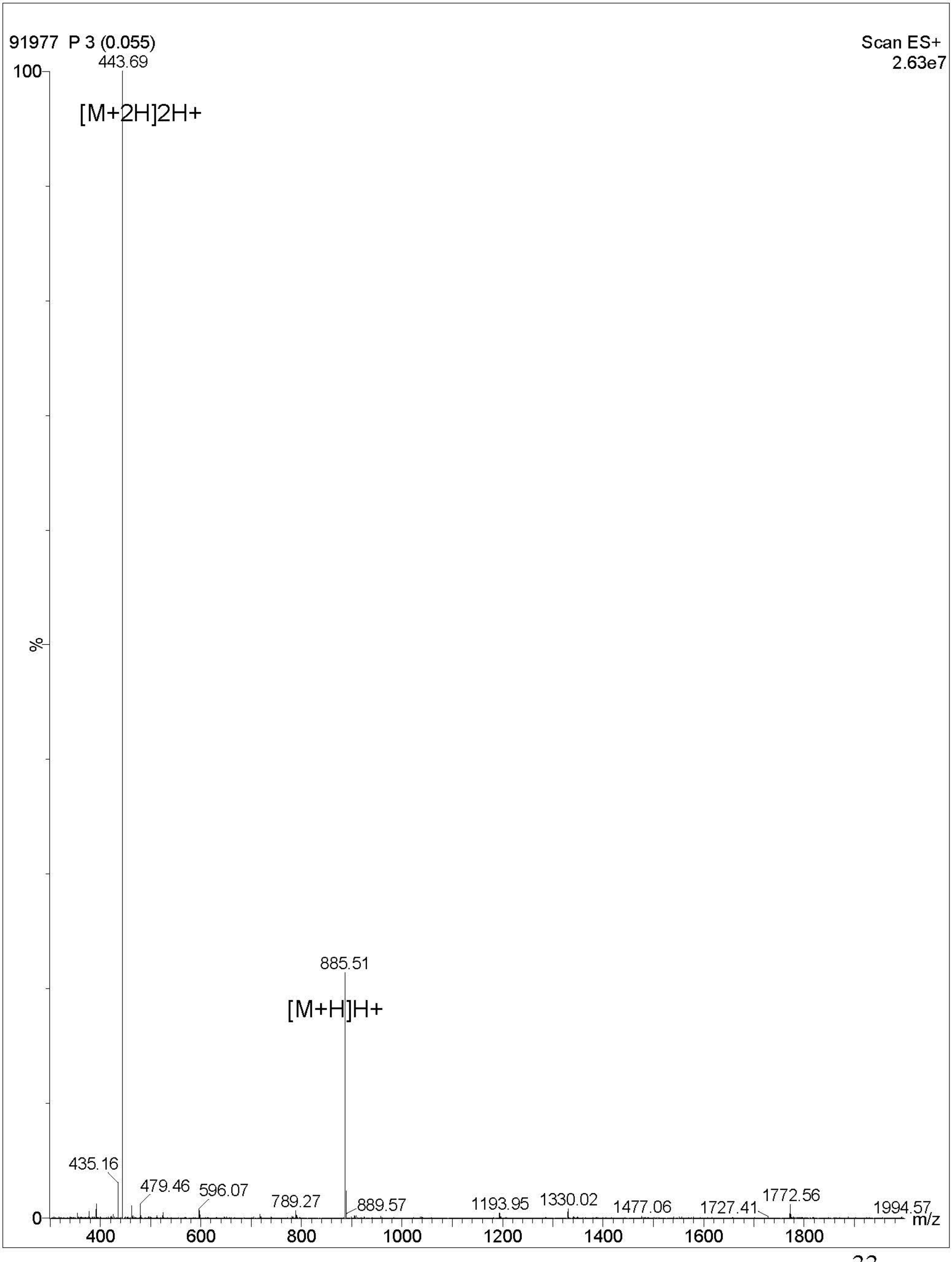

91977 P 3 (0.055)
Scan ES+
2.63e7
100
443.69
[M+2H]2H+
%
885.51
[M+H]H+
435.16
479.46
596.07
789.27
889.57
1193.95
1330.02
1477.06
1727.41
1772.56
1994.57
0
400
600
800
1000
1200
1400
1600
1800
m/z

安徽省国平药业有限公司

CERTIFICATE OF ANALYSIS

**Order ID**	GP120424-4
**Name**	L1405_S1412
**Lot No.**	GP120424-4-0413
**Sequence**	LAAFHAWS-NH2
**Dissolution condition**	100%H2O
**Length**	8AA
**Modification**	N/A
**Molecular Weight (MW)**	901.01
**Storage**	-20°C

**Test Items**	**Specifications**	**Results**
**MW by MS**	900.80	Conforms
**Purity by HPLC**	>95%	96.566%
**Peptide Content**	N/A	N/A
**Moisture content**	N/A	N/A
**Acetic acid content**	N/A	N/A
**Appearance**	White to off-white lyophilized powder	Conforms
**Quantity**	2mg	2.0mg

Certified by: LiuHui Date 04/30/2021

Quality Assurance Department

Note: this product is intended for research use only; not for diagnostic or human use.

Guoping Pharmaceutical Co., LTD

地址:合肥市经开区桃花工业园拓展区工投立恒工业广场A2西F1,电话:0551-62841987 传真:0551-62841765 www.guopingyaoye.com

Sample Information

Order ID :GP120424-4
Name :L1405_S1412
Sequence :LAAFHAWS-NH2
Lot.No :GP120424-4-0413
Pump A :0.1%Trifluoroacetic in 100% water
Pump B :0.1%Trifluoroacetic in 100% acetonitrile
Total Flow :1ml/min
Wavelength :220nm
Analytical column type :SHIMADZU Inertsil ODS-SP(4.6*250mm*5um)
Dissolution method :100%H2O
Inj. Volume :30 uL

Time	Module	Action	Value
0.01	Pumps	B.Conc	10
20.00	Pumps	B.Conc	50
30.00	Pumps	B.Conc	100
38.00	Pumps	B.Conc	100
40.00	Pumps	B.Conc	10
50.00	Controller	Stop	

Chromatogram

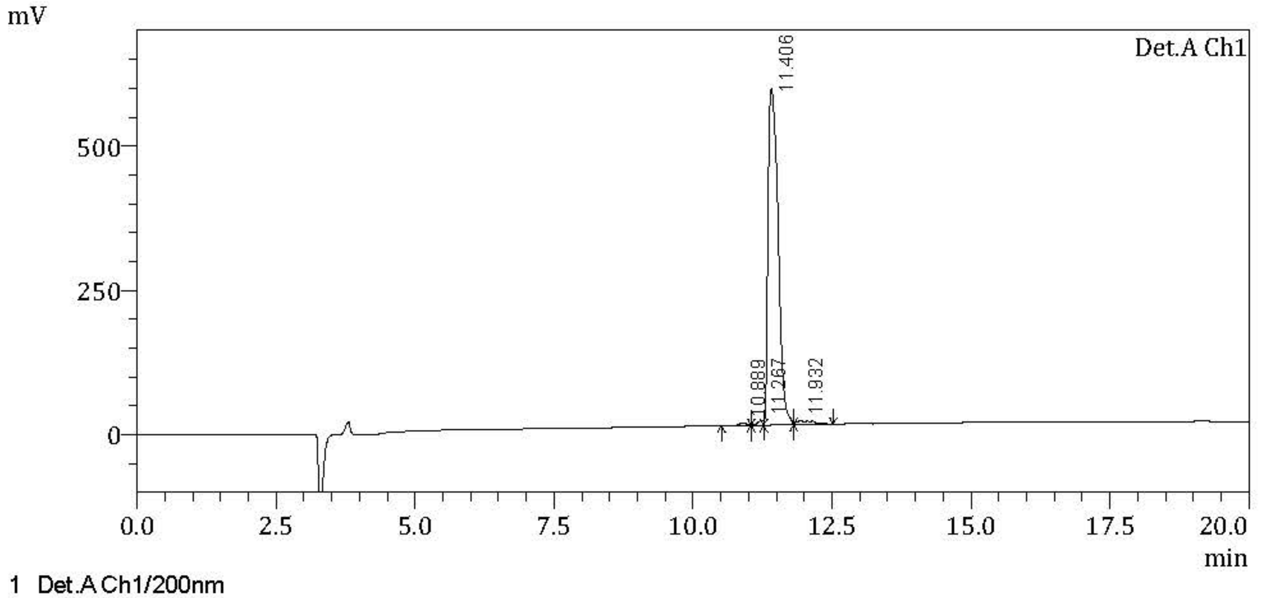

1 Det.A Ch1/200nm

PeakTable

Detector A Ch1 200nm

Peak#	Ret. Time	Area	Height	Area %	Height %
1	10.889	46502	5157	0.616	0.856
2	11.267	60668	7706	0.803	1.280
3	11.406	7291628	582315	96.566	96.682
4	11.932	152109	7121	2.014	1.182
Total		7550907	602299	100.000	100.000

ALK12

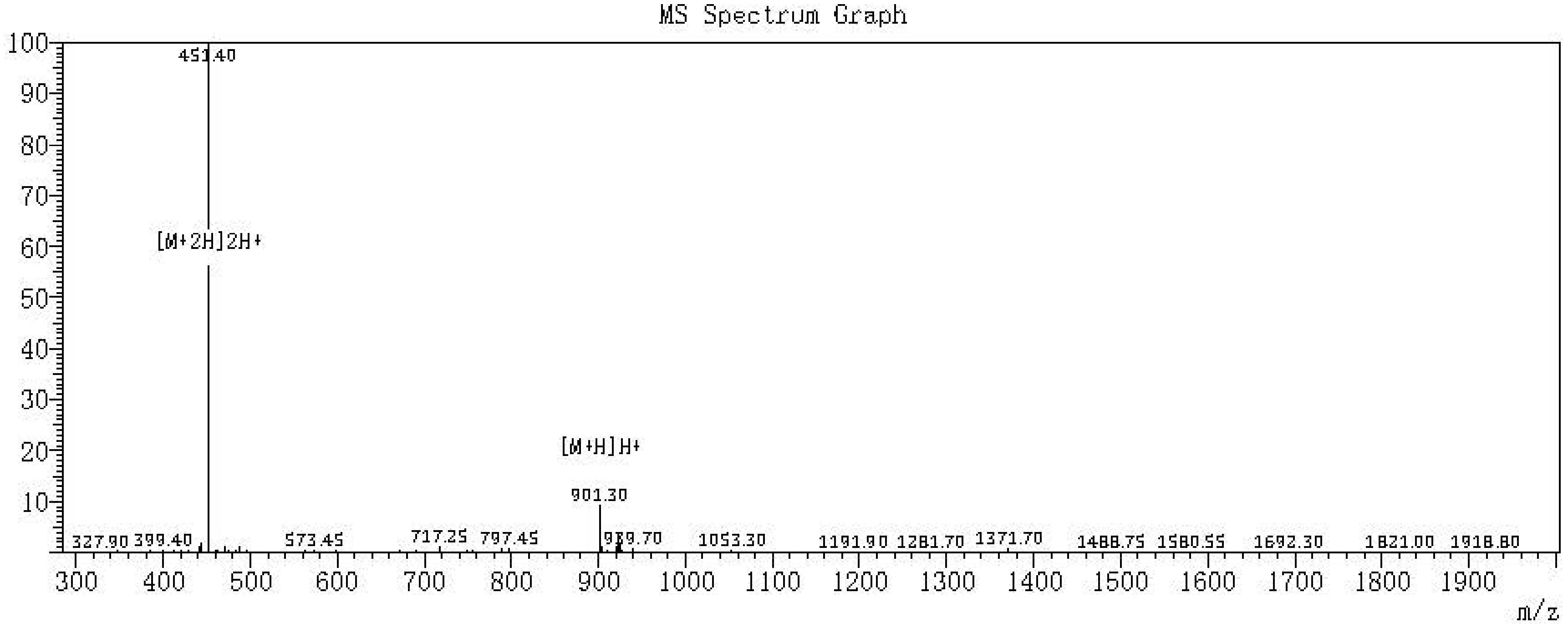

Sample Information

Dissolution method	:5%HAC+8%ACN+87%H2O	Interface	:ESI	Prerod Bias	:+1.5kv
Modified Date	:2021/04/25	Nebulizing Gas Flow	:1.50L/min	Detector	:-0.2kv
Injection Volume	:1ul	CDL Temp	:250C	T.Flow	:0.2ml/min
Heat Block Temp	:200	CDL Volt	:0v	B.conc	:50%H2O/50%MEOH

Order ID	:GP120424-4
Name	:L1405_S1412
Sequence	:LAAFHAWS-NH2
Lot.No	:GP120424-4-0413
Theoretical	:901.01
Observed	:900.80

ALK13

安徽省国平药业有限公司

CERTIFICATE OF ANALYSIS

**Order ID**	GP120424-3
**Name**	P1488_S1494
**Lot No.**	GP120424-3-0413
**Sequence**	PAFQAWS-NH2
**Dissolution condition**	100%H2O
**Length**	7AA
**Modification**	N/A
**Molecular Weight (MW)**	804.88
**Storage**	-20°C

**Test Items**	**Specifications**	**Results**
**MW by MS**	804.25	Conforms
**Purity by HPLC**	>95%	95.190%
**Peptide Content**	N/A	N/A
**Moisture content**	N/A	N/A
**Acetic acid content**	N/A	N/A
**Appearance**	White to off-white lyophilized powder	Conforms
**Quantity**	2mg	2.0mg

Certified by: LiuHui Date 04/30/2021

Quality Assurance Department

Note: this product is intended for research use only; not for diagnostic or human use.

Guoping Pharmaceutical Co., LTD

地址:合肥市经开区桃花工业园拓展区工投立恒工业广场A2西F1,电话:0551-62841987 传真:0551-62841765 www.guopingyaoye.com

ALK13

Sample Information

Order ID :GP120424-3
Name :P1488_S1494
Sequence :PAFQAWS-NH2
Lot.No :GP120424-3-0413
Pump A :0.1%Trifluoroacetic in 100% water
Pump B :0.1%Trifluoroacetic in 100% acetonitrile
Total Flow :1ml/min
Wavelength :220nm
Analytical column type :SHIMADZU Inertsil ODS-SP(4.6*250mm*5um)
Dissolution method :100%H2O
Inj. Volume :18 uL

Time	Module	Action	Value
0.01	Pumps	B.Conc	10
20.00	Pumps	B.Conc	50
30.00	Pumps	B.Conc	100
38.00	Pumps	B.Conc	100
40.00	Pumps	B.Conc	10
50.00	Controller	Stop	

Chromatogram

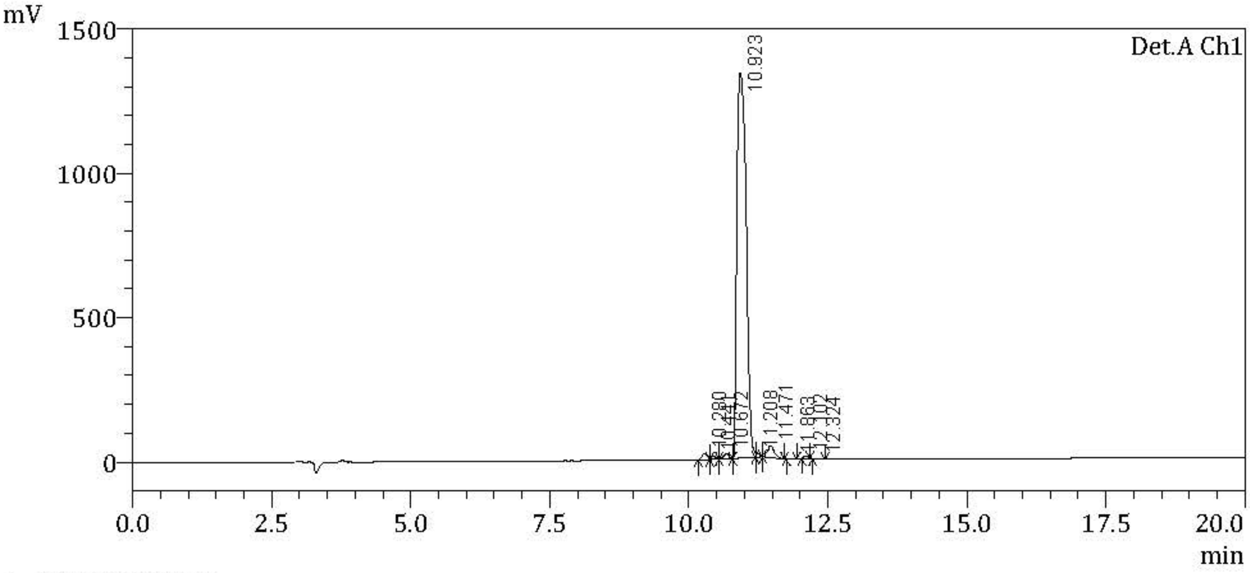

1 Det.A Ch1/220nm

PeakTable

Detector A Ch1 220nm

Peak#	Ret. Time	Area	Height	Area %	Height %
1	10.280	144302	21177	0.897	1.455
2	10.441	59719	10016	0.371	0.688
3	10.672	123868	19300	0.770	1.326
4	10.923	15313543	1335028	95.190	91.717
5	11.208	52139	14613	0.324	1.004
6	11.471	317172	41505	1.972	2.851
7	11.863	13324	2226	0.083	0.153
8	12.102	46074	8845	0.286	0.608
9	12.324	17134	2889	0.107	0.198
Total		16087275	1455598	100.000	100.000

ALK13

MS Spectrum Graph

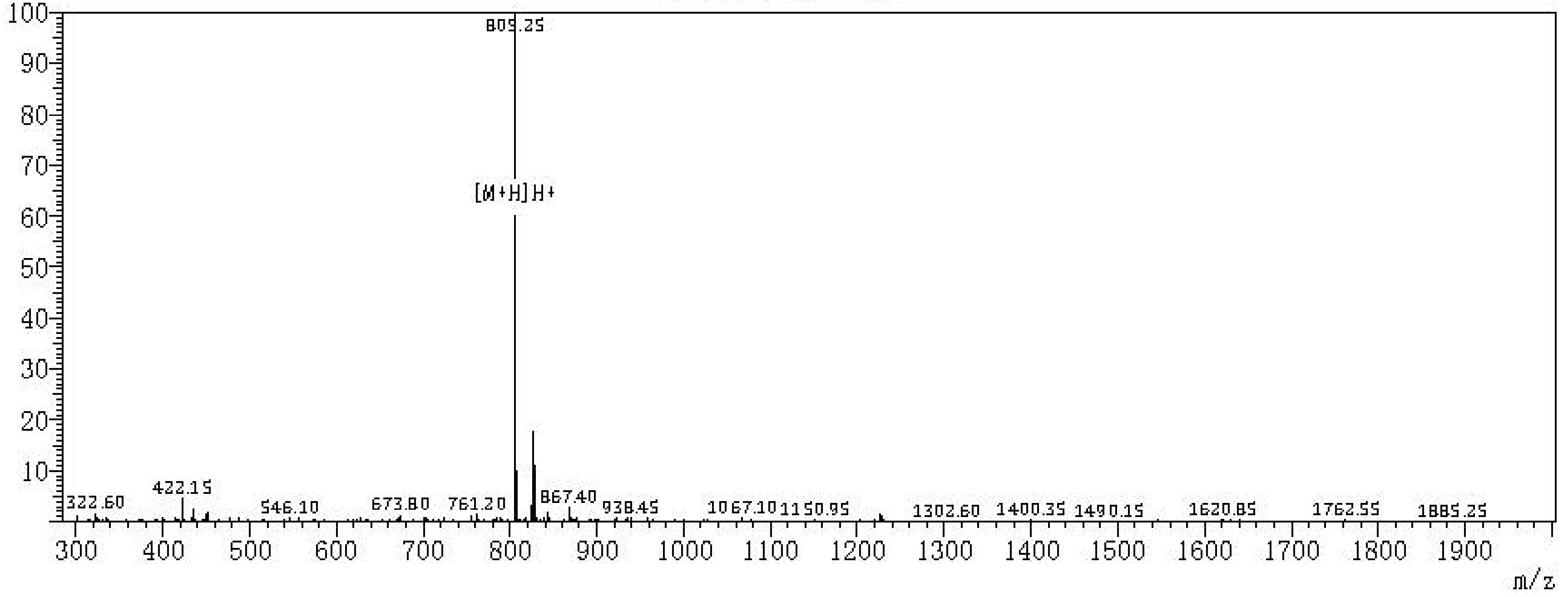

Sample Information

Dissolution method	:5%HAC+8%ACN+87%H2O	Interface	:ESI	Prerod Bias	:+1.5kv
Modified Date	:2021/04/23	Nebulizing Gas Flow	:1.50L/min	Detector	:-0.2kv
Injection Volume	:1ul	CDL Temp	:250C	T.Flow	:0.2ml/min
Heat Block Temp	:200	CDL Volt	:0v	B.conc	:50%H2O/50%MEOH

Order ID	:GP120424-3
Name	:P1488_S1494
Sequence	:PAFQAWS-NH2
Lot.No	:GP120424-3-0413
Theoretical	:804.88
Observed	:804.25

ALK1_A1

CERTIFICATE OF ANALYSIS

**Product Name**	ALK1-A1
**Lot No**	JT-91982
**Sequence**	AAFHSWS-NH2
**Dissolution condition**	15%ACN+85%H2O
**Length**	7AA
**Modification**	N/A
**Molecular Weight (MW)**	803.85
**Storage**	-20℃

**Test Items**	**Specifications**	**Results**
**Purity by HPLC**	95%	95.27%
**Peptide Content**	N/A	N/A
**Moisture content**	N/A	N/A
**Acetic acid content**	N/A	N/A
**Appearance**	White to off-white lyophilized powder	Conforms
**Quantity**	2mg	2.0mg

Certified by:
Quality Assurance Department

Date 10-26-2020

Note: this product is intended for research use only; not for diagnostic or human use.

Synpeptide Co., Ltd

Address: 320 Lvlin Road, Beicai, Pudong new area, Shanghai, China 201204

Tel: +86-21-50835580 Fax: +86-21-50835580 P/C: 201204 http://www.synpeptide.com

ALK_A1

Sample Information

Order ID	: Syn-91982
Name	: ALK1-A1
Sequence	: AAFHSWS-NH2
Lot No	: JT-91982
Pump A	: 0.1% Trifluoroacetic in 100% Water
Pump B	: 0.1% Trifluoroacetic in 100% Acetonrtrile
Total Flow	: 1ml/min
Wavelength	: 220nm
Analytial column type	: SHIMADZU Inertsil ODS-SP（4.6*250mm*5um）
Inj. Volume	: 30ul

Time	Module	Action	Value
0.00	Pumps	B.Conc	5
25.00	Pumps	B.Conc	65
25.01	Pumps	B.Conc	100
30.00	Pumps	B.Conc	100
30.01	Pumps	Stop	

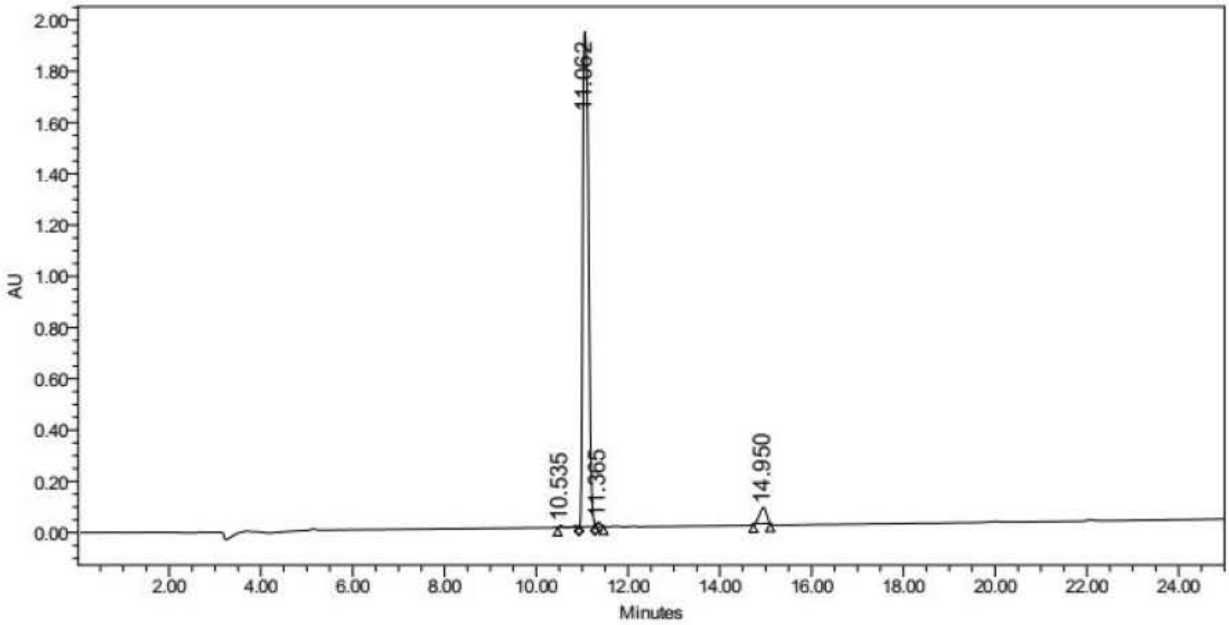

	RT	Area	% Area	Height
1	10.535	58996	0.33	6569
2	11.062	16873650	95.27	1982156
3	11.365	103157	0.58	15684
4	14.950	675724	3.82	62570

Synpeptide Co., Ltd

Address: 320 Lvlin Road, Beicai, Pudong new area, Shanghai, China 201204

Tel: +86-21-50835580 Fax: +86-21-50835580 P/C: 201204 http://www.synpeptide.com

ALK1_A1

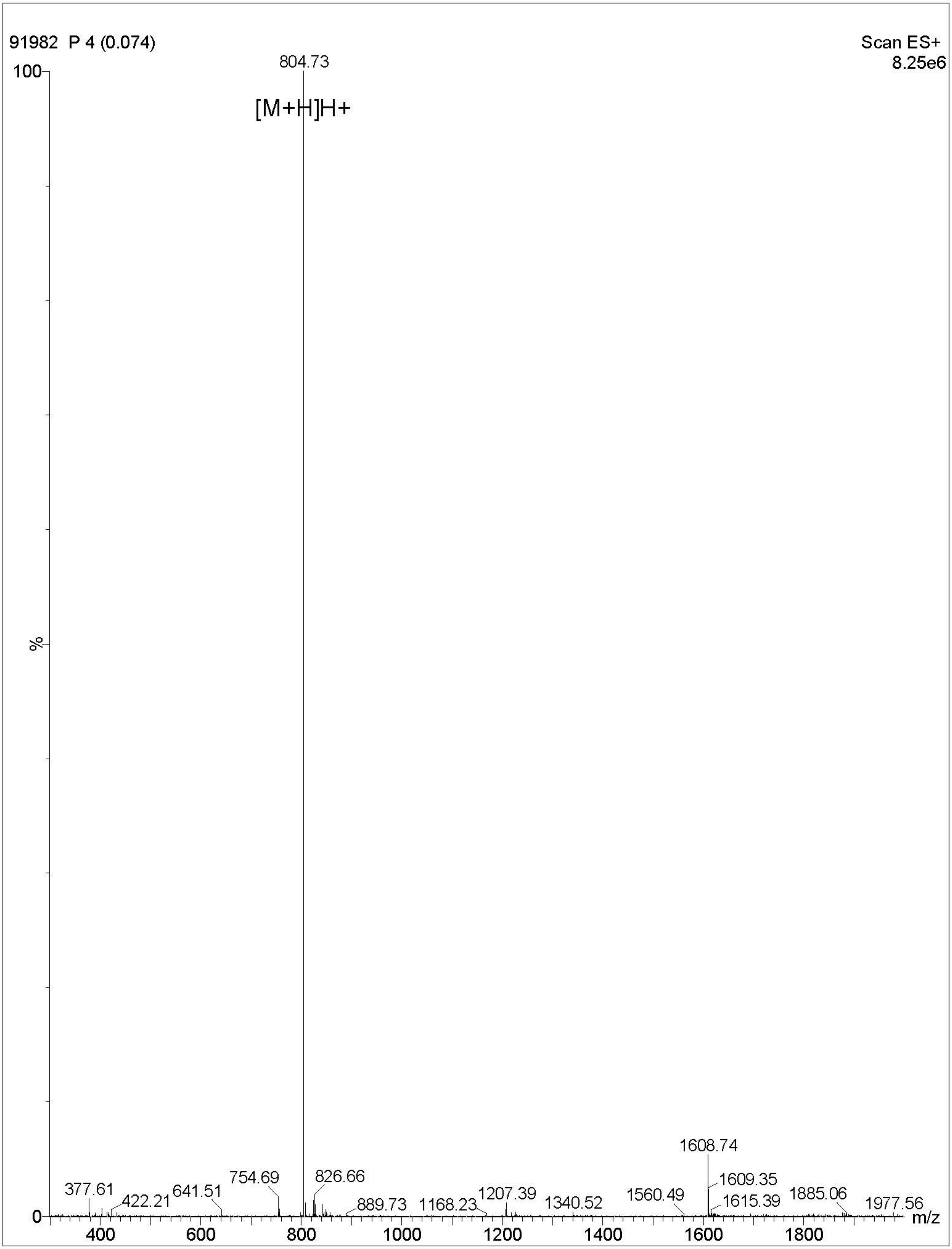
91982 P 4 (0.074)
Scan ES+
8.25e6
804.73
[M+H]H+
100
%
0
377.61
422.21
641.51
754.69
826.66
889.73
1168.23
1207.39
1340.52
1560.49
1608.74
1609.35
1615.39
1885.06
1977.56
400
600
800
1000
1200
1400
1600
1800
m/z

ALK_A3

CERTIFICATE OF ANALYSIS

**Product Name**	ALK1-A3
**Lot No**	JT-91983
**Sequence**	PAAHSWS-NH2
**Dissolution condition**	15%ACN+85%H2O
**Length**	7AA
**Modification**	N/A
**Molecular Weight (MW)**	753.79
**Storage**	-20℃

**Test Items**	**Specifications**	**Results**
**Purity by HPLC**	95%	98.50%
**Peptide Content**	N/A	N/A
**Moisture content**	N/A	N/A
**Acetic acid content**	N/A	N/A
**Appearance**	White to off-white lyophilized powder	Conforms
**Quantity**	2mg	2.0mg

Certified by:
Quality Assurance Department

Date 10-26-2020

Note: this product is intended for research use only; not for diagnostic or human use.

Synpeptide Co., Ltd

Address: 320 Lvlin Road, Beicai, Pudong new area, Shanghai, China 201204

Tel: +86-21-50835580 Fax: +86-21-50835580 P/C: 201204 http://www.synpeptide.com

ALK1_A3

Sample Information

Order ID	: Syn-91983
Name	: ALK1-A3
Sequence	: PAAHSWS-NH2
Lot No	: JT-91983
Pump A	: 0.1% Trifluoroacetic in 100% Water
Pump B	: 0.1% Trifluoroacetic in 100% Acetonrtrile
Total Flow	: 1ml/min
Wavelength	: 220nm
Analytial column type	: SHIMADZU Inertsil ODS-SP（4.6*250mm*5um）
Inj. Volume	: 30ul

Time	Module	Action	Value
0.00	Pumps	B.Conc	5
25.00	Pumps	B.Conc	65
25.01	Pumps	B.Conc	100
30.00	Pumps	B.Conc	100
30.01	Pumps	Stop	

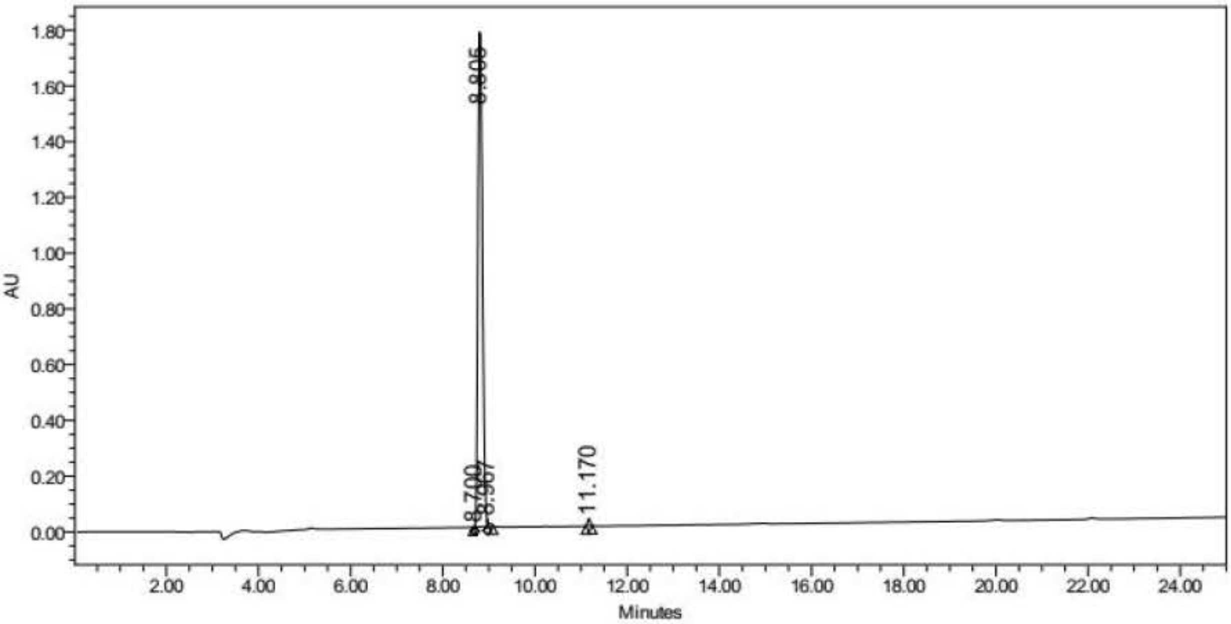

	RT	Area	% Area	Height
1	8.700	4541	0.04	5274
2	8.805	11936895	98.50	1786061
3	8.967	70368	0.58	23049
4	11.170	107037	0.88	24517

Synpeptide Co., Ltd

Address: 320 Lvlin Road, Beicai, Pudong new area, Shanghai, China 201204

Tel: +86-21-50835580 Fax: +86-21-50835580 P/C: 201204 http://www.synpeptide.com

ALK1_A3

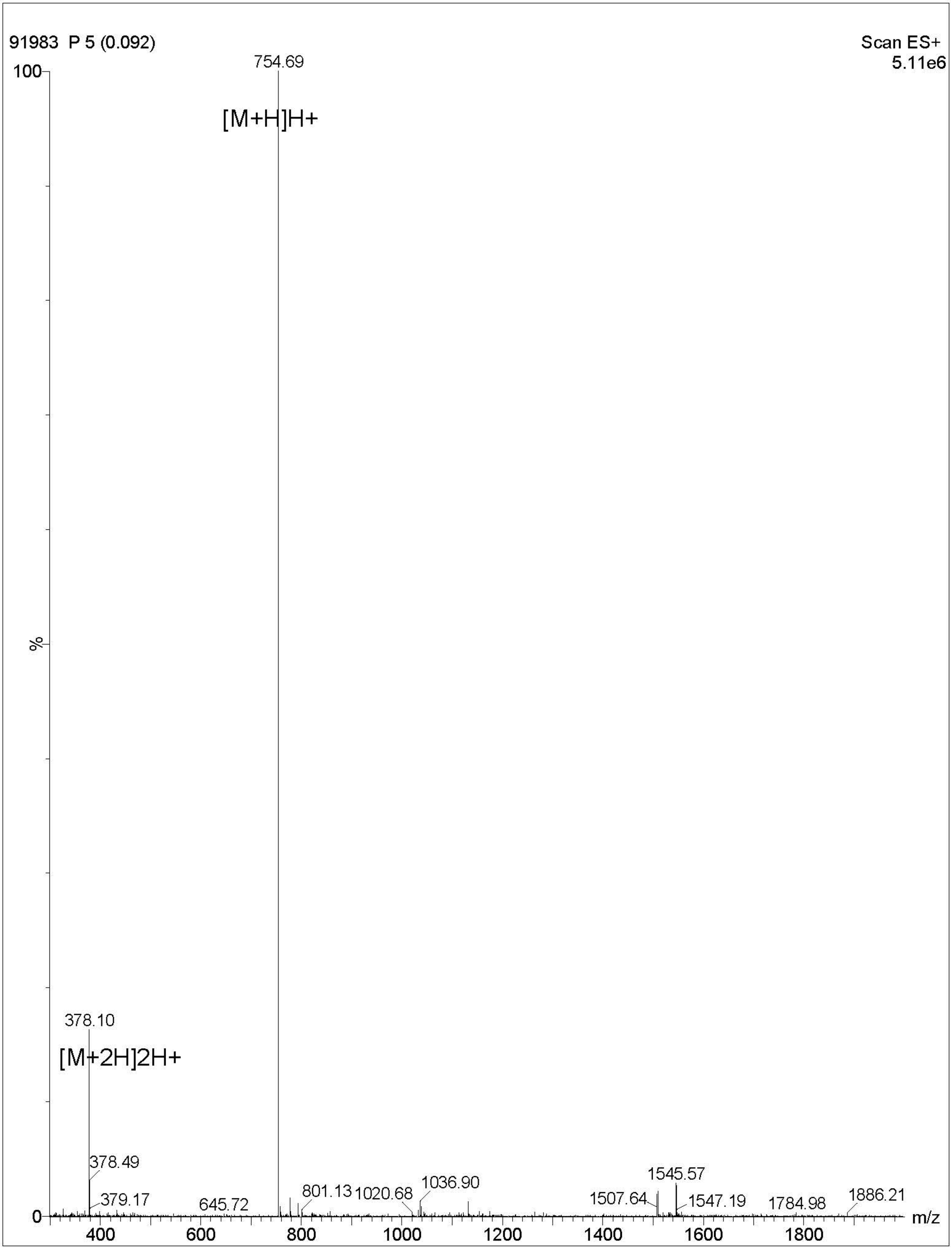

91983 P 5 (0.092)
Scan ES+
5.11e6
100
754.69
[M+H]H+
%
378.10
[M+2H]2H+
378.49
379.17
645.72
801.13
1020.68
1036.90
1507.64
1545.57
1547.19
1784.98
1886.21
0
400
600
800
1000
1200
1400
1600
1800
m/z

ALK1_A4

CERTIFICATE OF ANALYSIS

**Product Name**	ALK1-A4
**Lot No**	JT-91984
**Sequence**	PAFASWS-NH2
**Dissolution condition**	15%ACN+85%H2O
**Length**	7AA
**Modification**	N/A
**Molecular Weight (MW)**	763.82
**Storage**	-20℃

**Test Items**	**Specifications**	**Results**
**Purity by HPLC**	95%	99.66%
**Peptide Content**	N/A	N/A
**Moisture content**	N/A	N/A
**Acetic acid content**	N/A	N/A
**Appearance**	White to off-white lyophilized powder	Conforms
**Quantity**	2mg	2.0mg

Certified by:
Quality Assurance Department

Date 10-27-2020

Note: this product is intended for research use only; not for diagnostic or human use.

Synpeptide Co., Ltd

Address: 320 Lvlin Road, Beicai, Pudong new area, Shanghai, China 201204

Tel: +86-21-50835580 Fax: +86-21-50835580 P/C: 201204 http://www.synpeptide.com

ALK1_A4

Sample Information

Order ID	: Syn-91984		
Name	: ALK1-A4		
Sequence	: PAFASWS-NH2		
Lot No	: JT-91984		
Pump A	: 0.1% Trifluoroacetic in 100% Water		
Pump B	: 0.1% Trifluoroacetic in 100% Acetonrtrile		
Total Flow	: 1ml/min		
Wavelength	: 220nm		
Analytial column type	: SHIMADZU Inertsil ODS-SP（4.6*250mm*5um）		
Inj. Volume	: 30ul		

Time	Module	Action	Value
0.00	Pumps	B.Conc	10
25.00	Pumps	B.Conc	70
25.01	Pumps	B.Conc	100
30.00	Pumps	B.Conc	100
30.01	Pumps	Stop	

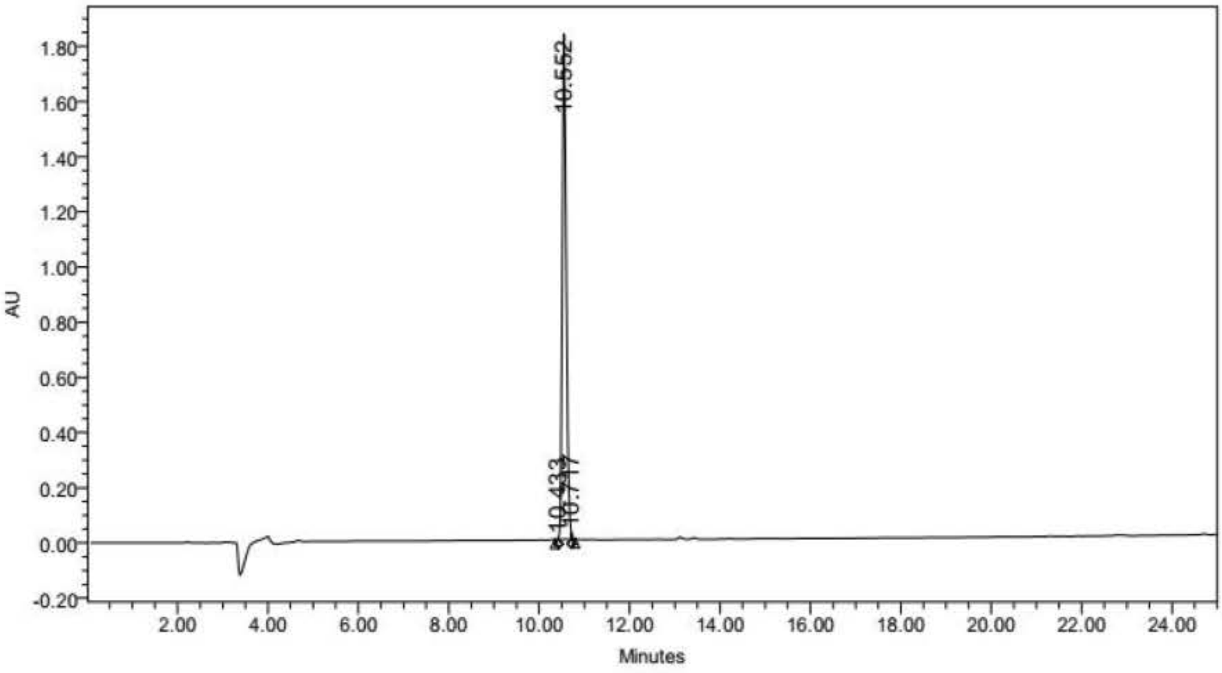

	RT	Area	% Area	Height
1	10.433	5135	0.04	5981
2	10.552	11416754	99.66	1825180
3	10.717	33830	0.30	22506

Synpeptide Co., Ltd

Address: 320 Lvlin Road, Beicai, Pudong new area, Shanghai, China 201204

Tel: +86-21-50835580 Fax: +86-21-50835580 P/C: 201204 http://www.synpeptide.com

ALK1_A4

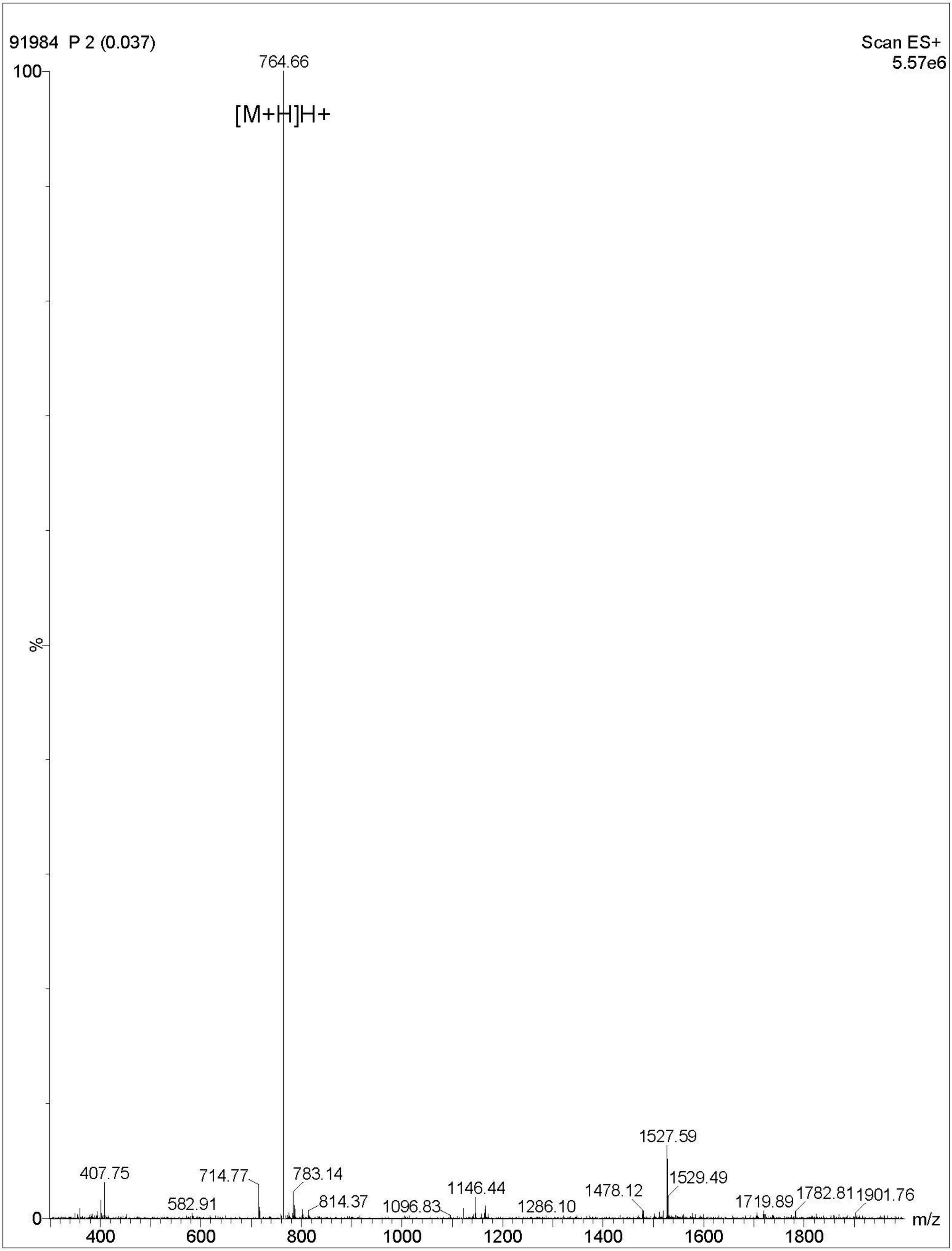

91984 P 2 (0.037)
Scan ES+
5.57e6
100
764.66
[M+H]H+
%
0
407.75
582.91
714.77
783.14
814.37
1096.83
1146.44
1286.10
1478.12
1527.59
1529.49
1719.89
1782.81
1901.76
400
600
800
1000
1200
1400
1600
1800
m/z

ALK1_A6

CERTIFICATE OF ANALYSIS

**Product Name**	ALK1-A6
**Lot No**	JT-91985
**Sequence**	PAFHSAS-NH2
**Dissolution condition**	15%ACN+85%H2O
**Length**	7AA
**Modification**	N/A
**Molecular Weight (MW)**	714.75
**Storage**	-20℃

**Test Items**	**Specifications**	**Results**
**Purity by HPLC**	95%	98.94%
**Peptide Content**	N/A	N/A
**Moisture content**	N/A	N/A
**Acetic acid content**	N/A	N/A
**Appearance**	White to off-white lyophilized powder	Conforms
**Quantity**	2mg	2.0mg

Certified by:
Quality Assurance Department

Date 10-27-2020

Note: this product is intended for research use only; not for diagnostic or human use.

Synpeptide Co., Ltd

Address: 320 Lvlin Road, Beicai, Pudong new area, Shanghai, China 201204

Tel: +86-21-50835580 Fax: +86-21-50835580 P/C: 201204 http://www.synpeptide.com

ALK1_A6

Sample Information

Order ID	: Syn-91985
Name	: ALK1-A6
Sequence	: PAFHSAS-NH2
Lot No	: JT-91985
Pump A	: 0.1% Trifluoroacetic in 100% Water
Pump B	: 0.1% Trifluoroacetic in 100% Acetonrtrile
Total Flow	: 1ml/min
Wavelength	: 220nm
Analytial column type	: SHIMADZU Inertsil ODS-SP（4.6*250mm*5um）
Inj. Volume	: 30ul

Time	Module	Action	Value
0.00	Pumps	B.Conc	1
25.00	Pumps	B.Conc	50
25.01	Pumps	B.Conc	100
30.00	Pumps	B.Conc	100
30.01	Pumps	Stop	

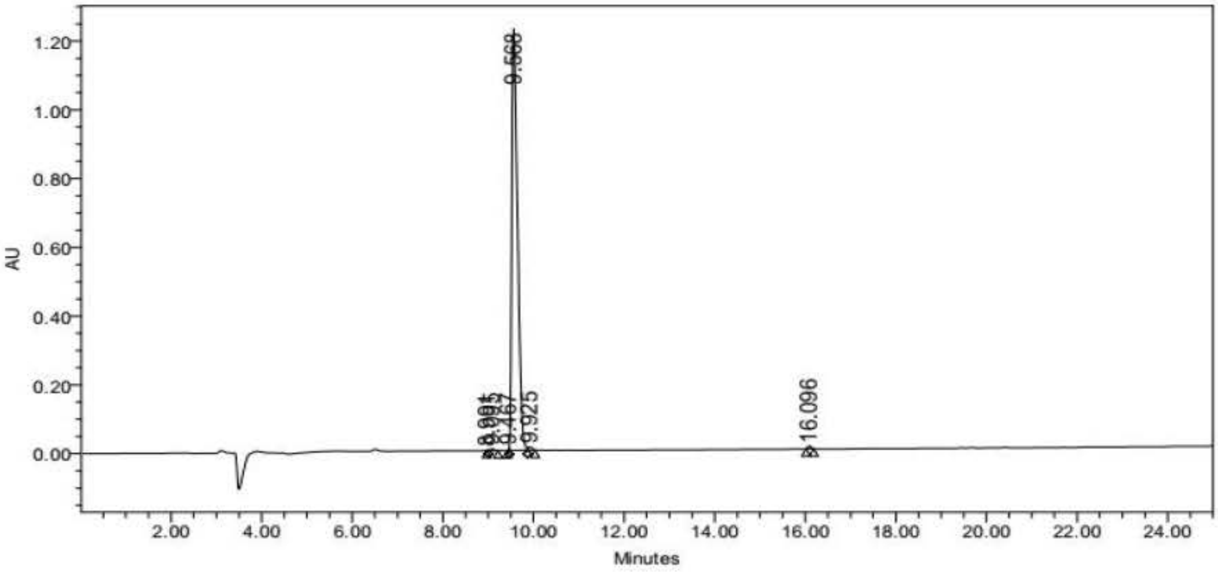

	RT	Area	% Area	Height
1	8.991	788	0.01	386
2	9.095	16752	0.15	3434
3	9.467	4742	0.04	7144
4	9.568	10795895	98.94	1229095
5	9.925	54981	0.50	8367
6	16.096	38516	0.35	7388

Synpeptide Co., Ltd

Address: 320 Lvlin Road, Beicai, Pudong new area, Shanghai, China 201204

Tel: +86-21-50835580 Fax: +86-21-50835580 P/C: 201204 http://www.synpeptide.com

ALK1_A6

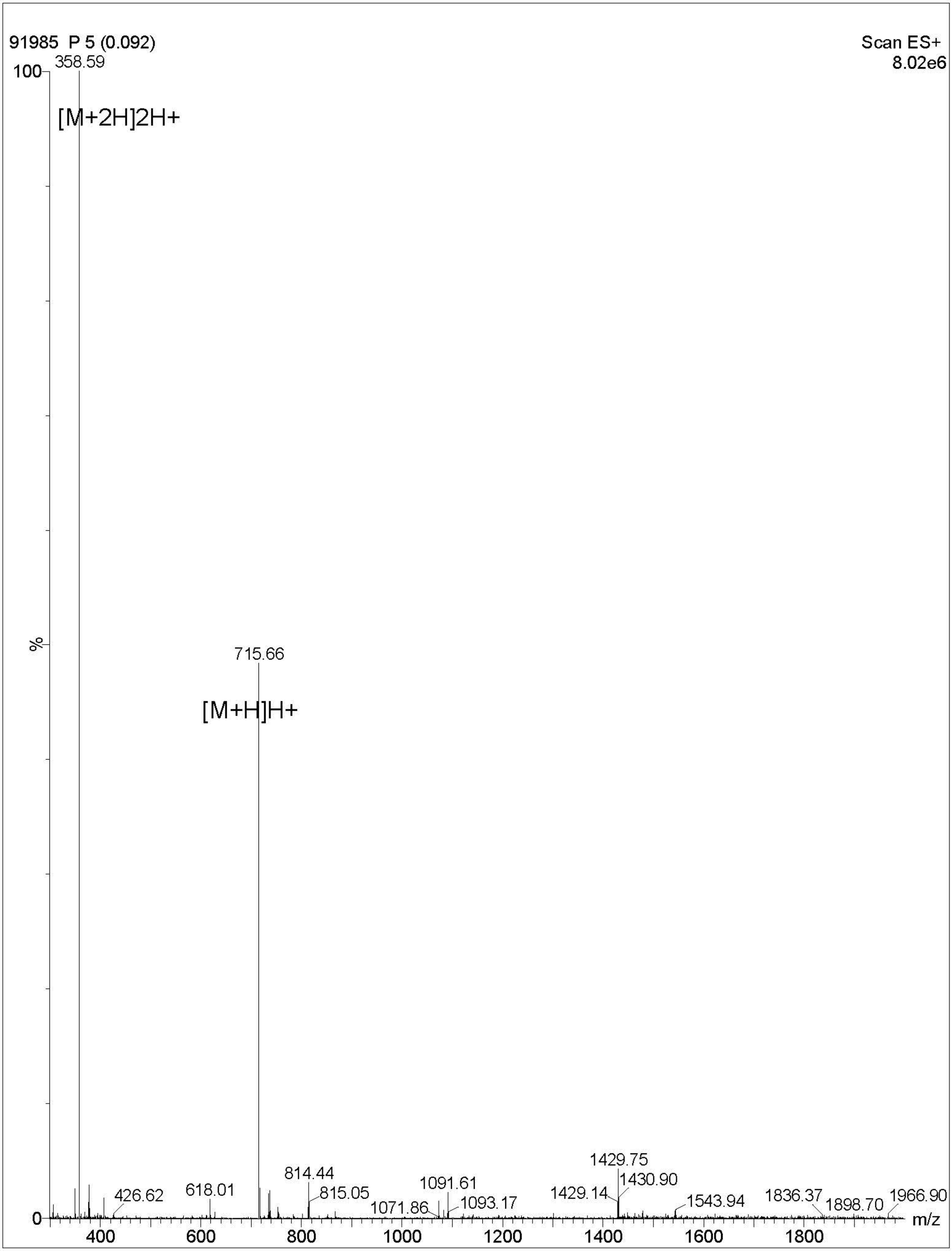
91985 P 5 (0.092)
Scan ES+
8.02e6
100
358.59
[M+2H]2H+
%
715.66
[M+H]H+
814.44
815.05
426.62
618.01
1091.61
1071.86
1093.17
1429.75
1430.90
1429.14
1543.94
1836.37
1898.70
1966.90
0
400
600
800
1000
1200
1400
1600
1800
m/z

ALK1_A7

CERTIFICATE OF ANALYSIS

**Product Name**	ALK1-A7
**Lot No**	JT-91986
**Sequence**	PAFHSWA-NH2
**Dissolution condition**	15%ACN+85%H2O
**Length**	7AA
**Modification**	N/A
**Molecular Weight (MW)**	813.89
**Storage**	-20℃

**Test Items**	**Specifications**	**Results**
**Purity by HPLC**	95%	99.41%
**Peptide Content**	N/A	N/A
**Moisture content**	N/A	N/A
**Acetic acid content**	N/A	N/A
**Appearance**	White to off-white lyophilized powder	Conforms
**Quantity**	2mg	2.0mg

Certified by:
Quality Assurance Department

Date 10-27-2020

Note: this product is intended for research use only; not for diagnostic or human use.

Synpeptide Co., Ltd

Address: 320 Lvlin Road, Beicai, Pudong new area, Shanghai, China 201204

Tel: +86-21-50835580 Fax: +86-21-50835580 P/C: 201204 http://www.synpeptide.com

ALK1_A7

Sample Information

Order ID	: Syn-91986
Name	: ALK1-A7
Sequence	: PAFHSWA-NH2
Lot No	: JT-91986
Pump A	: 0.1% Trifluoroacetic in 100% Water
Pump B	: 0.1% Trifluoroacetic in 100% Acetonrtrile
Total Flow	: 1ml/min
Wavelength	: 220nm
Analytial column type	: SHIMADZU Inertsil ODS-SP（4.6*250mm*5um）
Inj. Volume	: 30ul

Time	Module	Action	Value
0.00	Pumps	B.Conc	10
25.00	Pumps	B.Conc	70
25.01	Pumps	B.Conc	100
30.00	Pumps	B.Conc	100
30.01	Pumps	Stop	

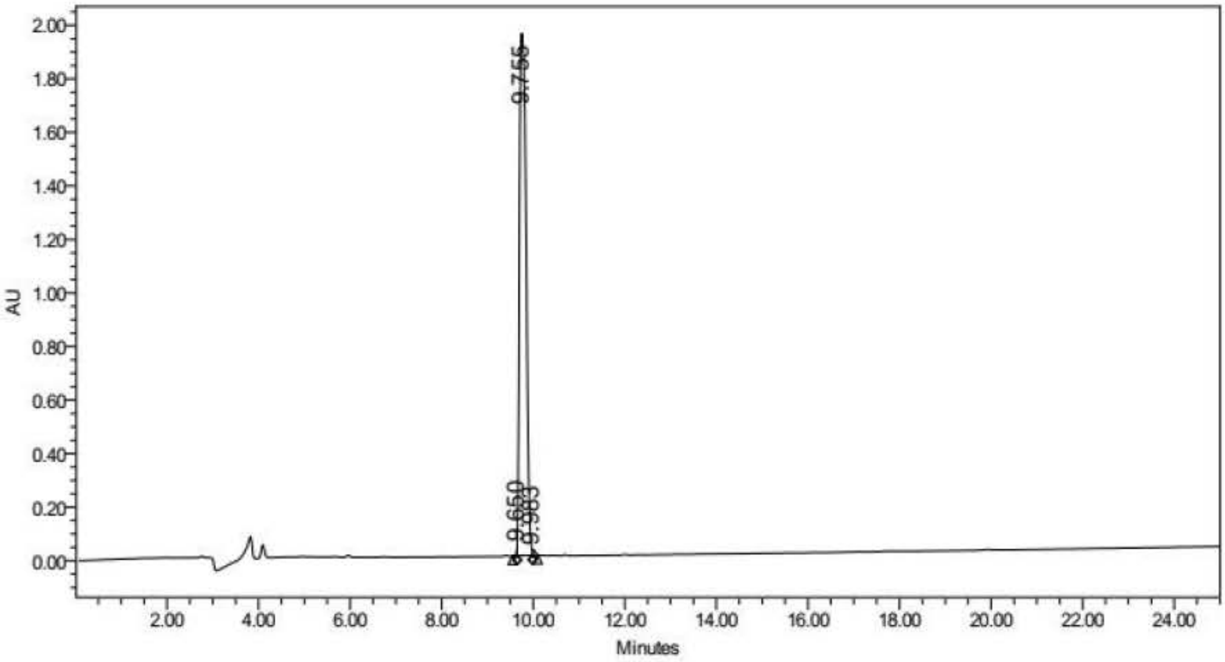

	RT	Area	% Area	Height
1	9.650	31496	0.16	37502
2	9.755	19518421	99.41	1961113
3	9.983	84277	0.43	20247

Synpeptide Co., Ltd

Address: 320 Lvlin Road, Beicai, Pudong new area, Shanghai, China 201204

Tel: +86-21-50835580 Fax: +86-21-50835580 P/C: 201204 http://www.synpeptide.com

ALK1_A7

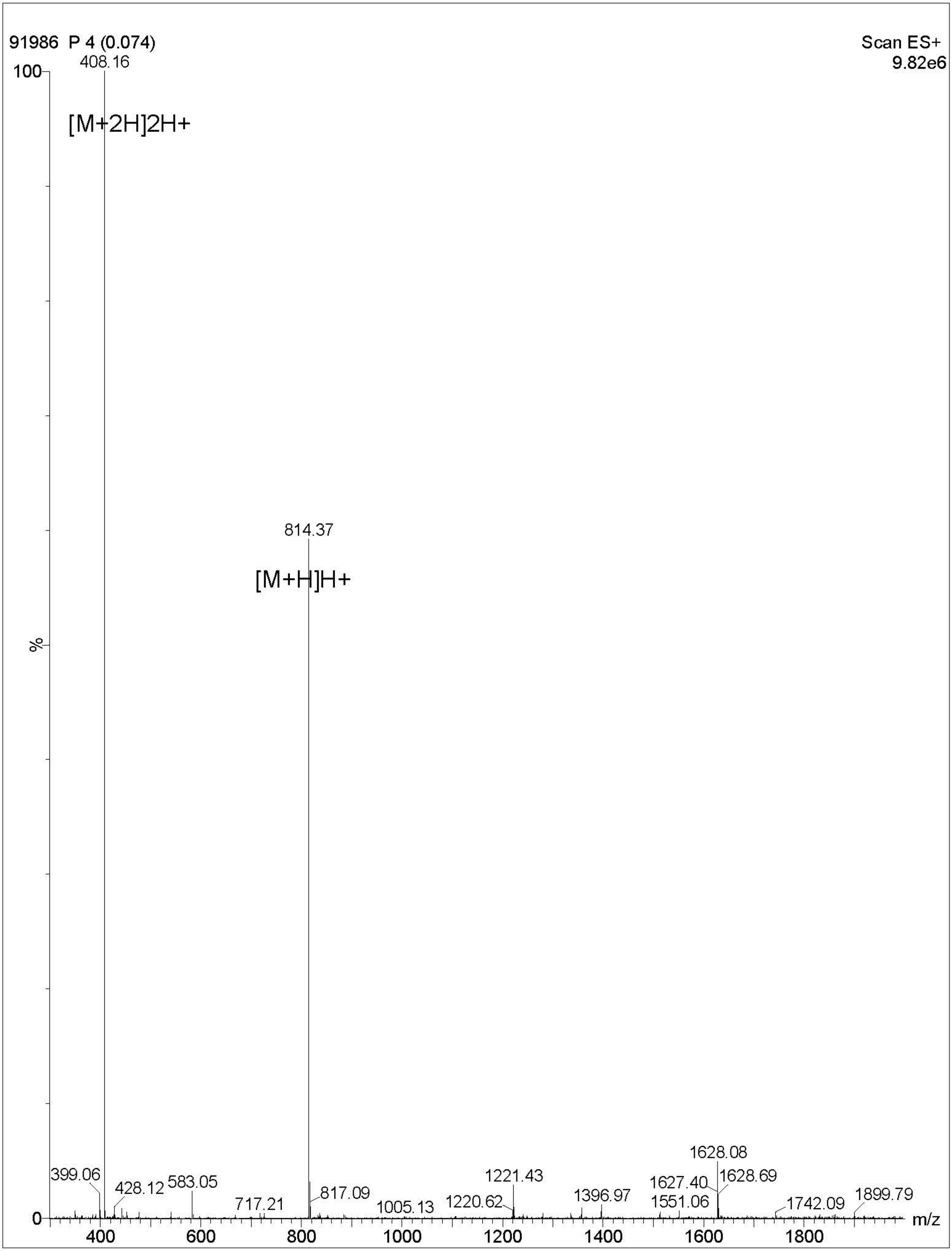

91986 P 4 (0.074)
Scan ES+
9.82e6
408.16
[M+2H]2H+
814.37
[M+H]H+
399.06
428.12
583.05
717.21
817.09
1005.13
1220.62
1221.43
1396.97
1551.06
1627.40
1628.08
1628.69
1742.09
1899.79
100
%
0
400
600
800
1000
1200
1400
1600
1800
m/z

ALK1-OH

CERTIFICATE OF ANALYSIS

**Product Name**	ALK1-nG
**Lot No**	JT-91980
**Sequence**	PAFHSWS
**Dissolution condition**	15%ACN+85%H2O
**Length**	7AA
**Modification**	N/A
**Molecular Weight (MW)**	830.88
**Storage**	-20℃

**Test Items**	**Specifications**	**Results**
**Purity by HPLC**	95%	98.81%
**Peptide Content**	N/A	N/A
**Moisture content**	N/A	N/A
**Acetic acid content**	N/A	N/A
**Appearance**	White to off-white lyophilized powder	Conforms
**Quantity**	2mg	2.0mg

Certified by:
Quality Assurance Department

Date 10-23-2020

Note: this product is intended for research use only; not for diagnostic or human use.

Synpeptide Co., Ltd

Address: 320 Lvlin Road, Beicai, Pudong new area, Shanghai, China 201204

Tel: +86-21-50835580 Fax: +86-21-50835580 P/C: 201204 http://www.synpeptide.com

ALK1-OH

Sample Information

Order ID	: Syn-91980
Name	: ALK1-nG
Sequence	: PAFHSWS
Lot No	: JT-91980
Pump A	: 0.1% Trifluoroacetic in 100% Water
Pump B	: 0.1% Trifluoroacetic in 100% Acetonrtrile
Total Flow	: 1ml/min
Wavelength	: 220nm
Analytial column type	: SHIMADZU Inertsil ODS-SP（4.6*250mm*5um）
Inj. Volume	: 30ul

Time	Module	Action	Value
0.00	Pumps	B.Conc	10
25.00	Pumps	B.Conc	70
25.01	Pumps	B.Conc	100
30.00	Pumps	B.Conc	100
30.01	Pumps	Stop	

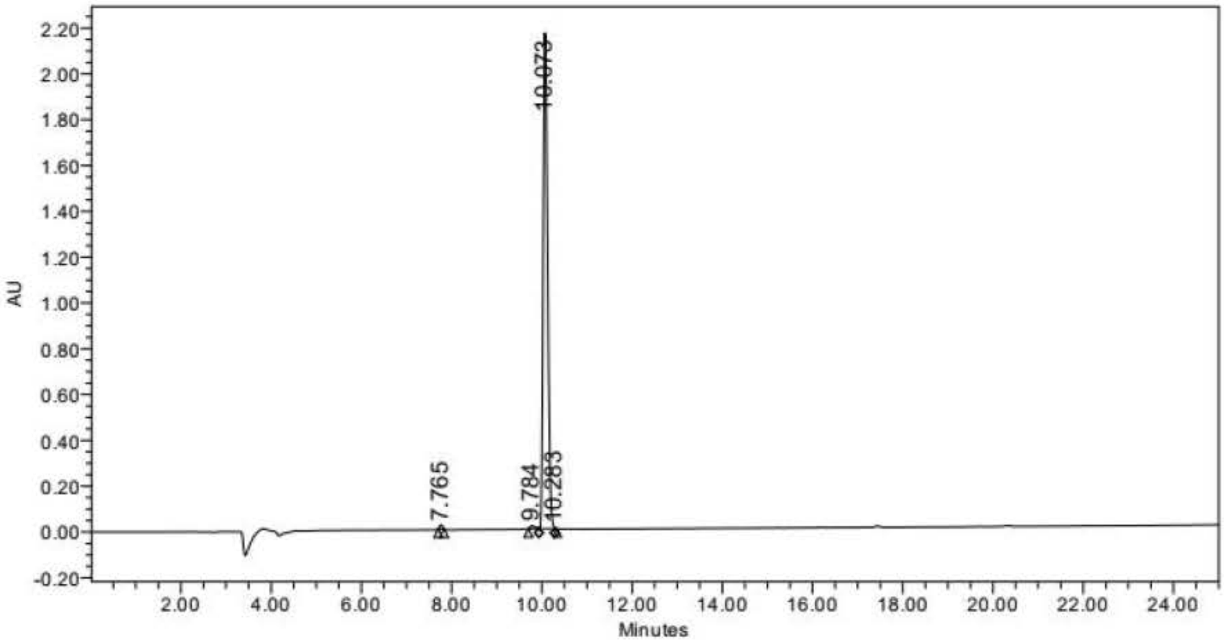

	RT	Area	% Area	Height
1	7.765	75220	0.50	16773
2	9.784	97347	0.65	13451
3	10.073	14719303	98.81	2178106
4	10.283	5300	0.04	4493

Synpeptide Co., Ltd

Address: 320 Lvlin Road, Beicai, Pudong new area, Shanghai, China 201204

Tel: +86-21-50835580 Fax: +86-21-50835580 P/C: 201204 http://www.synpeptide.com

ALK1-OH

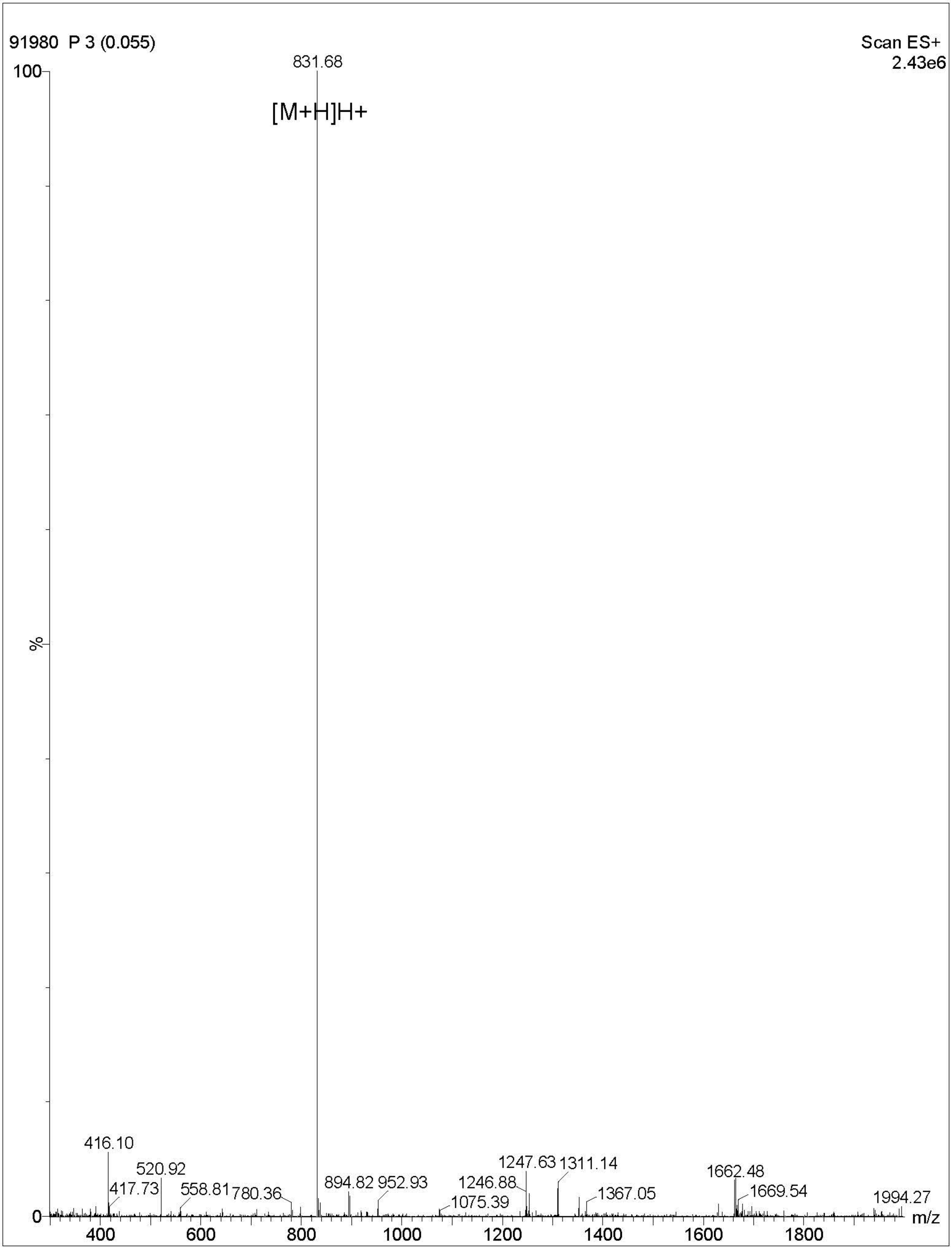

91980 P 3 (0.055)
Scan ES+
2.43e6
831.68
[M+H]H+
100
%
0
416.10
520.92
417.73
558.81
780.36
894.82
952.93
1075.39
1246.88
1247.63
1311.14
1367.05
1662.48
1669.54
1994.27
400
600
800
1000
1200
1400
1600
1800
m/z

CERTIFICATE OF ANALYSIS

**Order ID**	GP120451-2
**Name**	Anopheles 1
**Lot No.**	GP120451-2-0611
**Sequence**	DTPRYVSKQKFHSWG[amide]
**Dissolution condition**	100%H2O
**Length**	15AA
**Modification**	N/A
**Molecular Weight (MW)**	1835.04
**Storage**	-20°C

**Test Items**	**Specifications**	**Results**
**MW by MS**	1835.00	Conforms
**Purity by HPLC**	>95%	97.191%
**Peptide Content**	N/A	N/A
**Moisture content**	N/A	N/A
**Acetic acid content**	N/A	N/A
**Appearance**	White to off-white lyophilized powder	Conforms
**Quantity**	2mg	2.0mg

Certified by: LiuHui

Date 06/24/2021

Quality Assurance Department

Note: this product is intended for research use only; not for diagnostic or human use.

Guoping Pharmaceutical Co., LTD

地址:合肥市经开区桃花工业园拓展区工投立恒工业广场A2西F1,电话:0551-62841987 传真:0551-62841765 www.guopingyaoye.com

Sample Information

Order ID :GP120451-2
Name :Anopheles 1
Sequence :DTPRYVSKQKFHSWG[amide]
Lot.No :GP120451-2-0611
Pump A :0.1%Trifluoroacetic in 100% water
Pump B :0.1%Trifluoroacetic in 100% acetonitrile
Total Flow :1ml/min
Wavelength :220nm
Analytical column type :SHIMADZU Inertsil ODS-SP(4.6*250mm*5um)
Dissolution method :100%H2O
Inj. Volume :10 uL

Time	Module	Action	Value
0.01	Pumps	B.Conc	10
20.00	Pumps	B.Conc	50
23.00	Pumps	B.Conc	100
38.00	Pumps	B.Conc	100
40.00	Pumps	B.Conc	10
50.00	Controller	Stop	

Chromatogram

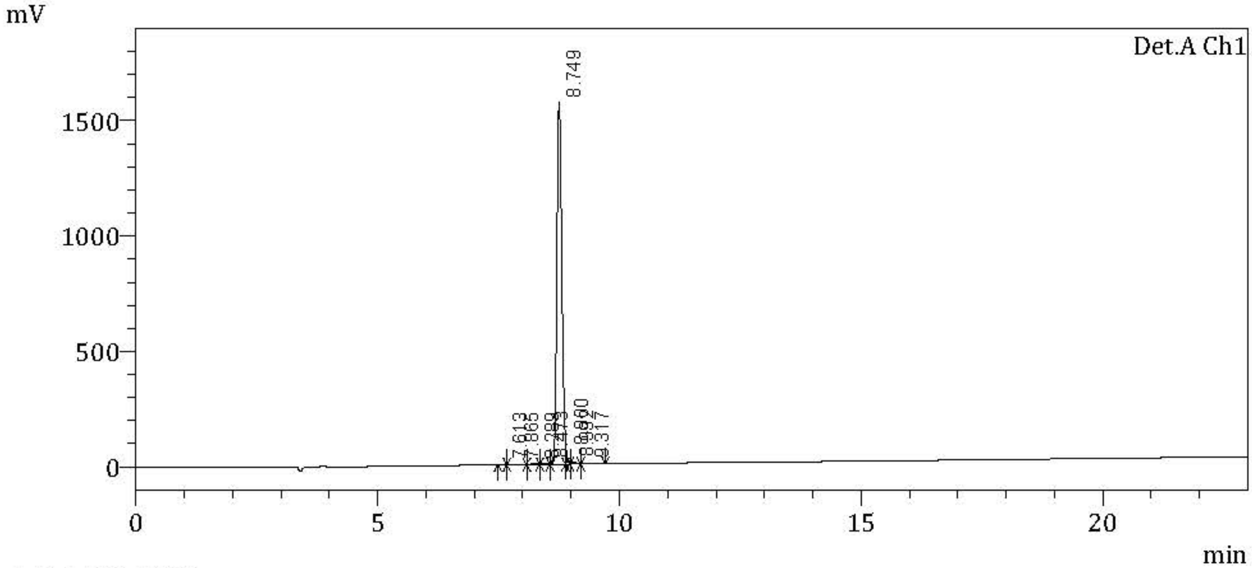

1 Det.A Ch1/220nm

PeakTable

Detector A Ch1 220nm

Peak#	Ret. Time	Area	Height	Area %	Height %
1	7.613	2088	301	0.018	0.018
2	7.865	31232	2653	0.273	0.161
3	8.289	26159	2247	0.229	0.136
4	8.473	45826	6091	0.401	0.370
5	8.749	11119628	1567012	97.191	95.117
6	8.900	135139	59027	1.181	3.583
7	8.992	62679	8481	0.548	0.515
8	9.317	18287	1644	0.160	0.100
Total		11441038	1647456	100.000	100.000

Anopheles LK1

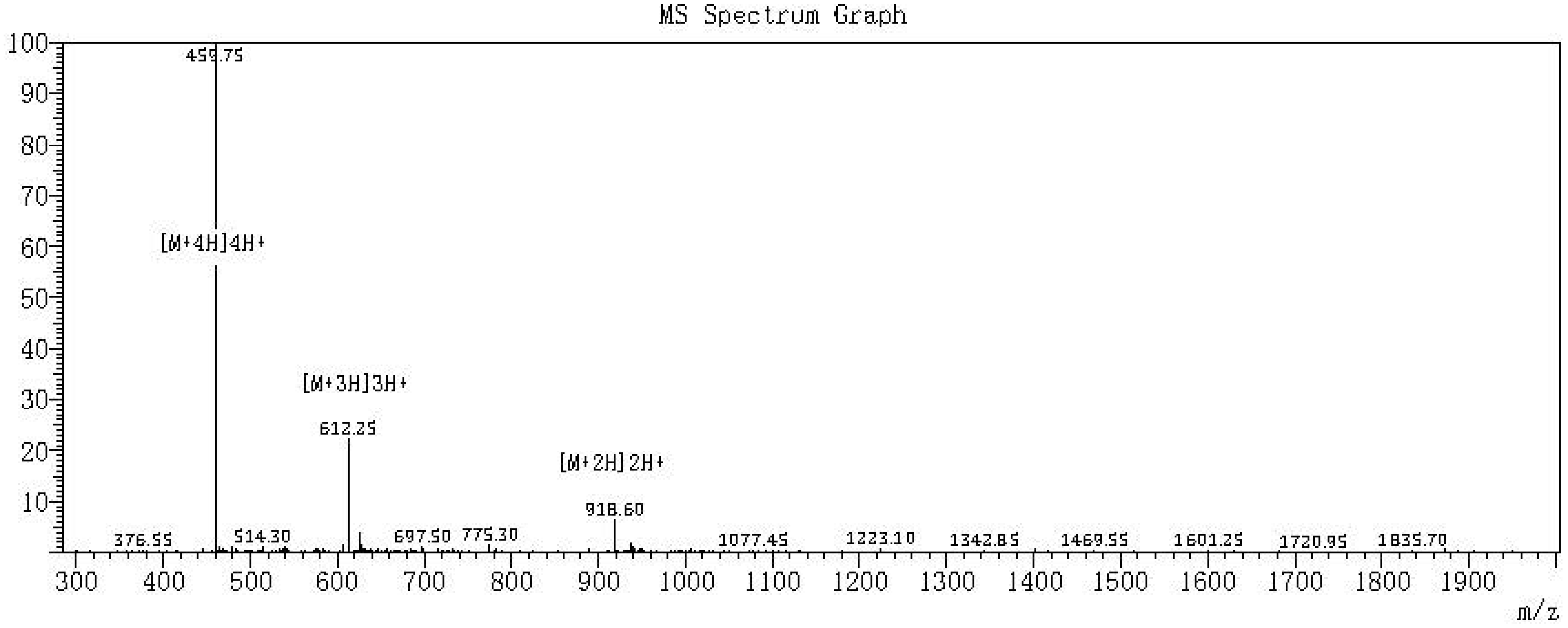

Sample Information

Dissolution method	:5%HAC+8%ACN+87%H2O	Interface	:ESI	Prerod Bias	:+1.5kv
Modified Date	:2021/06/24	Nebulizing Gas Flow	:1.50L/min	Detector	:-0.2kv
Injection Volume	:1ul	CDL Temp	:250C	T.Flow	:0.2ml/min
Heat Block Temp	:200	CDL Volt	:0v	B.conc	:50%H2O/50%MEOH

Order ID	:GP120451-2
Name	:Anopheles 1
Sequence	:DTPRYVSKQKFHSWG[amide]
Lot.No	:GP120451-2-0611
Theoretical	:1835.04
Observed	:1835.00

Certificate of Analysis

Gene Name	Anopheles LKR	Order No.	NJ0000288
Lot No.	Y28742/NJ0000288-NJ0000288-1	Cloning Vector	pCDNA3.1(+)
Cloning Sites	HindIII-ApaI	Insert Size	1734bp

QC Results

Test Items	Specifications	Results
Insert Sequence	Insert sequence results consistent with target	Pass
Vector Sequence	Flanking sequence consistent with expected	N/A
ORF Across Junction	Correct and consistent with target	N/A
Restriction Digest	Expected fragment sizes observed	Pass
PCR Amplification	Correct without non - specific bands	N/A
DNA Quantity/Quality	Actual yield (by A 260)	4ug
	Concentration (n/a if lyophilized)	N/A
	Purity (A 260/A280 = 1.8 - 2.0)	Pass
	# of Tubes	1
	Matrix	ddH2O
Endotoxin Test	Verified, <0.1 EU/μg (Endo-Free Preps Only)	N/A
Appearance	Clear, no visible particles	Pass
Label	Correct and white	Pass
Comments	NA	

Restriction Digestion Map

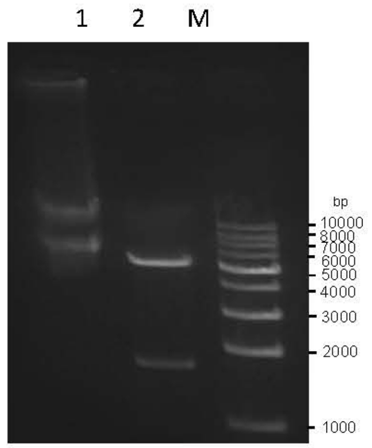

Lane1: plasmid DNA

Lane2: Digested with HindIII/ApaI

Lane M: DNA Marker

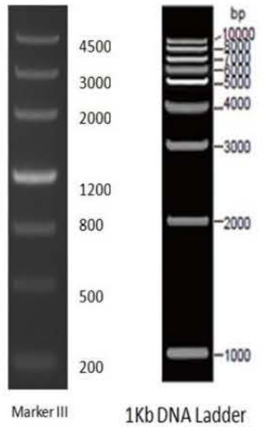

Certified by: Jie Chen	Date:20210611
**Plasmid Map and Gene Sequence**	
**Gene Name**：Anopheles LKR	
**Order No**：NJ0000288-NJ0000288-1	**Date**：20210611
**Vector Name**：pCDNA3.1(+)	**Clone site**：HindIII-ApaI
The gene Anopheles LKR has been cloned intopCDNA3.1(+) by HindIII-ApaI	

Plasmid Map

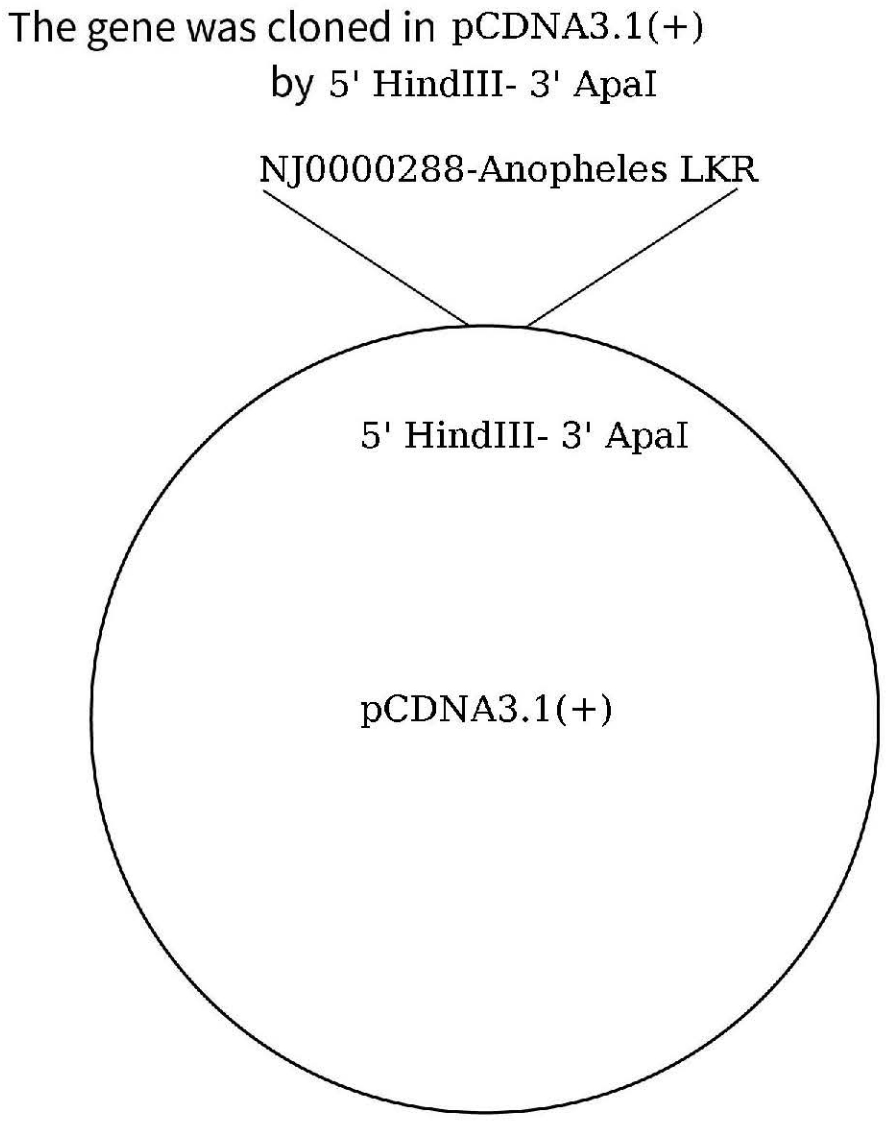

Gene Sequence：

aagcttATGCAAGCAACAGATATCACGGCATATCACACTGCCTACAACTACACACTCAACCAGTCGGACGTAAG
GATTGTGCTGGAGGATGAAAACCTTTACAAAGTCCCCATCGGCTTGCTGGTGCTGCTGTCCATCTTTTACGG
CACCATCAGCATCCTGGCGGTGATCGGGAACTCGCTAGTCATATGGATCGTCATCACGACCAAGCAGATGCA
GACGATCACCAACATGTTCATCGCCAACCTGGCCCTGGCGGACGTGACGATCGGTGTGTTTGCAATCCCATT
CCAGTTTCAGGCAGCGCTACTGCAACGCTGGAACCTGCCCGAGTTTATGTGTCCGTTCTGCCCGTTCGTGCA
GCTTATCAGCGTGAACGTGTCCGTCTTCACGCTGACTGCCATTGCAGTCGACAGGCATCGGGCCATCATCAA
TCCGCTCAGAGCACGAACATCGAAAAACATCTCCAAGTTCGTCATCAGCAGCATCTGGATGCTATCGTTTGTG
CTGGCCGCCCCGATACTGTTTGCGCTTCGAGTGCGGCCCGTGTCCTACATTGCGCTGGGTGGCATGAACGAT
ACGTACACCAACATCACCGTACCGTTCTGTAAGGTGGTCAACTTTGAGGATGGAGAGATTCTGCTCTATCGCT
ACGTGCTCGTGCTAGTGCAGTACTTCATACCGCTGTTCGTCATCAGCTTCGTTTACATACAGATGGCCCTCCG
GCTCTGGGGCTCGAAGACACCGGGCAATGCGCAAGACTCGCGGGACATTACCATGCTGAAGAACAAGAAG
AAGGTTATCAAAATGCTGATCATCGTTGTGGCGCTGTTCGGCGTGTGCTGGTTTCCACTGCAGCTTTACAACA
TCCTGCACGTGACGTGGCCGGAAATCAATGAATATCGCTTTATCAACATCATTTGGTTCGTGTGTGATTGGCT
TGCGATGAGCAACAGCTGTTACAATCCGTTTATCTACGGCATTTATAATGAAAAGTTTAAGCGCGAATTTCGC
AAACGTTACCCGTTCAAGCGCGATCAAACGTACAACCACAACCACGAGTCGGATAAAACGTCATCGATTTTT
ACGCGCGTCAGTTCCATCCGCTCGACCTACGCCACATCGTCCATACGGAACAAGCTAAGCACTAACCGGTAC
TCGGCATCGAAGCAATTCAAATTCCCGCCACCCAACCATCATTTCCAGCACCAGCCGGGTGGCCATCACAAC
GCCACCGGGGGTGCCCATCTGCACGAGCTAGCGTTCGGAACGTCCAAGAAGGGGCCAGTTAATTTTGACG
GCACGGTGACGACCACCTTCGCCACGAACCATCCGAGGGAAAAGAAGATGGATCACCGTTTGGTTGAGCA
CGACCAGCTGATTGCATCGTGCATCGAACGGCTCGACCATGAGCTCGCGTGTTCCAGCACGGTGGACAGTA
GTGAGGATCACCGAAACGGCGAACCGAGGACGCTCAACCGACCGGATATCGATGGAAATGGTACGGGACG
AGCTGCGAAACTTCGCAACGGTTCATCCCGGGAATGTGGGCTCTCCATCGCGAGCAATTACGCTGACCGGA
TGGCACTGAAGCATCCCCATCCGGACAGTGGTGGGGAGAGTGGCGATGGGGAACCGAAACCGGGACAGC
GCTCCAGCGAGGAACGGGACTCGGGCGGACACTTGTACTGTAACGATTTGGAAGAACTCGGACCGTACTAT
GATTAAgggccc

Certificate of Analysis

Product Name: 04010026651

Sequence: GFRLNSASRVAHGY-NH2

Sequence(Three Letters Code): Gly-Phe-Arg-Leu-Asn-Ser-Ala-Ser-Arg-Val-Ala-His-Gly-Tyr-NH2

Purity: 99.52%

Molecular Weight: 1533.73

Solubility: 1mg/ml in 33%ACN/67%H_2O

Test	Specification	Result
**Purity:**	HPLC	Conforms
	(See attached RP-HPLC chromatogram)	
**MS Analysis:**	ESI-MS	Conforms
	(See attached MS spectrum)	
**Counter Ion:**	Trifluoroacetate	Conforms
**Appearance:**	Lyophilized powder or Crystallization	Conforms

Quality Assurance By: ______________ **Position**: Manager **Date**: 2018-12-24

Important: The peptides can be used for research only. Most of the peptides are lyophilized white or faint yellow powder while fluorescent modified ones have special colors. The state of peptides with strong hydrophilic properties may be crystalline or liquid which does not affect for use. Before experiment, please choose proper solvent for your experiment to dissolve peptides. If peptides cannot be dissolved under harsh conditions, we can carry out feasibility study. Storage conditions:-20℃, seal, avoid light, dry.

Please test the sample within two weeks after receiving it.

注意：本品仅供科研，生产用途，不得直接用于人体。

HPLC Analysis Report

Measurement:	Peak Area	Run Time:	20min
Calculation Type:	Percent	Wavelength :	220nm
Flow Rate :	1.0ml/min	Inj.Vol:	10uL
Column:	Kromasil 100-5C18,4.6mmX250mm,5 micron	Column Temp:	25℃
Buffer A :	0.1%TFA in Acetonitrile	Buffer B:	0.1%TFA in water

Gradient(linear):		A	B
	0.0min	15%	85%
	20min	50%	50%
	20.1min	100%	0%

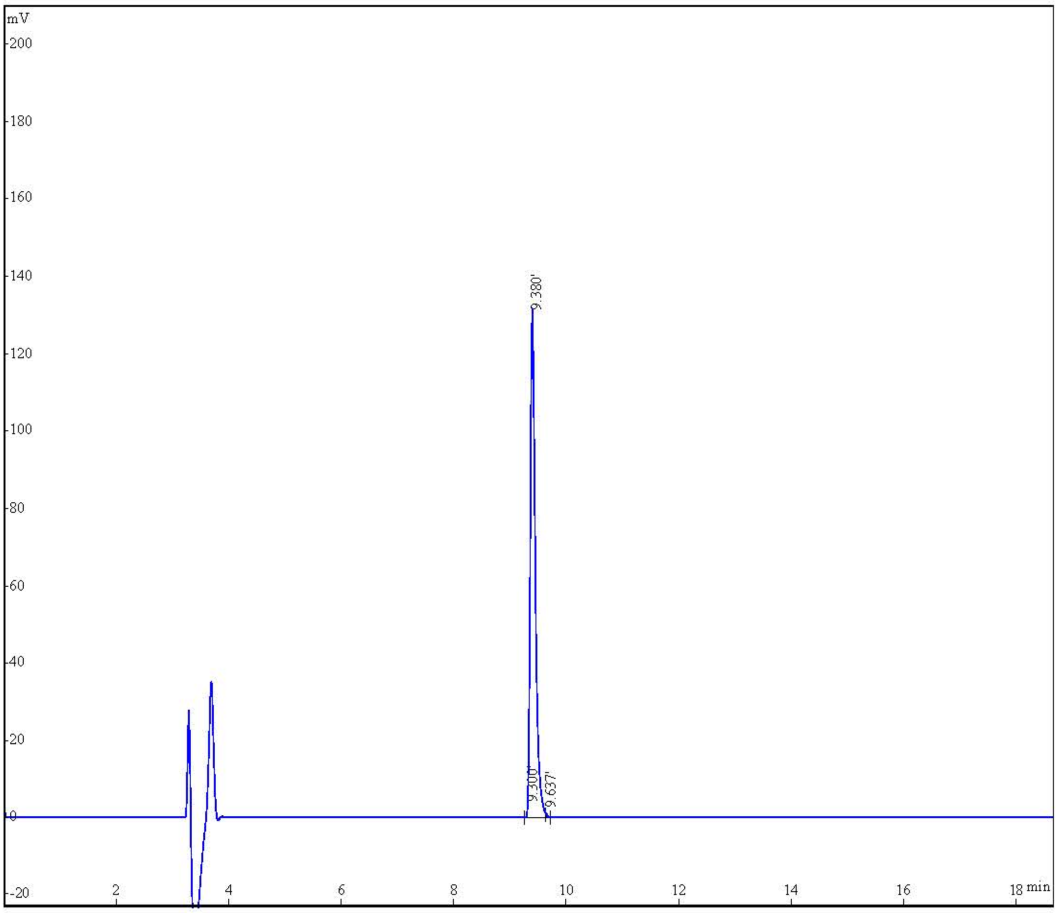

Rank	Time	Name	Conc.	Area
1	9.300		0.2746	2263
2	9.380		99.52	820427
3	9.637		0.1982	1634
Total			100	824324

MS Analysis Report

Ion Source: ESI Nebulizer Gas (NEB): 12.00
Curtain Gas(CUR): 6.00 Ionspray Voltage(IS): ±4500
Temperature(TEM): 0.00 Run Time: 0.5-1min

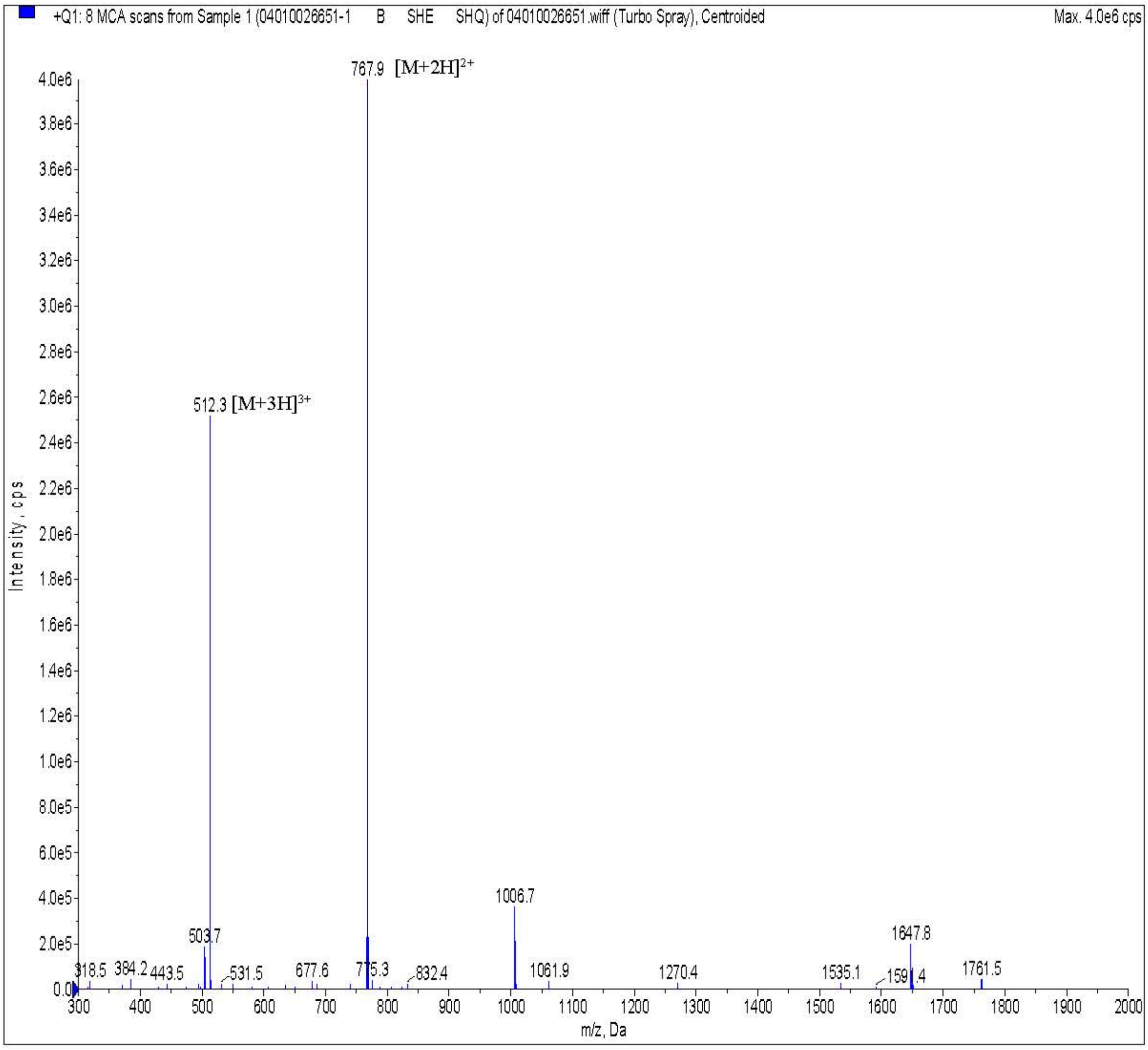

Supplement: Figure S15 [file mmc2.pdf]
